# Metal–ligand Lability and Ligand Mobility Enables Framework Transformation via Ligand Release in a Family of Crystalline 2D Coordination Polymers

**DOI:** 10.1002/chem.202201408

**Published:** 2022-07-19

**Authors:** Feifan Lang, Daljit C. N. G. Singh, Abhishek B. Rao, Catherine Romer, James S. Wright, Rebecca Smith, Harry Adams, Lee Brammer

**Affiliations:** ^1^ Department of Chemistry University of Sheffield Brook Hill Sheffield S3 7HF UK; ^2^ Current address: Department of Chemistry University of Surrey Guildford Surrey GU2 7XH UK; ^3^ Current address: Eos Energy Enterprises Edison NJ 08820 USA

**Keywords:** coordination polymer, crystal engineering, dielectric constant, in situ X-ray diffraction, solid-state reaction

## Abstract

A family of seven silver(I)‐perfluorocarboxylate‐quinoxaline coordination polymers, [Ag_4_(O_2_CR_F_)_4_(quin)_4_] **1**–**5** (R_F_=(CF_2_)_n‐1_CF_3_)_4_, n=1 to 5); [Ag_4_(O_2_C(CF_2_)_2_CO_2_)_2_(quin)_4_] **6**; [Ag_4_(O_2_CC_6_F_5_)_4_(quin)_4_] **7** (quin=quinoxaline), denoted by composition as *4 : 4 : 4 phases*, was synthesised from reaction of the corresponding silver(I) perfluorocarboxylate with excess quinoxaline. Compounds **1**–**7** adopt a common 2D layered structure in which 1D silver‐perfluorcarboxylate chains are crosslinked by ditopic quinoxaline ligands. Solid‐state reaction upon heating, involving loss of one equivalent of quinoxaline, yielding new crystalline *4 : 4 : 3 phases* [Ag_4_(O_2_C(CF_2_)_n‐1_CF_3_)_4_(quin)_3_]_n_ (**8**–**10**, n=1 to 3), was followed in situ by PXRD and TGA studies. Crystal structures were confirmed by direct syntheses and structure determination. The solid‐state reaction converting *4 : 4 : 4* to *4 : 4 : 3 phase* materials involves cleavage and formation of Ag−N and Ag−O bonds to enable the structural rearrangement. One of the *4 : 4 : 3 phase* coordination polymers (**10**) shows the remarkably high dielectric constant in the low electric field frequency range.

## Graphical abstract

**Two‐dimensional coordination** polymers exhibit ligand extrusion into the vapour phase on heating to undergo an extensive structural reorganisation involving bond breaking and formation, ligand migration and reorientation. The reactions occur with retention of crystallinity and have been followed in situ by PXRD.

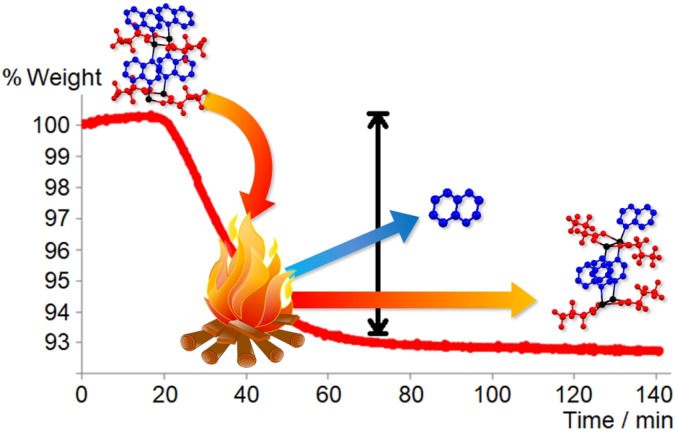

## Introduction

Metal–organic frameworks (MOFs) and the broader category of coordination polymers (CPs) are a structurally, compositionally and functionally highly varied and versatile class of materials assembled through coordination bonds between metal centres and di‐ or polytopic linking ligands.^[1]^ The use of labile metal ions facilitates reversible metal–ligand bond formation which is advantageous in error‐correction during formation of the periodic coordination network via self‐assembly. The metal–ligand bonds are, however, typically the weakest point of the network and often dictate the limitations of a given material in chemical or thermal stability. This has led, for example, to more recent extensive development of MOFs containing kinetically inert high‐oxidation‐state metal ions (Cr^3+^, Fe^3+^, Zr^4+^, Hf^4+^) to enhance stability.^[2]^

Retaining some “weakness” or kinetic lability of metal–ligand bonding, however, can open up new opportunities for structural and chemical transformations in the solid state and more generally for dynamic behaviour of these materials.^[3]^ Solid‐state transformations may be induced by external stimuli, such as temperature, pressure, light, field, etc. Of particular interest in the context of the work presented herein are solid‐state transformations that involve changes in metal–ligand coordination. These often include either uptake, removal or displacement of molecular species that serve as ligands in one form of the material and are disconnected from metal centres in an alternative form. Thus, metal centres can change in coordination number and/or coordination geometry enabling formation of new materials via solid‐state routes.^[4]^ Such transformations can also be considered a form of post‐synthetic modification (PSM), although PSM more often focuses on organic transformations of linker ligands.^[5]^

Silver(I)‐containing coordination polymers (AgCPs) are a well‐established class of compounds often involving N‐ and O‐donor ligands that form coordinative bonds with Ag(I) in variety of combinations of coordination number and geometry.^[6]^ In addition, argentophilic interactions^[7]^ can form between Ag(I) centres in close proximity. The versatility in coordination environment in AgCPs provides an ideal platform for studies of solid‐state reactions involving metal–ligand bond breaking and/or formation, leading to new materials. We have developed a class of AgCPs that exploit such behaviour and are based upon Ag(I)‐perfluorocarboxylate units, either discrete or extended, that are linked by diimine or diamine ligands.[^8^, ^9^, ^10^, ^11^] These exhibit facile formation as crystalline materials and the versatile deformation of the Ag(I) coordination environment has enabled a variety of processes to be monitored by diffraction methods due to retention of crystallinity. These processes include ligand insertion and removal,^[9]^ changes in network dimensionality[^9^, ^10^] and selective entrapment of guests such as xylenes and other aromatic molecules.^[11]^

In the present study we extend this class of materials to a new family in which quinoxaline (quin) is deployed as a diimine linker to form coordination polymers with a series of Ag(I) perfluorcarboxylates, [Ag_4_(O_2_CR)_4_(quin)_4_] (**1**–**5**: R=(CF_2_)_n‐1_CF_3_)_4_, n=1 to 5; **6**: R=CF_2_CF_2_COO; **7**: R=C_6_F_5_; quin=quinoxaline). We denote these materials by composition as *4 : 4 : 4 phases* (Ag : O_2_CR_F_ : quin) and show by single‐crystal X‐ray diffraction that these are layered 2D coordination polymers with perfluorinated interlayer regions. Choice of perfluoroalkyl or ‐aryl group enables control of the interlayer spacing and thermal stability. Upon heating, selected coordination bonds break resulting in these materials losing 25 % of quinoxaline ligands and enabling transformation via formation of new coordination bonds into a new family of coordination polymers, the *4 : 4 : 3 phases* [Ag_4_(O_2_C(CF_2_)_n‐1_CF_3_)_4_(quin)_3_]_n_ (**8**–**10**, n=1 to 3), which we have studied for selected perfluorocarboxylates. These transformation processes have been monitored in situ by thermal analysis and PXRD and the structures of the 4 : 4 : 3 phases have been verified by single‐crystal X‐ray diffraction, enabling a solid‐state mechanistic pathway to be proposed.

## Results and Discussion

### Crystal structures and control of spatial dimensions

**Figure 1:**
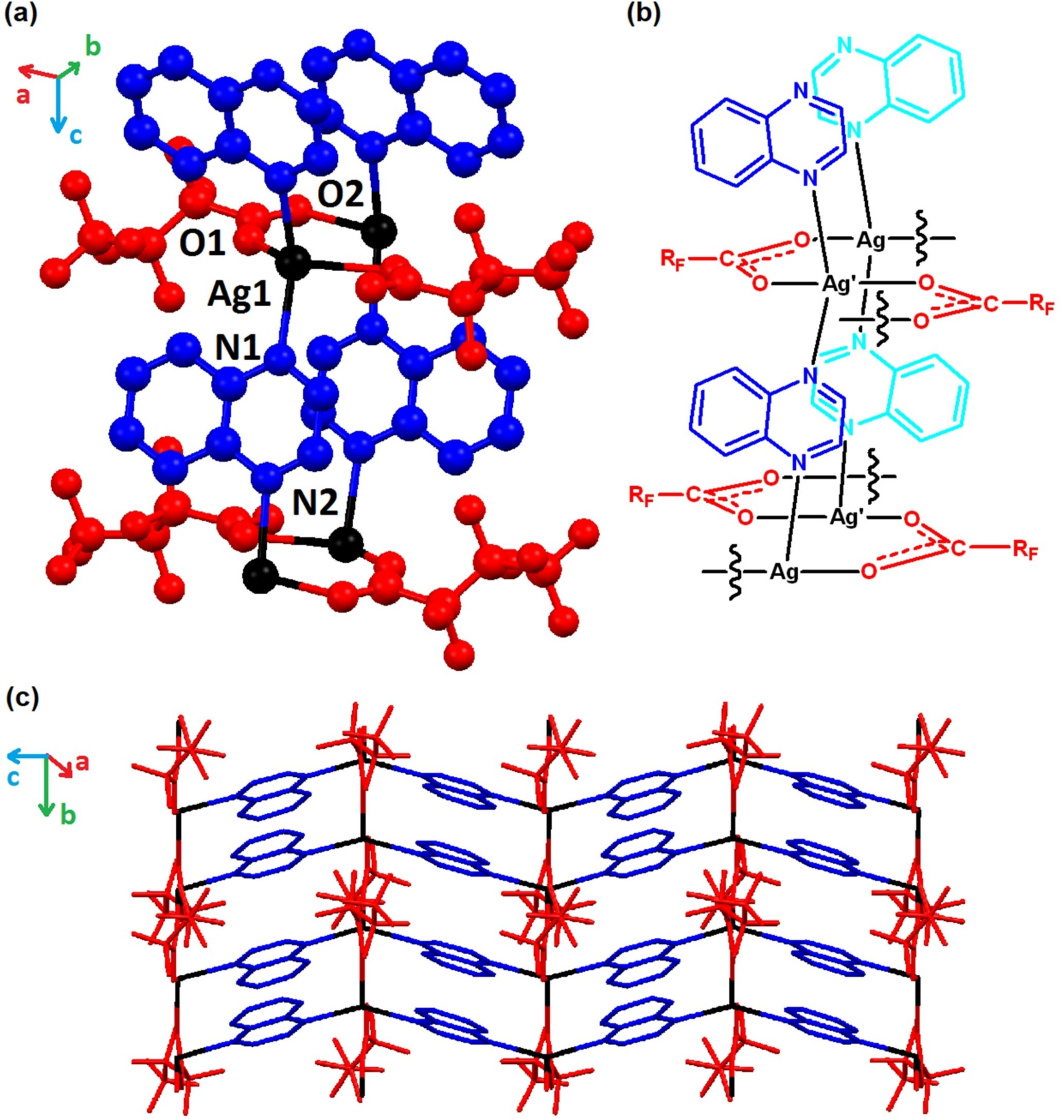
The flattened tetrahedral Ag(I) coordination environment and network propagation for **3** shown (a) as its crystal structure and (b) schematically. (c) A single layer of the crystal structure viewed perpendicular to the (100) plane showing orthogonal propagation of the network via bridging perfluorcarboxylate and quinoxaline groups along the *b*‐ and *c*‐axis directions, respectively.

Compounds **1**–**7** (the 4 : 4 : 4 phases) were synthesised by reaction of a slight excess of quinoxaline with the corresponding silver(I) carboxylate salt (**1**‐**5**) or by reaction of quinoxaline with the product of reaction of Ag_2_O with the corresponding carboxylic acid (**6**, **7**). Phase purity was confirmed by elemental analysis and/or Pawley fitting of PXRD data. This series of coordination polymers adopt a common 2D layered structure, as illustrated for compound **3** in Figure 1. Each Ag centre adopts a flattened tetrahedral geometry and is coordinated by N‐atoms from two quinoxaline ligands (N−Ag−N 148.3(1)° in **3**) and O‐atoms from two carboxylate groups (O−Ag−O 122.2(1)° in **3**). Quinoxaline ligands bridge between Ag centres to propagate a zigzag chain along the *c*‐axis, whereas carboxylate ligands connect neighbouring Ag centres in a μ:κ^1^,κ^1^‐bridging mode to propagate a coordination network along the *b*‐axis. The 2D network layers therefore lie parallel to the (100) planes.

**Figure 2:**
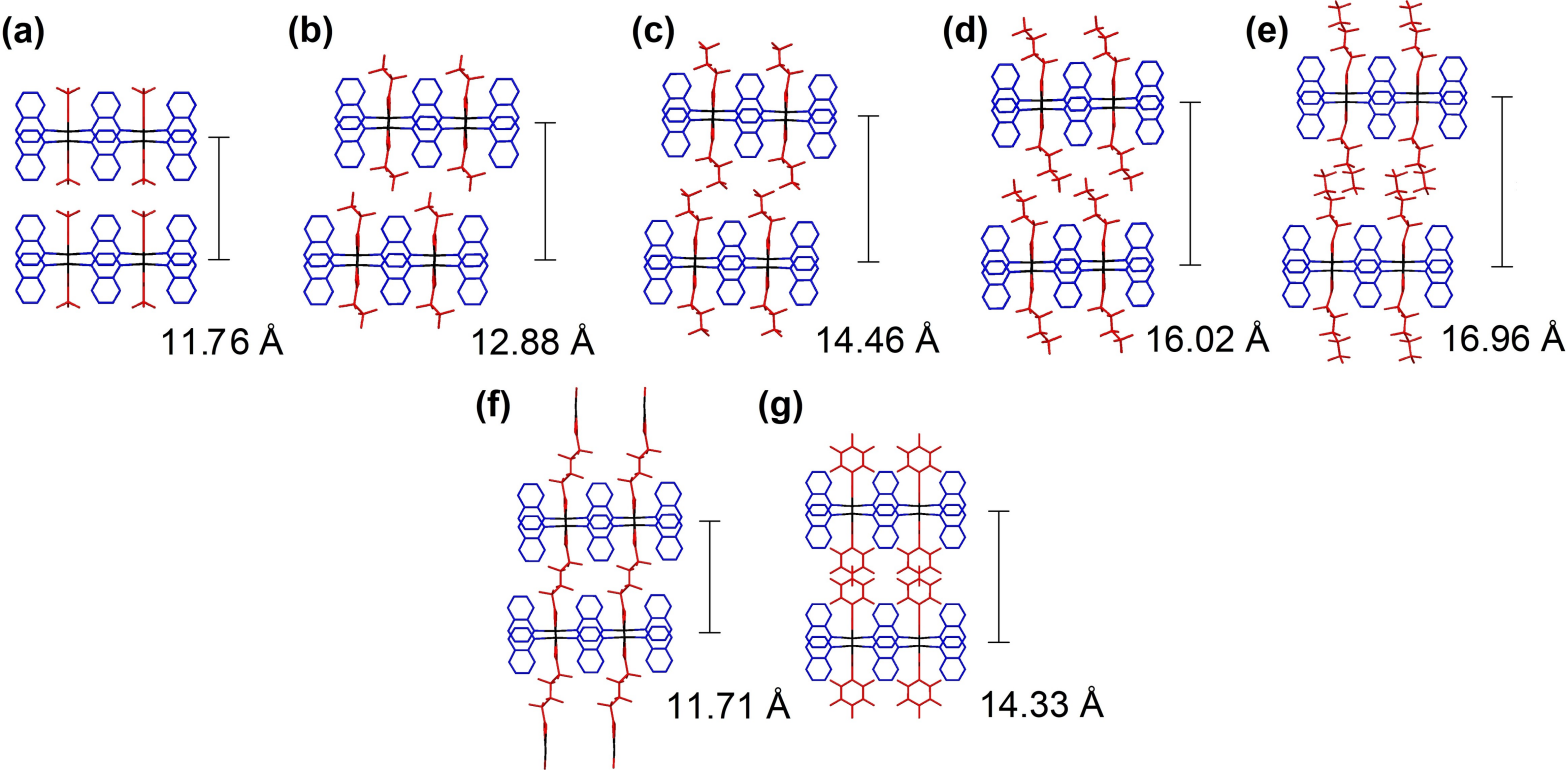
(a–g) Crystal structures of 4 : 4 : 4 phase coordination polymers **1**–**7** noting their interlayer spacings. (a–e) show perfluoroalkylcarboxylates **1**–**5**, (f) shows layers linked via a perfluoroalkyldicarboxylate (**6**) and (g) shows perfluorophenylcarboxylate (**7**).

The shortest distance between the Ag centres (along the Ag‐carboxylate network) lies in the range 3.38–3.48 Å, which is close to the minimum reported Ag⋅⋅⋅Ag van der Waals separation (3.44 Å),^[12]^ suggesting any argentophilic interaction is likely to be very weak. The quinoxaline ligands from adjacent chains are aligned face‐to‐face and anti‐parallel, with an interplanar distance of approximately 3.52 Å, indicating reasonably strong π⋅⋅⋅π stacking interactions. Neighbouring layers of the coordination polymer network are stacked along the [100] direction with interlayer spacings in the range 11.7–17.0 Å dependent on the size of the perfluorinated R‐group of the carboxylate (Figure 2). There is a monotonic expansion in the interlayer spacing across the series of perfluoroalkylcarboxylates (compounds **1**–**5**). Perfluoroalkyl chains interdigitate in the interlayer region in a manner similar to that observed in other layered silver‐perfluoroalkylcarboxylate‐diimine coordination polymers in which these perfluoroalkyl‐rich regions have been shown to enable small‐molecule guest transport via chain mobility.^[9]^ It is interesting to note that when using pentafluorophenylcarboxylate (**7**), the aromatic rings display offset π‐stacking in the interlayer region leading to an interlayer separation similar to that when perfluorobutanoate (**3**) is used. Use of a dicarboxylate, octafluoroadipate, enables adjacent layers of the coordination polymer to be covalently linked in **6** and leads to an interlayer spacing slightly shorter than observed for the shortest monocarboxylate used (CF_3_CO_2_^−^ in **1**).

**Figure 3:**
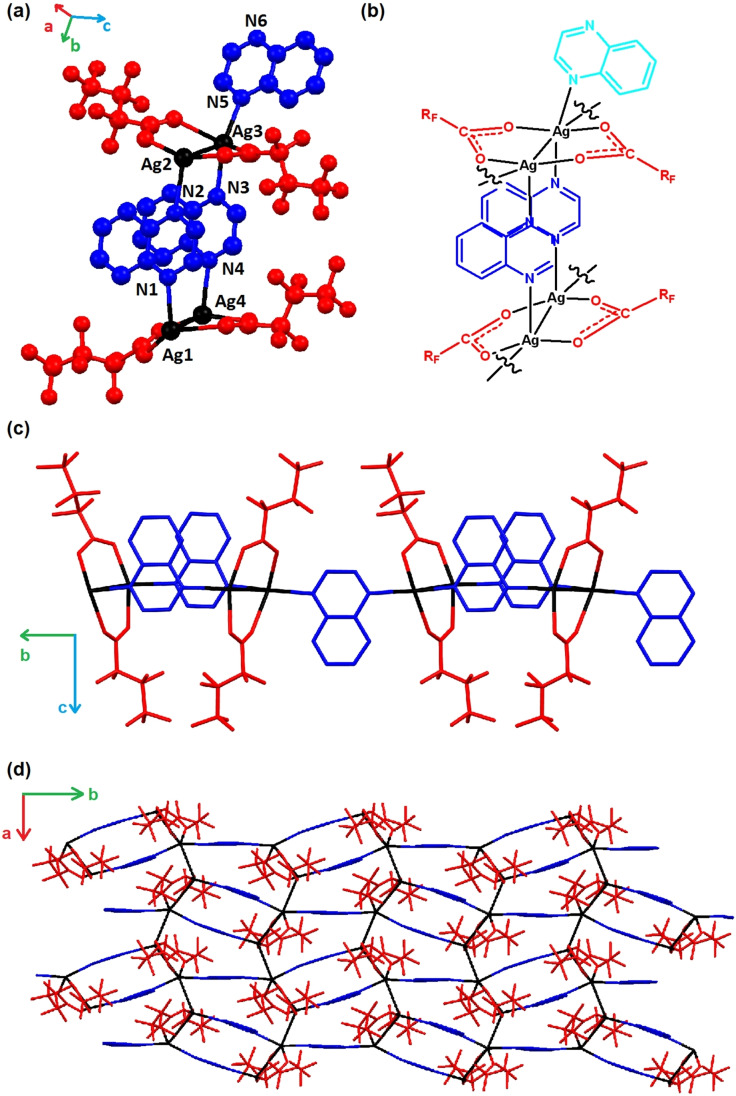
The Ag(I) coordination environments and network propagation for **10** shown (a) as its crystal structure and (b) schematically. (c) Zig‐zag tape arrangement showing propagation of coordination polymer via alternating single and double quinoxaline bridges between Ag_2_(O_2_CR_F_)_2_ units. (d) A single layer of the crystal structure viewed perpendicular to the (001) plane showing orthogonal propagation of the network via bridging quinoxaline ligands and argentophilic Ag⋅⋅⋅Ag interactions along the *b*‐ and *a*‐axis directions, respectively.

**Figure 4:**
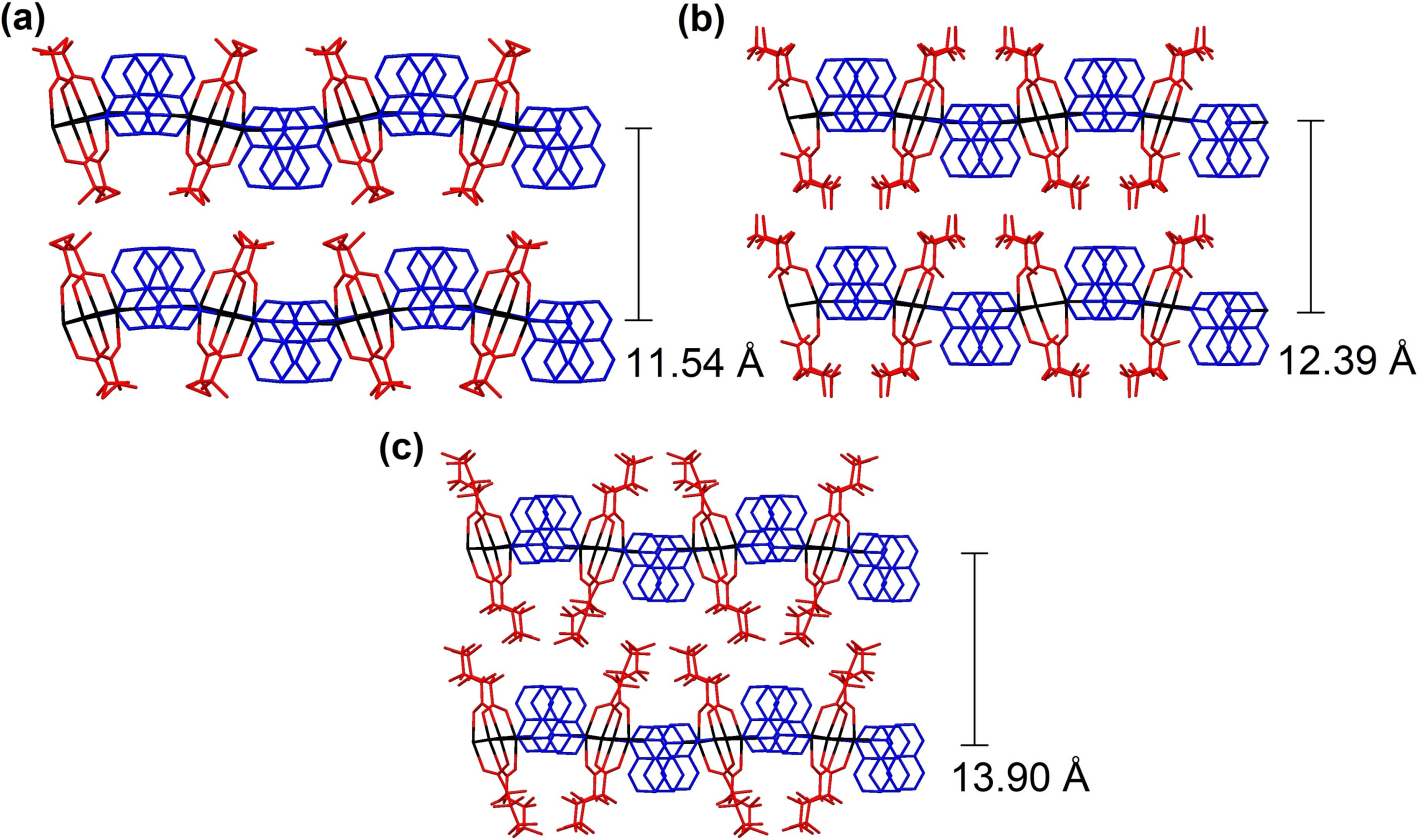
Crystal structures of 4 : 4 : 3 phase coordination polymers (a) **8**, (b) **9**, (c) **10** showing interlayer dimensions and perfluoroalkyl chain interdigitation.

Adjusting the stoichiometry of the reaction from using a small excess of quinoxaline to prepare the *4 : 4 : 4 phases* (**1**–**7**), to one in which the silver(I) carboxylate is in excess, enabled preparation of a new series of coordination polymers [Ag_4_(O_2_C(CF_2_)_n‐1_CF_3_)_4_(quin)_3_]_n_ (**8**–**10**, n=1 to 3), which are analogues of compounds **1**–**3**, but with a 4 : 4 : 3 ratio of Ag : O_2_CR_F_ : quin, henceforth denoted as *4 : 4 : 3 phases*. Crystal structures were determined by single‐crystal X‐ray diffraction and phase purity was confirmed by elemental analysis and Pawley fitting of PXRD data. Like their 4 : 4 : 4 counterparts, the 4 : 4 : 3 phase materials are 2D coordination polymers with a layered structure (layers parallel to the (001) planes) with interlayer regions comprising interdigitated perfluoroalkyl chains (Figures 3 and 4). The absence of 25 % of the quinoxaline ligands, however, leads to a different arrangement within the layers. Perfluorocarboxylate ligands (O_2_CR_F_) are still coordinated to Ag(I) centres in a μ: κ^1^, κ^1^‐bridging manner, but now form discrete Ag_2_(O_2_CR_F_)_2_ dimers (Ag⋅⋅⋅Ag 3.065–3.194 Å) rather than infinite {Ag(O_2_CR_F_)}_n_ chains.^[13]^ The coordination polymer is propagated within each layer via an alternating arrangement of double and single quinoxaline ligand bridges linking these Ag_2_(O_2_CR_F_)_2_ units into a zigzag tape along the *b*‐axis, with the tapes cross‐linked via short unsupported argentophilic interactions (Ag⋅⋅⋅Ag 3.088–3.167 Å) along the *a*‐axis, as illustrated by **10** in Figure 3. This leads to two distinct Ag(I) coordination environments. One is analogous to that in the 4 : 4 : 4 phase materials, i. e. flattened tetrahedral coordination (Ag1 and Ag3, Figure 3) involving two quinoxaline ligands (N−Ag−N 149.8(3)–153.5(4)° in **10**) and two carboxylates (O−Ag−O 124.3(3)–125.5(3)° in **10**). The other is a distorted T‐shaped coordination (Ag2 and Ag4, Figure 3) involving one quinoxaline ligand and two carboxylates.

Pairs of quinoxaline ligands in the double bridges are oriented in a parallel face‐to‐face manner with an interplanar distance of approximately 3.5 Å. The singly bridged ligands along the same zigzag tape are oriented antiparallel to the doubly bridged ligands (Figure 3). Neighbouring layers for **8**–**10** have interlayer spacings of 11.5–13.9 Å. As seen for the 4 : 4 : 4 phases, there is a correlation of increased separations with increasing perfluoroalkyl chain length, although in each case separations are slightly shorter than in the corresponding 4 : 4 : 4 phases (Figure 4).

**Figure 5:**
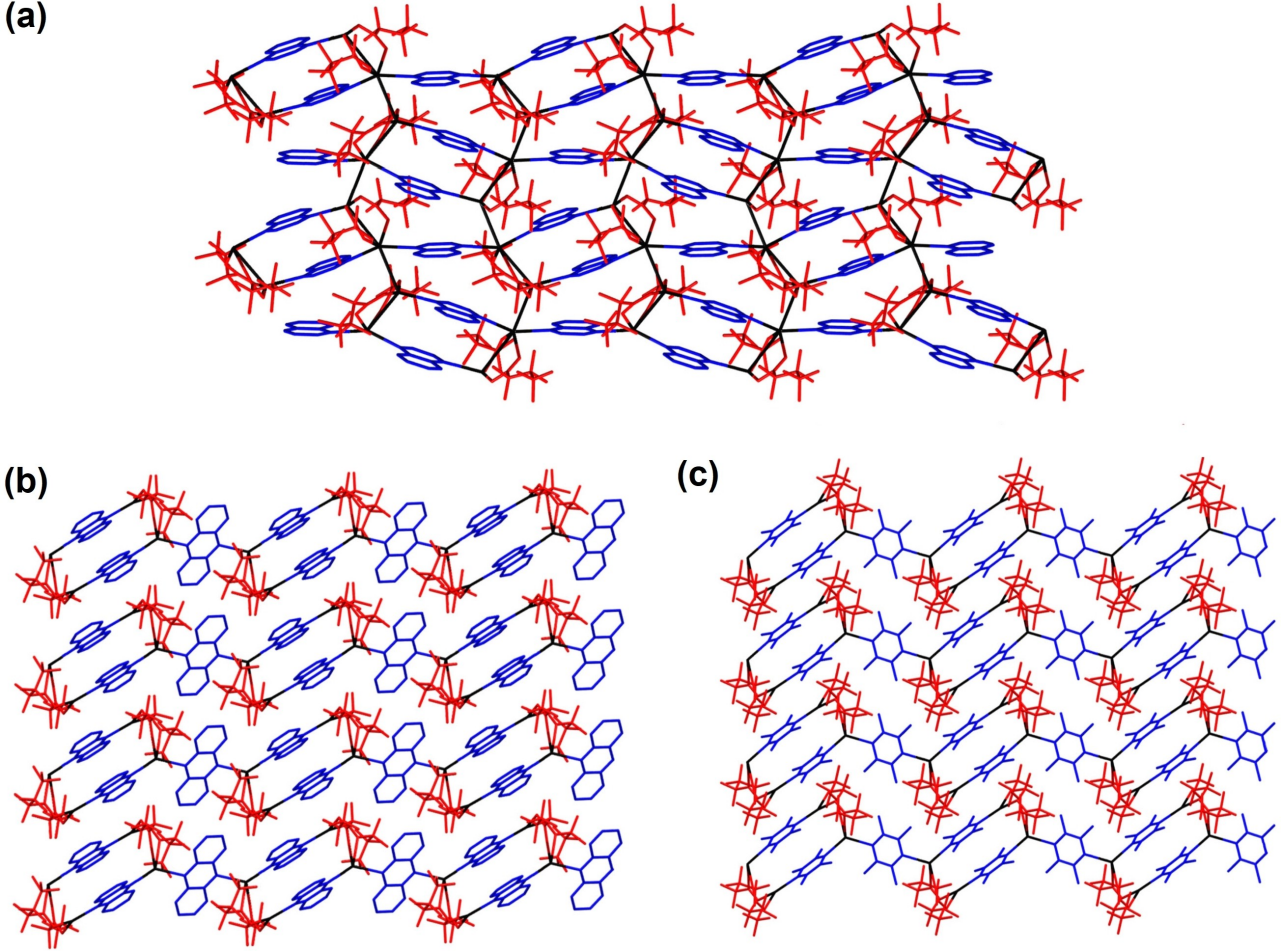
Illustration of different structures of 4 : 4 : 3 layers formed by [Ag_4_(O_2_CR_F_)_4_(diimine)_3_] coordination polymers with different diimine ligands: (a): **10**; (b): [Ag_4_(O_2_C(CF_2_)_2_CF_3_)_4_(phen)_3_];^[14]^ (c): [Ag_4_(O_2_C(CF_2_)_2_CF_3_)_4_(TMP)_3_].^[9b]^

Although analogues to the 4 : 4 : 4 phase materials have not been previously reported, analogues to the 4 : 4 : 3 phase materials with different diimine ligands are known. Figure 5 provides a comparison of **10** with two such examples, [Ag_4_(O_2_C(CF_2_)_2_CF_3_)_4_(phen)_3_] and [Ag_4_(O_2_C(CF_2_)_2_CF_3_)_4_(TMP)_3_] (phen: phenazine; TMP: tetramethylpyrazine), in which a more laterally extended (phenazine) and less extended (TMP) diimine ligand has been used. In all cases the zig‐zag tape arrangement in which the coordination polymer is propagated via alternating single and double quinoxaline bridges between Ag_2_(O_2_CR_F_)_2_ units is observed. In the materials employing phenazine and TMP ligands, however, the singly bridged diimine ligand is oriented orthogonal to the doubly bridged ones. This has the effect of keeping the zig‐zag tapes further apart and preventing assembly via argentophilic interactions within the 2D layers (Figure 5).

### Solution‐phase transformation from 4 : 4 : 3 to 4 : 4 : 4 phase

Although it was possible to isolate materials with 4 : 4 : 4 stoichiometry [Ag_4_(O_2_CR_F_)_4_(quin)_4_] or 4 : 4 : 3 stoichiometry [Ag_4_(O_2_CR_F_)_4_(quin)_3_] by adjusting the ratio of quinoxaline to silver(I) perfluorcarboxylate in the synthesis, a more extensive investigation of synthetic conditions suggests that both products can form under the same conditions, and that it is likely that the 4 : 4 : 3 phase is a kinetic product and the 4 : 4 : 4 phase the thermodynamic product. After using the same synthetic protocol that enabled synthesis of [Ag_4_(O_2_C(CF_2_)_2_CF_3_)_4_(quin)_4_] (**3**) in phase‐pure form after 31 days, the solid product was analysed by PXRD after 4 days and after 7 days. After 4 days the solid product comprised a 48.9 : 51.1 % ratio of **3 : 10** and after 7 days a 90.4 : 9.6 % ratio of **3 : 10**, established by Rietveld fitting of the PXRD patterns. Isolation of phase‐pure **3** was achieved on a shorter timescale (<3 days) by using an excess of silver(I) perfluorobutanoate in the synthesis.

Having observed the transformation from [Ag_4_(O_2_CR_F_)_4_(quin)_3_] to [Ag_4_(O_2_CR_F_)_4_(quin)_4_] in solution, the reverse transformation was explored as a solid‐state transformation, encouraged by results of thermogravimetric analysis of the 4 : 4 : 4 phase materials.

### Solid‐state transformation from 4 : 4 : 4 to 4 : 4 : 3 phase

**Figure 6:**
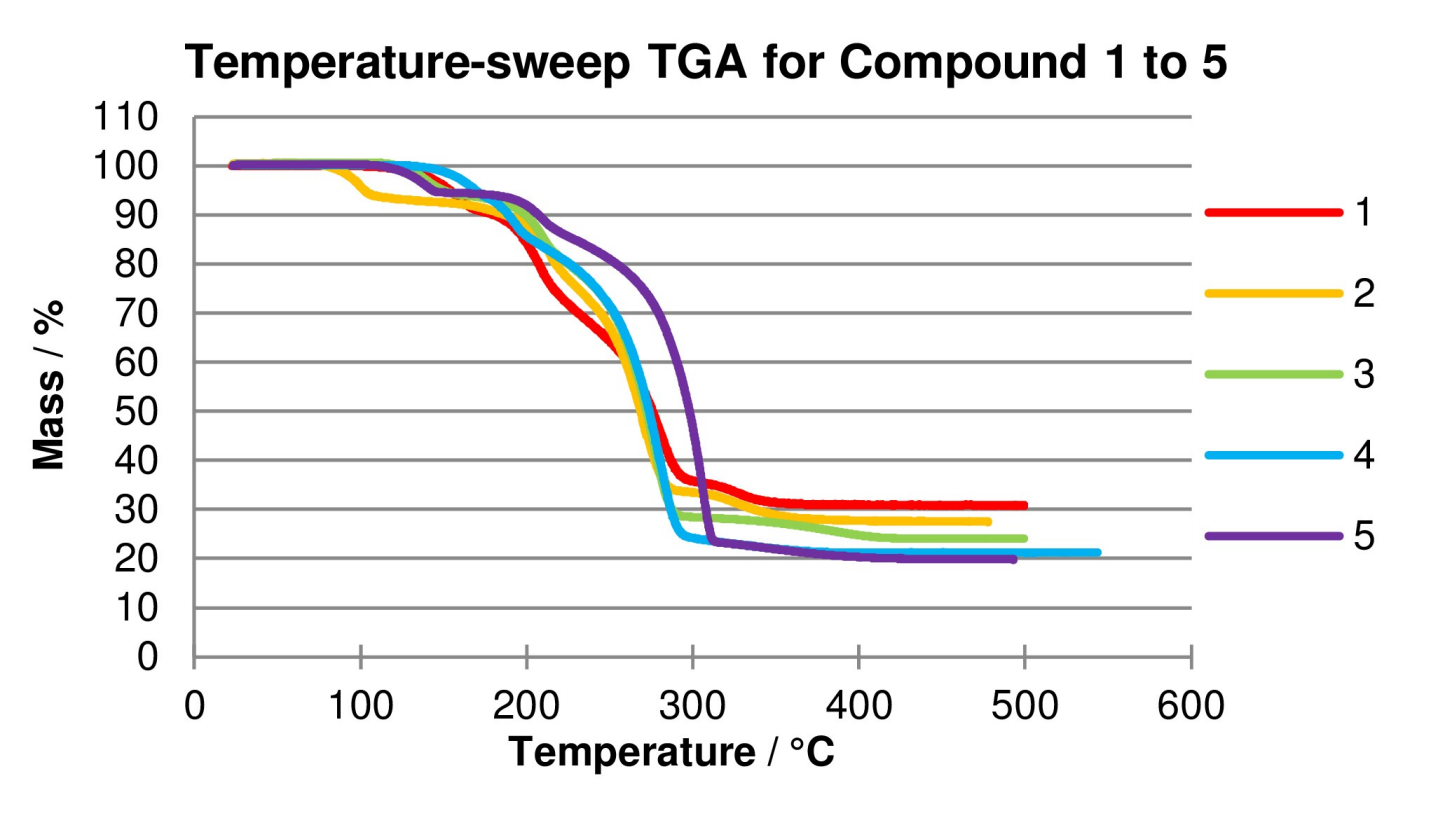
Temperature‐ramp TGA traces for 4 : 4 : 4 phase materials (**1**–**5**). Initial mass loss corresponds to loss of one quinoxaline ligand per formula unit consistent with conversion from [Ag_4_(O_2_CR_F_)_4_(quin)_4_] to [Ag_4_(O_2_CR_F_)_4_(quin)_3_]. For full experimental details and analysis, see Supporting Information.

Thermogravimetric analysis of the 4 : 4 : 4 phases **1**–**5** (Figures 6 and S11–S22) show mass loss patterns on heating, indicative of an initial loss of one quinoxaline ligand per [Ag_4_(O_2_CR_F_)_4_(quin)_4_] formula unit with an onset temperature in the range 85–125 °C, followed by secondary mass loss of 1–2 quinoxaline ligands, before final mass losses consistent with decomposition of the perfluorocarboxylate ligands. The stability of the material after loss of one quinoxaline ligand to form a material with composition [Ag_4_(O_2_CR_F_)_4_(quin)_3_] is further confirmed by isothermal TGA traces at selected temperatures close to the onset temperature of the initial mass loss (Figures S12 for **1**, S14 for **2**, S16 for **3**, S18‐S20 for **4** and S22 for **5**).

Encouraged by the TGA results, and aware that solid‐state transformations in related families of materials have been observed,[^9^, ^10^] we undertook in situ diffraction studies to investigate the ligand‐loss reaction [Ag_4_(O_2_CR_F_)_4_(quin)_4_] to [Ag_4_(O_2_CR_F_)_4_(quin)_3_] for compounds **1** and **3**, for which the structures of the starting 4 : 4 : 4 phase and the product 4 : 4 : 3 phase (**8** and **10**) had been established crystallographically. We similarly investigated the heating of [Ag_4_(O_2_C(CF_2_)_4_CF_3_)_4_(quin)_4_] **5**, for which TGA results suggest formation of [Ag_4_(O_2_C(CF_2_)_4_CF_3_)_4_(quin)_3_] by loss of one equivalent of quinoxaline, but for which independent direct solution‐phase synthesis was unsuccessful. Single‐crystal‐to‐single‐crystal transformations from 4 : 4 : 4 phases to 4 : 4 : 3 phases by heating were not successful as single‐crystal crystallinity was not retained. In situ PXRD studies, however, were more informative.

**Figure 7:**
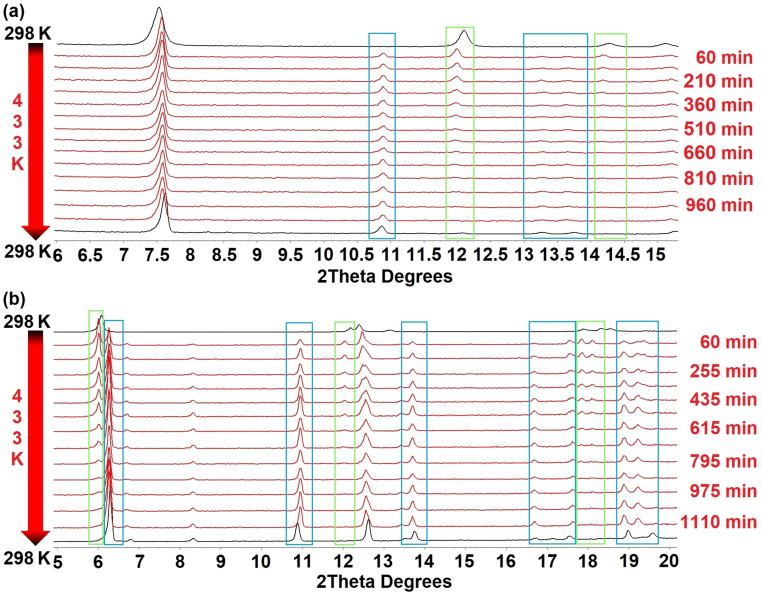
(a) In situ PXRD patterns showing the transformation of **1** to **8**, shown in the range 6≤2θ≤15.5° (b) In situ PXRD patterns showing the transformation of **3** to **10**, shown in the range 5≤2θ≤20°. Increasing or decreasing intensity of reflections during the phase change are highlighted with blue boxes and green boxes, respectively.

**Figure 8:**
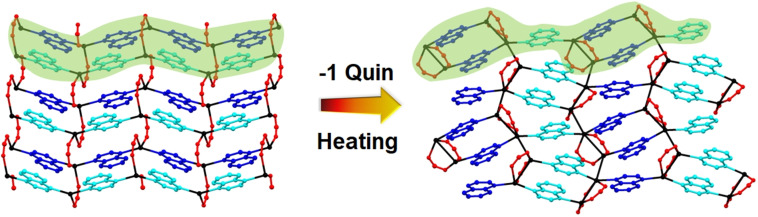
Solid‐state reaction involves loss one quinoxaline ligand per formula unit leading to conversion of [Ag_4_(O_2_CR_F_)_4_(quin)_4_] (4 : 4 : 4 phase) to [Ag_4_(O_2_CR_F_)_4_(quin)_3_] (4 : 4 : 3 phase). Hydrogen atoms and perfluoroalkyl chains are not shown. Ag (black); carboxylates (O_2_C, red); quinoxaline ligand orientation: ligand points into the page (blue); ligand points out of the page (cyan). Green shading shows individual [Ag_4_(O_2_CR_F_)_4_(quin)_4_] and [Ag_4_(O_2_CR_F_)_4_(quin)_3_] tapes that combine to make the 2D networks in these materials.

In each study, after an initial powder pattern obtained at 298 K, the capillary containing the sample was heated to 433 K and a pattern measured every 75–90 mins for a total of 18–19 h before a final measurement was made at 298 K. All patterns were analysed using Pawley fitting to identify whether a single or multiple phases were present and to determine unit cell dimensions of these phases (Figures S23–S60). The isothermal heating studies show a gradual transformation from 4 : 4 : 4 phase to 4 : 4 : 3 phase for **1**→**8** and **3**→**10** (Figure 7) and clearly demonstrate that the solid‐state reaction involving extrusion of quinoxaline upon heating and the associated rearrangement takes place with retention of crystallinity. The solid‐state reaction to convert [Ag_4_(O_2_CR_F_)_4_(quin)_4_] (4 : 4 : 4 phase) to [Ag_4_(O_2_CR_F_)_4_(quin)_3_] (4 : 4 : 3 phase) involves breaking of both Ag−N and Ag−O coordination bonds and formation of new bonds of both types and requires migration of carboxylate ligands and reorientation of quinoxaline ligands (Figure 8).

**Scheme 1:**
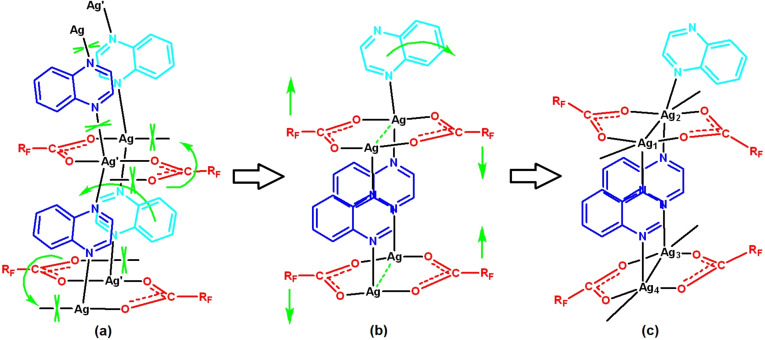
Proposed transformation from [Ag_4_(O_2_CR_F_)_4_(quin)_4_] (*4 : 4 : 4 phase*) to [Ag_4_(O_2_CR_F_)_4_(quin)_3_] (*4 : 4 : 3 phase*), illustrating in (a)→(b) loss of quinoxaline and migration of carboxylate ligands and in (b) to (c) the resultant reorientation of the perfluoroalkyl groups (R_F_) that point into the interlayer regions.

This reaction and rearrangement process is envisaged as shown schematically in Scheme 1.

### Thermal expansion studies and further mechanistic insight

**Figure 9:**
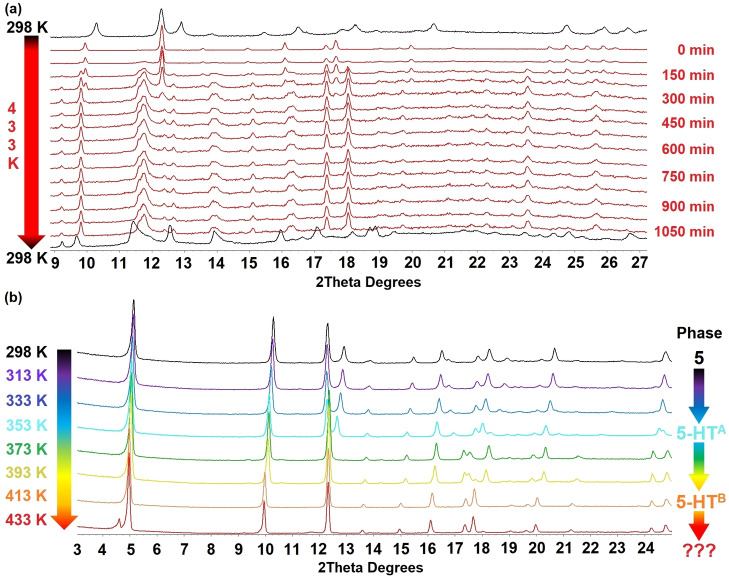
(a) In situ PXRD patterns showing the transformation of **5** to **5‐HT^B^** and a subsequent unidentified phase upon direct heating at 433 K; patterns shown in the range 9≤2θ≤27° (b) In situ PXRD patterns showing the transformation of **5** to **5‐HT^A^** to **5‐HT^B^** and a subsequent unidentified phase during gradual increase in temperature; patterns shown in the range 3≤2θ≤25°.

For **5**, which has the longest perfluoroalkylcarboxylate of the compounds studied, heating initially leads to formation a new single phase of slightly larger unit cell volume (Δ*V*=6.5 %), which we will refer to as **5‐HT^B^**. Heating at 433 K for over 6 h leads to a mixed‐phase pattern involving **5‐HT^B^** and a new phase, the peaks for which could not be indexed. Transformation to an indexable pattern did not occur within the 18 h period of the heating study (Figure 9a).^[15]^ A further heating study in which PXRD patterns were measured at 10–20 K increments during heating from 298 K to 433 K showed that **5** is first transformed into a new single phase (**5‐HT^A^**) in the temperature range 353–373 K (Figure 9b). Further heating leads to transformation of **5‐HT^A^** into **5‐HT^B^** in the temperature step from 393 K to 413 K, after which new peaks evolve at 433 K, indicating a further transformation is beginning.

**Figure 10:**
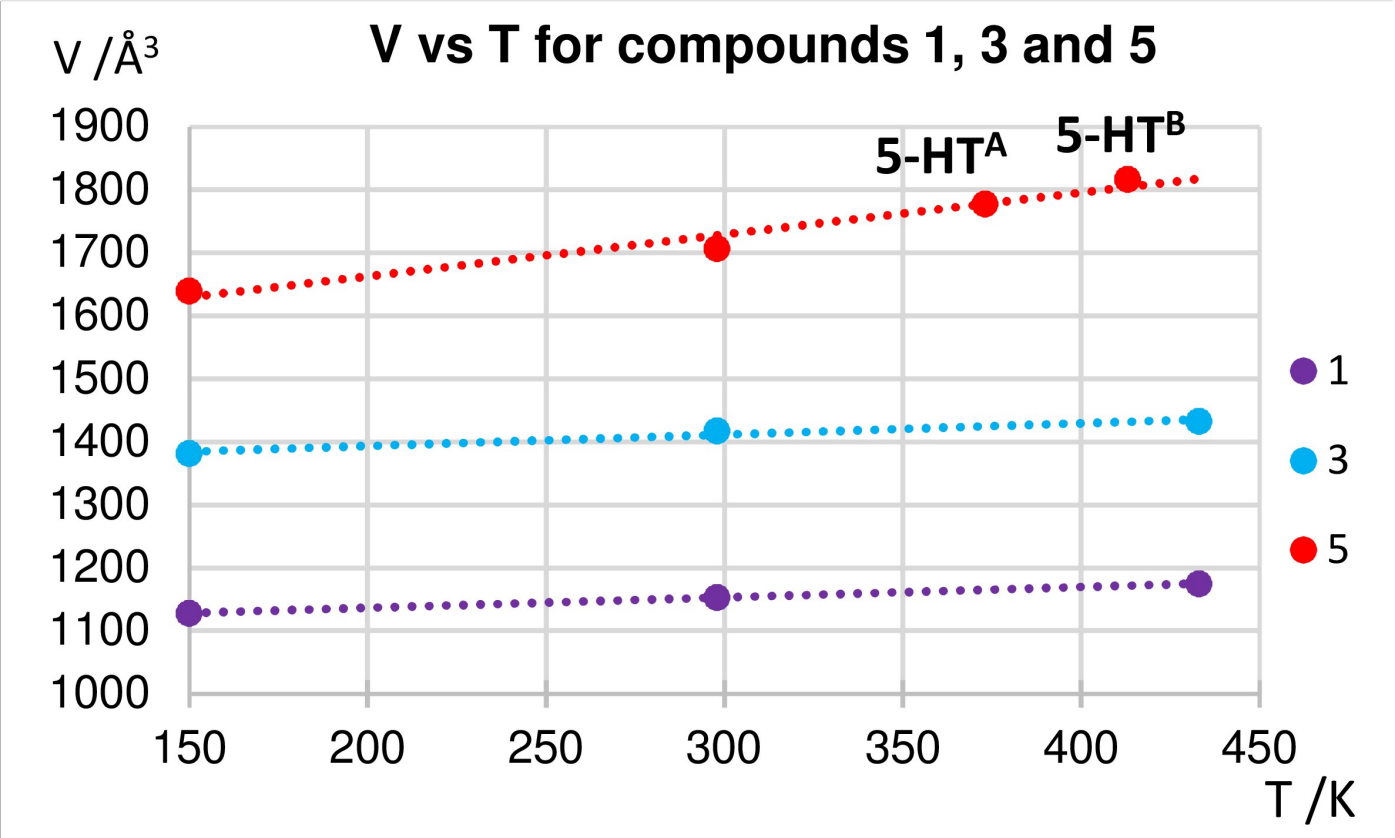
Linear expansion of unit cell volume with temperature for **1**, **3** and **5**. Values at 150 K from SCXRD data, otherwise from Pawley fits of PXRD data.

Determination of unit cell dimensions for **1**, **3** and **5** as a function of temperature in the range 150–433 K reveals a linear expansion in unit cell volume (Figure 10). In the case of **1** and **3** there is no change of phase, and prolonged heating at 433 K then leads to quinoxaline ligand loss (see above). For **5**, we interpret the behaviour to indicate that thermal expansion is associated with phase changes (from **5** to **5‐HT^A^** then to **5‐HT^B^**, accompanied by a change in lattice symmetry, Table S9), which involves an expansion of the interlayer spacing and is likely to involve conformational changes in the interlayer perfluoroalkyl chains in the high‐temperature (HT) phases, as seen previously in related materials.^[9c]^ The studies of thermal expansion suggest that, upon heating, all the [Ag_4_(O_2_CR_F_)_4_(quin)_4_] (4 : 4 : 4 phase) materials undergo thermal expansion, notably in the interlayer region (expansion of *a*‐axis), which may facilitate release of quinoxaline in the conversion to [Ag_4_(O_2_CR_F_)_4_(quin)_3_] (4 : 4 : 3 phase). In the case of **5**, the interlayer region contains more extensive interdigitation due to the longer perfluoroalkyl chains, leading to distinct phase changes on heating, as the material undergoes greater expansion than its shorter chain analogues (Figure 10) and has greater potential for conformational changes, before the release of quinoxaline.^[15]^

### Dielectric constant measurements

**Figure 11:**
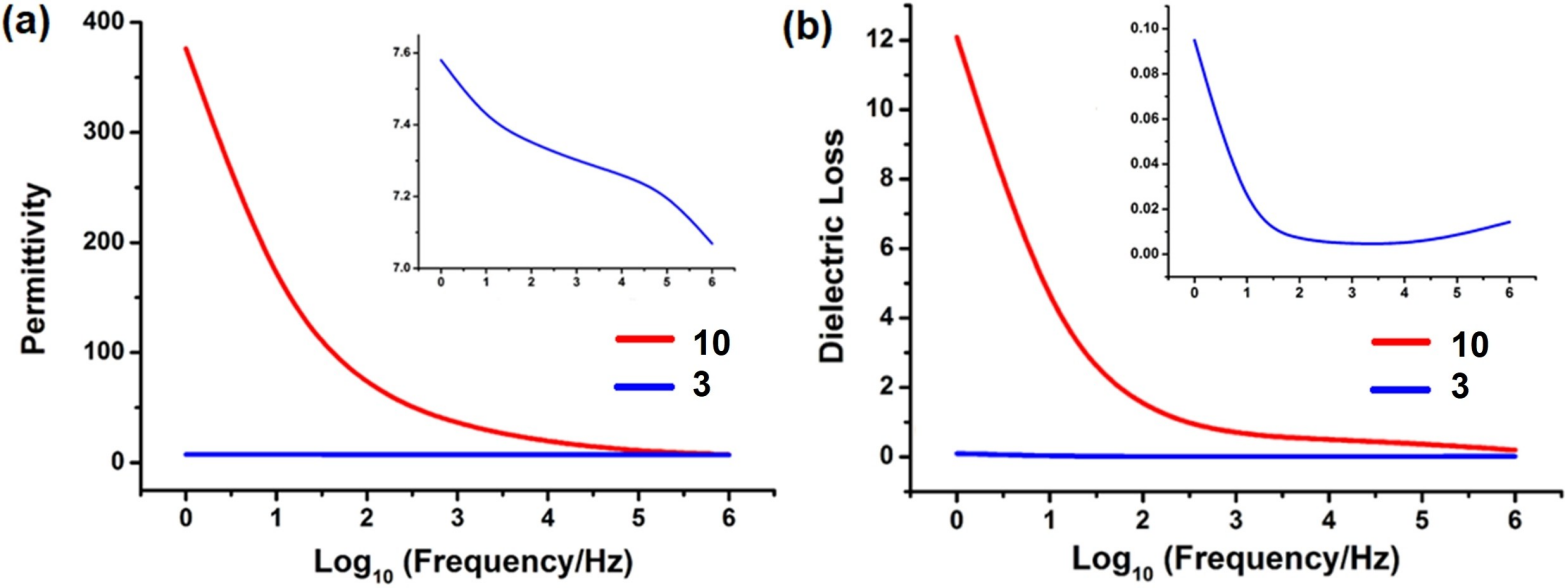
(a) Frequency‐dependent dielectric permittivity (ϵ_r_) of **3** and **10** at room temperature. (b) Frequency‐dependent dielectric loss (DL) for **3** and **10** at room temperature. Insets: expanded plots for **3**.

High dielectric constant materials (high‐κ materials) have received much interest due to their potential applications in modern electronic and electrical power systems such as high energy density capacitors, resonators, filters, and other key components in microwave communication systems.^[16]^ To date, most of these high‐κ materials are inorganic ceramics, organic polymers, organic/inorganic hybrids and polymer nanocomposites.^[17]^ and examples of coordination compounds remain quite limited.^[18]^ We have therefore taken the opportunity to test the dielectric performance of a pair of compounds, **3** and **10**, as an indication of the effect on this property of the solid‐state transformation that has been investigated structurally and mechanistically (see above). The two compounds exhibit markedly different frequency dependent behaviour at room temperature (Figure 11).

The 4 : 4 : 4 phase (**3**) exhibits a modest dielectric constant (ϵ_r_ 7.58–7.07), with little change over the frequency range 1 Hz–1 MHz. The corresponding 4 : 4 : 3 phase (**10**), by contrast, exhibits a remarkably high ϵ_r_ (376.1) at 1 Hz which drops quickly upon increasing the frequency (7.29 at 1 MHz), and suggests that it may be suitable to function as a high‐κ material. The dielectric loss (*DL*, which is denoted as the energy loss within dielectric materials), for **10** drops quickly from 12.08 to 1.24 below 100 Hz and then decreases more slowly. By contrast, the *DL* of **3** decreases first and then slightly rises with increasing frequency, exhibiting a nearly frequency‐independent behaviour with a magnitude of only approximately 10^−3^ in the range of 10^2^–10^5^ Hz. The results suggest that **10** may be a good candidate for further investigation as a high‐κ material at low frequency at room temperature, and that other 4 : 4 : 3 phase materials in this family may be warrant follow‐up measurements.

## Conclusions

We report the syntheses and structures of ten 2D coordination polymers formed from combination of silver perfluorocarboxylates (AgO_2_CR_F_) and quinoxaline (quin), which are classified as two structure types, dependent on their stoichiometry: [Ag_4_(O_2_CR_**F**_)_4_(quin)_4_] (denoted as 4 : 4 : 4 phases, **1**–**7**) and [Ag_4_(O_2_CR_**F**_)_4_(quin)_3_] (denoted as 4 : 4 : 3 phases, **8**–**10**). We have established from the solution‐phase syntheses that the 4 : 4 : 3 phase is the kinetic product and the 4 : 4 : 4 phase is the thermodynamic product, but synthesis can be controlled to yield exclusively one product in most cases. More remarkably, we have shown that crystalline 4 : 4 : 4 phases can be converted to the corresponding 4 : 4 : 3 phase by heating in a solid‐state transformation that involves loss of 25 % of quinoxaline ligands and substantial rearrangement of the structure involving breaking and formation of multiple metal–ligand bonds, ligand migrations and reorientations. This transformation, involving retention of crystallinity, has been followed in situ by PXRD. More detailed studies of thermal expansion of the 4 : 4 : 4 phases shows that the likely mechanism involves expansion of the interlayer spacing and, where longer perfluoroalkyl carboxylates are used, mobility of the perfluoroalkyl chains is likely to be involved in the extrusion of the quinoxaline ligands, consistent with behaviour established for related materials.^[9c]^ Finally, we note that the 4 : 4 : 3 phase [Ag_4_(O_2_C(CF_2_)_2_CF_3_)_4_(quin)_3_] (**10**) exhibits a remarkably high dielectric constant, in contrast to its 4 : 4 : 4 phase analogue (**3**), suggesting that further exploration as a potential as a high‐κ material at low frequency is warranted.

## Experimental Section

**General**: All the reactants were purchased from Sigma Aldrich, Alfa Aesar or Fluorochem and used as received. Elemental analyses were carried out on a Perkin‐Elmer 2400 CHNS/O Series II elemental analyzer in the Department of Chemistry. Thermogravimetric analyses were recorded on a Perkin‐Elmer Pyris1 TGA model thermogravimetric analyser under a flow of dry N_2_ gas. Dielectric constants and losses (frequency range 1–10^6^ Hz) were measured on a broadband dielectric spectrometer (Novocontrol Concept 80, Germany).

### Syntheses

**Preparation of [Ag_4_(O_2_CCF_3_)_4_(quin)_4_] (1)**: Quinoxaline (6.5 mg, 0.05 mmol) was dissolved in ethyl acetate (1.0 mL) in a 20 mL glass vial and gently layered with ethyl acetate (2 mL) and then acetone (2 mL) onto this solution. Then an acetone solution (1.0 mL) of silver(I) trifluoroacetate (10 mg, 0.045 mmol) was carefully layered onto the buffer layer. The vial was tightly capped and stored in a dark cupboard to minimize the possibility of light degradation of silver ions. Yellow crystals of **1** were formed within one week. Yield: 14.6 mg (93 %). Anal. Calcd. for C_40_H_24_Ag_4_F_12_N_8_O_8_: C 34.22; H 1.72; N 7.98; found: C 34.01; H 1.67; N 7.82 %.

**Preparation of [Ag_4_(O_2_CCF_2_CF_3_)_4_(quin)_4_] (2)**: An analogous layering synthesis to that used for **1** was applied for preparing **2**, using a methanol solution (1.0 mL) of quinoxaline (32 mg, 0.246 mmol), a methanol solution (1.0 mL) of silver(I) pentafluoropropanoate (30 mg, 0.11 mmol) and without applying a buffer layer. Yellow crystals of **2** were generated within 3 days. Yield: 9.5 mg (21 %). Anal. Calc. for C_44_H_24_Ag_4_F_20_N_8_O_8_: C 32.92; H 1.50; N 6.98; found: C 32.72; H 1.78; N 6.75 %.

**Preparation of [Ag_4_(O_2_C(CF_2_)_2_CF_3_)_4_(quin)_4_] (3)**: An analogous layering synthesis to that used for **1** was applied for preparing **3**, using an ethyl acetate solution (1.0 mL) of quinoxaline (13 mg, 0.10 mmol), a buffer solution of ethyl acetate (2 mL) and acetone (2 mL) and an acetone solution (1.0 mL) of silver(I) heptafluorobutanoate (29 mg, 0.09 mmol). Pale yellow crystals of **3** were formed within 14 days. Phase purity was established by PXRD and elemental analysis after 31 days. Yield: 18.7 mg (46 %). Anal. Calcd. for C_44_H_24_Ag_4_F_20_N_8_O_8_: C, 31.95; H, 1.33; N, 6.21 %; found C, 31.87; H, 1.20; N, 6.31 %.

**Preparation of [Ag_4_(O_2_C(CF_2_)_3_CF_3_)_4_(quin)_4_] (4)**: An analogous layering synthesis to that used for **1** was applied for preparing **4**, using an ethyl acetate solution (1.0 mL) of quinoxaline (13 mg, 0.10 mmol), a buffer solution of ethyl acetate (2 mL) and acetone (2 mL) and an acetone solution (1.0 mL) of silver(I) nonafluoropentanoate (16.5 mg, 0.045 mmol). Pale yellow crystals of **4** were afforded within 4 days. Yield: 11 mg (48 %). Anal. Calcd. for C_52_H_24_Ag_4_F_36_N_8_O_8_: C 31.16; H 1.21; N 5.59; found: C 31.24; H 1.43; N 5.52 %.

**Preparation of [Ag_4_(O_2_C(CF_2_)_4_CF_3_)_4_(quin)_4_] (5)**: An analogous layering synthesis to that used for **1** was applied for preparing **5**, using an ethyl acetate solution (4.0 mL) of quinoxaline (64 mg, 0.49 mmol), a buffer solution of ethyl acetate (0.5 mL) and acetone (0.5 mL) and an acetone solution (4.0 mL) of silver(I) undecafluorohexanoate (151.2 mg, 0.36 mmol). Yellow crystals were produced after 1 day and gradually turned into yellow crystals of **5** within 2 days. Yield: 126 mg (64 %). Anal. Calcd. for C_56_H_24_Ag_4_F_44_N_8_O_8_: C 30.52; H 1.10; N 5.08; found: C 30.60; H 1.46; N 5.17 %.

**Preparation of [Ag_4_((O_2_C)_2_C_4_F_8_)_4_(quin)_4_] (6)**: Ag_2_O (17.4 mg, 0.075 mmol) and octafluoroadipic acid (C_4_F_8_(CO_2_H)_2_) (21.7 mg, 0.075 mmol) were combined in 30 % aqueous NH_3_ (4 mL) and added to quinoxaline (9.6 mg, 0.075 mmol) in 30 % aqueous NH_3_ (4 mL). Yellow crystals of **6** (50.8 mg, yield 26 %) resulted after 35 days of slow evaporation of solvent in the absence of light. Analysis calc.: C, 33.88, H, 1.24, N, 6.80. Found: C, 31.96, H, 1.15, N, 5.35.

**Preparation of [Ag_4_(O_2_C(C_6_F_5_))_4_(quin)_4_] (7)**: Ag_2_O (17.4 mg, 0.075 mmol) and C_6_F_5_CO_2_H (33.8 mg, 0.075 mmol) were combined in 30 % aqueous NH_3_ (4 mL) and added to quinoxaline (9.6 mg, 0.075 mmol) in 30 % aqueous NH_3_ (4 mL). Yellow crystals of **7** (75.2 mg, yield 92 %) resulted after 40 days of slow evaporation of solvent in the absence of light. Analysis calc.: C, 40.18, H, 1.35, N, 6.25. Found: C, 39.87, H, 1.28, N, 6.18.

**Preparation of [Ag_4_(O_2_CCF_3_)_4_(quin)_3_] (8)**: An analogous layering synthesis to that used for **1** was applied for preparing **8**, using an ethyl acetate solution (1.0 mL) of quinoxaline (6.5 mg, 0.05 mmol), a buffer solution of ethyl acetate (2 mL) and acetone (2 mL) and an acetone solution (1.0 mL) of silver(I) trifluoroacetate (20 mg, 0.09 mmol). Pale yellow crystals of **8** were formed within 2 days. Yield: 6.5 mg (60 %). Anal. Calcd. for C_32_H_18_Ag_4_F_12_N_6_O_8_: C 30.15; H 1.41; N 6.60; found: C 30.14; H 1.70; N 6.56 %.

**Preparation of [Ag_4_(O_2_CCF_2_CF_3_)_4_(quin)_3_] (9)**: An analogous layering synthesis to that used for **1** was applied for preparing **9**, using an ethyl acetate solution (1.0 mL) of quinoxaline (6.0 mg, 0.046 mmol), a buffer solution of ethyl acetate (2 mL) and acetone (2 mL) and an acetone solution (1.0 mL) of silver(I) pentafluoropropanoate (16.8 mg, 0.062 mmol). Off‐white crystals of **9** were formed within 3 days. Yield: 5.1 mg (23 %). Anal. Calcd. for C_36_H_18_Ag_4_F_20_N_6_O_8_: C 29.35; H 1.22; N 5.70; found: C 29.29; H 1.12; N 5.62 %.

**Preparation of [Ag_4_(O_2_C(CF_2_)_2_CF_3_)_4_(quin)_3_] (10)**: An analogous layering synthesis to that used for **1** was applied for preparing **10**, using an ethyl acetate solution (2.0 mL) of quinoxaline (13.0 mg, 0.10 mmol), a buffer solution of ethyl acetate (2 mL) and acetone (2 mL) and an acetone solution (2.0 mL) of silver(I) heptafluorobutanoate (58.0 mg, 0.18 mmol). Off‐white crystals of **10** were formed within 3 days. Yield: 30 mg (58 %). Anal. Calcd. for C_40_H_18_Ag_4_F_28_N_6_O_8_: C 28.68; H 1.08; N 5.02; found: C 28.92; H 1.10; N 5.06 %.

**Investigation of time‐evolution of syntheses of (3) and (10)**: An analogous layering synthesis to that used for **1** was used to explore the time‐evolution of the synthesis of **3** and **10** to establish if interconversion takes places between the two over time. An ethyl acetate solution (2.0 mL) of quinoxaline (13.0 mg, 0.10 mmol), a buffer solution of ethyl acetate (2 mL) and acetone (2 mL) and an acetone solution (2.0 mL) of silver(I) heptafluorobutanoate (29.0 mg, 0.09 mmol) was allowed to react for 4 days before analysis of the solid product by PXRD showed a 48.9 : 51.1 % ratio of **3 : 10**, established by Rietveld fitting. A separate reaction set up under identical conditions was allowed to react for 7 days, whereupon analysis of the solid product by PXRD showed a 90.4 : 9.6 % ratio of **3 : 10**, established by Rietveld fitting.

**Single‐crystal X‐ray diffraction**: Single‐crystal X‐ray data were collected on a Bruker D8 *VENTURE* diffractometer, equipped with a PHOTON 100 CMOS detector (Cu‐K_α_ radiation) or a Bruker APEX‐II diffractometer (Mo‐K_α_ radiation). Crystal structures were solved with direct methods or Patterson methods using the *SHELXTL* ^[19]^ or *Olex2* ^[20]^ programs. All structures were refined against all *F* ^2^ values and a multi‐scan method (*SADABS*)^[21]^ was used for absorption correction. Non‐H atoms were refined with anisotropic displacement parameters. Hydrogen atoms were added at calculated positions and refined with a riding model and isotropic displacement parameters fixed in magnitude relative to the attached carbon atoms. Disordered parts of some structures were modelled with reasonable occupancies and isotropic displacement parameters. Details of crystal data, structure solution and refinement parameters can be found in Table S1.

**Powder X‐ray diffraction**: Powder X‐ray diffraction data for phase purity checks were recorded at room temperature either at University of Sheffield (compounds **5**, **8**, **9** and **10**) or Diamond Light Source Synchrotron (compounds **1**, **2**, **3** and **4**). The Bruker D8 ADVANCE diffractometer at University of Sheffield was fitted with a focusing Göbel mirror optic and a high‐resolution energy‐dispersive Lynxeye XE detector and operated in a capillary mode (Debye‐Scherrer) or flat‐plate mode (Bragg‐Brentano) using Cu−Kα radiation. For capillary mode, the sample was packed in a 0.7 mm borosilicate capillary, whereas in flat‐plate mode each sample was loaded on a 14 mm silicon zero‐background sample disc. Samples were rotated at 30 rot/min to average sample exposure (4 sec step^−1^ and 0.02° step size). Beamline I11 at Diamond Light Source^[22]^ was equipped with a wide angle (90°) PSD detector comprising 18 Mythen‐2 modules (*λ*=0.82562 Å). The samples were packed into 0.7 mm borosilicate capillaries and five pairs of scans were collected for 10 s per pattern (2×10 s per pair), as well as one 1 s scan at both the start and the end of each pair to check for radiation damage. These patterns were summed to give the final pattern. All patterns were indexed and fitted by Pawley refinement^[23]^ using the *TOPAS‐Academic* program.^[24]^

**PXRD studies of time‐evolution of synthesis of 3 and 10**: PXRD data were collected for product of the reaction of quinoxaline with silver(I) heptafluorobutanoate to monitor the production of (**3**) and (**10**) as function of reaction time after 4 days and after 7 days. All the patterns were collected at room temperature and diffraction patterns were fitted using the *TOPAS‐Academic* program. For the product after 4 days’ reaction PXRD data the powder was loaded into a 0.5 mm borosilicate capillary. X‐ray diffraction data were collected using Cu−Kα radiation at University of Sheffield, Department of Materials Science on a STOE STADI P diffractometer equipped with a PSD detector. A single scan (5≤2θ≤40 °) was measured at a scan rate of 0.067 ° min^−1^, using a rotating capillary. For the product after 7 days’ reaction PXRD data the white microcrystalline powder was loaded into a 0.7 mm quartz capillary. X‐ray diffraction data were collected (*λ*=0.82665 Å) at beamline I11 at Diamond Light Source, as described above.

**In situ PXRD heating studies of loss of quinoxaline from coordination polymers**: In situ PXRD heating studies were recorded on a Bruker D8 ADVANCE X‐ray powder diffractometer. Samples were loaded in a 0.7 mm borosilicate capillary which was open at both ends. Data were collected as described above. The patterns were indexed and fitted using Pawley and Rietveld^[25]^ refinement using the *TOPAS Academic* program.

## Conflict of interest

The authors declare no conflicts of interest.

1

## Supporting information

As a service to our authors and readers, this journal provides supporting information supplied by the authors. Such materials are peer reviewed and may be re‐organized for online delivery, but are not copy‐edited or typeset. Technical support issues arising from supporting information (other than missing files) should be addressed to the authors.

Supporting Information

## Data Availability

The data that support the findings of this study are available in the Supporting Information of this article. Deposition Number(s) 2170223–2170232 (for 1–10; see Table S1) and 2170855 for [Ag_4_(O_2_C(CF_2_)_3_CF_3_)_4_(phen)_3_] contain(s) the supplementary crystallographic data for this paper. These data are provided free of charge by the joint Cambridge Crystallographic Data Centre and Fachinformationszentrum Karlsruhe Access Structures service.
