# Supplementary material for: Metal–ligand Lability and Ligand Mobility Enables Framework Transformation via Ligand Release in a Family of Crystalline 2D Coordination Polymers
Source: Chemistry. 2022 Jul 19;28(51):e202201408. doi: 10.1002/chem.202201408 (PMC9543667; doi:10.1002/chem.202201408)
Supplement: Supplementary file 1 — Supporting Information [file CHEM-28-0-s001.pdf]

Chemistry–A European Journal

Supporting Information

Metal–ligand Lability and Ligand Mobility Enables Framework Transformation via Ligand Release in a Family of Crystalline 2D Coordination Polymers

Feifan Lang, Daljit C. N. G. Singh, Abhishek B. Rao, Catherine Romer, James S. Wright, Rebecca Smith, Harry Adams, and Lee Brammer*

Table of Contents

S1. Synthesis of coordination polymers **Page S2**

S2. Single-crystal X-ray diffraction **Page S3**

S3. Phase purity checks by powder X-ray diffraction (PXRD) **Page S7**

S4. PXRD studies of time-evolution of synthesis of 3 and 10 **Page S11**

S5. Thermogravimetric analysis **Page S13**

S6. *In situ* PXRD heating studies of loss of quinoxaline from 1, 3 and 5 **Page S19**

S7. Diffraction studies of thermal expansion and phase change for 1, 3 and 5 **Page S43**

S8. Dielectric constant test **Page S45**

S9. CIF Check Alert A and B responses **Page S45**

S10. References **Page S46**

Open Access statement **Page S46**

S1. Synthesis of coordination polymers

Preparation of [$Ag_4(O_2CCF_3)_4(quin)_4$] (1). Quinoxaline (6.5 mg, 0.05 mmol) was dissolved in ethyl acetate (1.0 mL) in a 20 mL glass vial and gently layered with ethyl acetate (2 mL) and then acetone (2 mL) onto this solution. Then an acetone solution (1.0 mL) of silver(I) trifluoroacetate (10 mg, 0.045 mmol) was carefully layered onto the buffer layer. The vial was tightly capped and stored in a dark cupboard to minimize the possibility of light degradation of silver ions. Yellow crystals of **1** were formed within one week. Yield: 14.6 mg (93%). Anal. Calcd. for $C_{40}H_{24}Ag_4F_{12}N_8O_8$: C 34.22; H 1.72; N 7.98; found: C 34.01; H 1.67; N 7.82%.

Preparation of [$Ag_4(O_2CCF_2CF_3)_4(quin)_4$] (2). An analogous layering synthesis to that used for **1** was applied for preparing **2**, using a methanol solution (1.0 mL) of quinoxaline (32 mg, 0.246 mmol), a methanol solution (1.0 mL) of silver(I) pentafluoropropanoate (30 mg, 0.11 mmol) and without applying a buffer layer. Yellow crystals of **2** were generated within 3 days. Yield: 9.5 mg (21%). Anal. Calc. for $C_{44}H_{24}Ag_4F_{20}N_8O_8$: C 32.92; H 1.50; N 6.98; found: C 32.72; H 1.78; N 6.75%.

Preparation of [$Ag_4(O_2C(CF_2)_2CF_3)_4(quin)_4$] (3). An analogous layering synthesis to that used for **1** was applied for preparing **3**, using an ethyl acetate solution (1.0 mL) of quinoxaline (13 mg, 0.10 mmol), a buffer solution of ethyl acetate (2 mL) and acetone (2 mL) and an acetone solution (1.0 mL) of silver(I) heptafluorobutanoate (29 mg, 0.09 mmol). Pale yellow crystals of **3** were formed within 14 days. Phase purity was established by PXRD and elemental analysis after 31 days. Yield: 18.7 mg (46%). Anal. Calcd. for $C_{44}H_{24}Ag_4F_{20}N_8O_8$: C, 31.95; H, 1.33; N, 6.21%; found C, 31.87; H, 1.20; N, 6.31%.

Preparation of [$Ag_4(O_2C(CF_2)_3CF_3)_4(quin)_4$] (4). An analogous layering synthesis to that used for **1** was applied for preparing **4**, using an ethyl acetate solution (1.0 mL) of quinoxaline (13 mg, 0.10 mmol), a buffer solution of ethyl acetate (2 mL) and acetone (2 mL) and an acetone solution (1.0 mL) of silver(I) nonafluoropentanoate (16.5 mg, 0.045 mmol). Pale yellow crystals of **4** were afforded within 4 days. Yield: 11 mg (48%). Anal. Calcd. for $C_{52}H_{24}Ag_4F_{36}N_8O_8$: C 31.16; H 1.21; N 5.59; found: C 31.24; H 1.43; N 5.52%.

Preparation of [$Ag_4(O_2C(CF_2)_4CF_3)_4(quin)_4$] (5). An analogous layering synthesis to that used for **1** was applied for preparing **5**, using an ethyl acetate solution (4.0 mL) of quinoxaline (64 mg, 0.49 mmol), a buffer solution of ethyl acetate (0.5 mL) and acetone (0.5 mL) and an acetone solution (4.0 mL) of silver(I) undecafluorohexanoate (151.2 mg, 0.36 mmol). Yellow crystals were produced after 1 day and gradually turned into yellow crystals of **5** within 2 days. Yield: 126 mg (64%). Anal. Calcd. for $C_{56}H_{24}Ag_4F_{44}N_8O_8$: C 30.52; H 1.10; N 5.08; found: C 30.60; H 1.46; N 5.17%.

Preparation of [$Ag_4((O_2C)_2C_4F_8)_4(quin)_4$] (6). Ag_2O (17.4mg, 0.075mmol) and octafluoroadipic acid ($C_4F_8(CO_2H)_2$) (21.7mg, 0.075mmol) were combined in 30% aqueous NH_3 (4 mL) and added to quinoxaline (9.6mg, 0.075 mmol) in 30% aqueous NH_3 (4 mL). Yellow crystals of **6** (50.8mg, yield 26%) resulted after 35 days of slow evaporation of solvent in the absence of light. Analysis calc.: C, 33.88, H, 1.24, N, 6.80. Found: C, 31.96, H, 1.15, N, 5.35.

Preparation of [$Ag_4(O_2C(C_6F_5))_4(quin)_4$] (7). Ag_2O (17.4mg, 0.075mmol) and $C_6F_5CO_2H$ (33.8mg, 0.075mmol) were combined in 30% aqueous NH_3 (4 mL) and added to quinoxaline (9.6mg, 0.075 mmol) in 30% aqueous NH_3 (4 mL). Yellow crystals of **7** (75.2mg, yield 92%) resulted after 40 days

of slow evaporation of solvent in the absence of light. Analysis calc.: C, 40.18, H, 1.35, N, 6.25. Found: C, 39.87, H, 1.28, N, 6.18.

Preparation of [$Ag_4(O_2CCF_3)_4(quin)_3$] (8). An analogous layering synthesis to that used for **1** was applied for preparing **8**, using an ethyl acetate solution (1.0 mL) of quinoxaline (6.5 mg, 0.05 mmol), a buffer solution of ethyl acetate (2 mL) and acetone (2 mL) and an acetone solution (1.0 mL) of silver(I) trifluoroacetate (20 mg, 0.09 mmol). Pale yellow crystals of **8** were formed within 2 days. Yield: 6.5 mg (60%). Anal. Calcd. for $C_{32}H_{18}Ag_4F_{12}N_6O_8$: C 30.15; H 1.41; N 6.60; found: C 30.14; H 1.70; N 6.56%.

Preparation of [$Ag_4(O_2CCF_2CF_3)_4(quin)_3$] (9). An analogous layering synthesis to that used for **1** was applied for preparing **9**, using an ethyl acetate solution (1.0 mL) of quinoxaline (6.0 mg, 0.046 mmol), a buffer solution of ethyl acetate (2 mL) and acetone (2 mL) and an acetone solution (1.0 mL) of silver(I) pentafluoropropanoate (16.8 mg, 0.062 mmol). Off-white crystals of **9** were formed within 3 days. Yield: 5.1 mg (23%). Anal. Calcd. for $C_{36}H_{18}Ag_4F_{20}N_6O_8$: C 29.35; H 1.22; N 5.70; found: C 29.29; H 1.12; N 5.62%.

Preparation of [$Ag_4(O_2C(CF_2)_2CF_3)_4(quin)_3$] (10). An analogous layering synthesis to that used for **1** was applied for preparing **10**, using an ethyl acetate solution (2.0 mL) of quinoxaline (13.0 mg, 0.10 mmol), a buffer solution of ethyl acetate (2 mL) and acetone (2 mL) and an acetone solution (2.0 mL) of silver(I) heptafluorobutanoate (58.0 mg, 0.18 mmol). Off-white crystals of **10** were formed within 3 days. Yield: 30 mg (58%). Anal. Calcd. for $C_{40}H_{18}Ag_4F_{28}N_6O_8$: C 28.68; H 1.08; N 5.02; found: C 28.92; H 1.10; N 5.06%.

Investigation of time-evolution of syntheses of [$Ag_4(O_2C(CF_2)_2CF_3)_4(quin)_4$] (3) and [$Ag_4(O_2C(CF_2)_2CF_3)_4(quin)_3$] (10)

An analogous layering synthesis to that used for **1-5** was used to explore the time-evolution of the synthesis of **3** and **10** to establish if interconversion takes places between the two over time. An ethyl acetate solution (2.0 mL) of quinoxaline (13.0 mg, 0.10 mmol), a buffer solution of ethyl acetate (2 mL) and acetone (2 mL) and an acetone solution (2.0 mL) of silver(I) heptafluorobutanoate (29.0 mg, 0.09 mmol) was allowed to react for 4 days before analysis of the solid product by PXRD showed a 48.9:51.1% ratio of **3**:**10**, established by Rietveld fitting (see section 4). A separate reaction set up under identical conditions was allowed to react for 7 days, whereupon analysis of the solid product by PXRD showed a 90.4:9.6% ratio of **3**:**10**, established by Rietveld fitting (see section 4).

Preparation of [$Ag_4(O_2C(CF_2)_2CF_3)_4(phen)_3$]. A 0.05 M solution of 128 mg (0.04 mmol) $Ag(O_2C(CF_2)_2CF_3)$ in ethanol was layered on to a 0.05 M solution of 72.1 mg (0.04 mmol) phenazine. Yellow crystals of [$Ag_4(O_2C(CF_2)_2CF_3)_4(phen)_3$] formed in 43% yield. Anal. Calcd. for $C_{52}H_{24}Ag_4F_{28}N_6O_8$: C 29.15; H 0.87; N 3.17; found: C 34.24; H 1.34; N 4.61%. Crystal structure details in Table S1.

S2. Single-Crystal X-ray Diffraction

Single-crystal X-ray data were collected at 150 K for **1**, **3-5**, **8** and **10**, 120 K for **2** and 100 K for **9** and **[$Ag_4(O_2C(CF_2)_2CF_3)_4$ (phen)$_3$]**, on a Bruker D8 *VENTURE* diffractometer, equipped with a PHOTON 100 CMOS detector (using Cu-K$_\alpha$ radiation, λ = 1.54178 Å) or a Bruker APEX-II diffractometer (using Mo-K$_\alpha$ radiation, λ = 0.71073 Å). The crystal structures were solved with

direct methods or Patterson methods using the *SHELXTL*[S1] or *Olex2*[S2] programs. All the structures were refined against all F^2 values and a multi-scan method (*SADABS*)[S3,S4] was used for absorption correction. Non-H atoms were refined with anisotropic displacement parameters, except where noted for disordered components. Hydrogen atoms were added at calculated positions and refined with a riding model and isotropic displacement parameters fixed in magnitude relative to the attached carbon atoms. Disordered parts in some compounds were modelled with reasonable occupancies and isotropic displacement parameters. Disorder of one CF_3 group in compound **9** was modelled with two orientations related by rotation (occupancy 0.71(4)/0.29(4)). Disorder of the four CF_3 groups in compound **8** was modelled with two orientations related for each (occupancy 0.50(3), 0.54(4)/0.46(4), 0.60(6)/0.40(6) and 0.66(6)/0.34(6)). Disorder of the $CF_2(CF_2)_4CF_3$ chains in compound **5** was modelled with two orientations (occupancy 0.58(1)/0.42(1)). Disorder of two out of total four $CF_2(CF_2)_2CF_3$ chains in compound **10** was modelled with two orientations (occupancy 0.59(2)/0.41(2) and 0.52(2)/0.48(2)). Disorder in the two independent perfluorcarboxylate groups was modelled in two orientations (occupancies 0.64(2)/0.36(2) and 0.50(2)/0.50(2)). The structure of **9** was modelled as a racemic twin (Flack *x* = 0.49(4)). Details of crystal data, structure solution and refinement parameters for compounds **1-10** and **[$Ag_4(O_2C(CF_2)_2CF_3)_4$ (phen)$_3$]** can be found in Table S1.

Table S1. Crystal Data, Structure Solution and Refinement Parameters

	**$[Ag_4(O_2CCF_3)_4(quin)_4]$ (1)**	**$[Ag_4(O_2CCF_2CF_3)_4(quin)_4]$ (2)**	**$[Ag_4(O_2C(CF_2)_2CF_3)_4(quin)_4]$ (3)**	**$[Ag_4(O_2C(CF_2)_3CF_3)_4(quin)_4]$ (4)**	**$[Ag_4(O_2C(CF_2)_4CF_3)_4(quin)_4]$ (5)**
CCDC deposition no.	2170223	2170224	2170225	2170230	2170226
Crystal colour	Yellow	Colourless	Yellow	Pale yellow	Yellow
Crystal size (mm)	0.22 x 0.26 x 0.05	0.20 x 0.14 x 0.04	0.34 x 0.22 x 0.13	0.34 x 0.19 x 0.02	0.38 x 0.10 x 0.07
Crystal system	Orthorhombic	Triclinic	Monoclinic	Monoclinic	Monoclinic
Space group, Z	$Pbcm$, 1	P-1, 1	$P2_1/c$, 1	$P2_1/c$, 1	$P2_1/c$, 1
a (Å)	11.7575(3)	13.3405(9)	14.5530(7)	17.0394(8)	17.0199(16)
b (Å)	6.6483(2)	6.6186(4)	6.6729(4)	6.6752(3)	6.6761(6)
c (Å)	14.4311(4)	14.3831(11)	14.3152(6)	14.2268(6)	14.3243(16)
α (°)	90	90.191(5)	90	90	90
β (°)	90	102.636(5)	96.466(3)	109.915(3)	94.823(7)
γ (°)	90	80.249(5)	90	90	90
V (Å^3)	1128.04(5)	1220.55(15)	1381.32(12)	1521.42(12)	1638.9(3)
Density (Mg.m^{-3})	2.067	2.182	2.169	2.178	2.225
Wavelength (Å)	0.71073	1.54178	0.71073	0.71073	0.71073
Temperature (K)	150	120	150	150	150
μ(Mo-K$_\alpha$/Cu-K$_\alpha$) (mm^{-1})	1.822	13.973	1.554	1.440	1.36
Θ range (°)	2.82 to 27.39	3.151 to 52.184	2.86 to 29.05	2.873 to 26.816	3.19 to 26.62
Reflns collected	10513	6712	11045	12678	12736
Independent reflns (R_{int})	1351 (0.0277)	2633 (0.0613)	3682 (0.0279)	3280 (0.0466)	3659 (0.0495)
Reflns used in refinement, n	1351	2633	3682	3280	3659
L.S. parameters, p	90	379	216	243	250
No. of restraints, r	0	1	0	0	55
$R1$ (F)[a] $I > 2.0\sigma(I)$	0.0223	0.0630	0.0391	0.0414	0.0861
$wR2(F^2)$[a], all data	0.0601	0.1765	0.1260	0.1264	0.2614
$S(F^2)$[a], all data	1.051	1.029	1.018	1.032	1.039

[a] $R1(F) = \Sigma(|F_o| - |F_c|)/\Sigma|F_o|$; $wR^2(F^2) = [\Sigma w(F_o^2 - F_c^2)^2/\Sigma wF_o^4]^{1/2}$; $S(F^2) = [\Sigma w(F_o^2 - F_c^2)^2/(n + r - p)]^{1/2}$

Table S1. (continued)

	$[Ag_4((O_2C)_2C_4F_8)_2(quin)_4]$ (6)	$[Ag_4(O_2CC_6F_5)_4(quin)_4]$ (7)	$[Ag_4(O_2CCF_3)_4(quin)_3]$ (8)	$[Ag_4(O_2CCF_2CF_3)_4(quin)_3]$ (9)	$[Ag_4(O_2C(CF_2)_2CF_3)_4(quin)_3]$ (10)	$[Ag_4(O_2C(CF_2)_2CF_3)_4(phen)_3]$
CCDC deposition no.	2170232	2170228	2170229	2170227	2170231	2170855
Crystal colour	Yellow	Yellow	Yellow	Yellow	White	Yellow
Crystal size (mm)	0.45 x 0.39 x 0.35	0.38 x 0.35 x 0.31	0.25 x 0.14 x 0.04	0.14 x 0.07 x 0.03	0.18 x 0.14 x 0.03	0.18 x 0.14 x 0.03
Crystal system	Monoclinic	Orthorhombic	Monoclinic	Orthorhombic	Monoclinic	Monoclinic
Space group, *Z*	*I*2/*a*, 2	*Pbcm*, 1	$P2_1/n$, 4	$P2_12_12$, 2	$P2_1/n$, 4	$P2_1/n$, 2
a (Å)	14.3254(16)	14.327(2)	10.3394(3)	10.5119(5)	10.6884(11)	14.9463(11)
b (Å)	6.6182(8)	6.6615(11)	16.3000(5)	16.3803(8)	16.4660(16)	8.5082(6)
c (Å)	23.507(3)	14.508(2)	23.2981(8)	12.3916(6)	27.817(3)	22.1370(16)
α (°)	90	90	90	90	90	90
β (°)	95.750(2)	90	97.992(2)	90	91.547(5)	93.293(3)
γ (°)	90	90	90	90	90	90
V (Å^3)	2217.5(4)	1384.6(4)	3888.24(2)	2133.7(2)	4893.8(9)	2810.4(4)
Density (Mg.m^{-3})	2.289	2.154	2.176	2.294	2.272	2.156
Wavelength (Å)	0.71073	0.71073	1.54178	1.54178	0.71073	0.71073
Temperature (K)	150	150	150	100	150	100
μ(Mo-K_α/Cu-K_α) (mm^{-1})	1.876	1.531	16.988	15.886	1.743	1.528
Θ range (°)	1.74 to 27.51	1.42 to 27.59	3.32 to 52.06	3.57 to 47.37	2.47 to 27.44	1.60 to 27.51
Reflns collected	11984	14793	21617	11113	37133	48308
Independent reflns (R_{int})	2543(0.0602)	1673(0.0487)	4261 (0.0628)	1918 (0.0630)	10782 (0.0700)	6448 (0.0394)
Reflns used in refinement, *n*	2543	1673	4261	1918	10782	6448
L.S. parameters, *p*	181	124	549	316	760	412
No. of restraints, *r*	0	0	0	312	46	60
$R1$ (F)[a] $I > 2.0\sigma(I)$	0.0432	0.0338	0.0620	0.0458	0.0391	0.0359
$wR2(F^2)$[a], all data	0.1147	0.0866	0.1574	0.1128	0.1260	0.1176
$S(F^2)$[a], all data	1.029	1.090	1.057	1.049	1.210	0.556

[a] $R1(F) = \Sigma(|F_o| - |F_c|)/\Sigma|F_o|$; $wR^2(F^2) = [\Sigma w(F_o^2 - F_c^2)^2/\Sigma wF_o^4]^{½}$; $S(F^2) = [\Sigma w(F_o^2 - F_c^2)^2/(n + r - p)]^{½}$

S3. Phase purity checks by powder X-ray diffraction (PXRD)

Powder X-ray diffraction data for phase purity checks were either recorded in University of Sheffield (for compound **5**, **8**, **9** and **10**) or Diamond Light Source Synchrotron (for compound **1**, **2**, **3** and **4**). The Bruker D8 ADVANCE X-ray powder diffractometer in University of Sheffield was fitted with a focusing Göbel mirror optic and a high-resolution energy-dispersive Lynxeye XE detector and operated in a capillary mode (Debye-Scherrer) or flat-plate mode (Bragg-Brentano) and using Cu-Kα radiation. For the capillary mode, the sample was packed in a 0.7 mm borosilicate capillary, whereas in the flat-plate mode each sample was loaded on a 14 mm silicon zero-background sample disc. Each sample was rotated at 30 rot min^{-1} to average the sample exposure and scanned at room temperature (collected as 4 sec $step^{-1}$ and 0.02° step size). The beamline I11 at Diamond Light Source[S5] was equipped with a wide angle (90°) PSD detector comprising 18 Mythen-2 modules (λ = 0.82562 Å). The samples were packed into 0.7 mm borosilicate capillaries and five pairs of scans were collected for 10 sec per pattern (2 x10 s per pair), as well as one 1 sec scan at both the start and the end of each pair for checking the exposure damage. These patterns were summed to offer the final collection pattern. All the patterns were collected at room temperature and diffraction patterns were indexed and fitted by Pawley refinement[S6] using the *TOPAS-Academic* program.[S7]

$[Ag_4(OOCCF_3)_4(quin)_4]$ (1)

The unit cell parameters from the single-crystal structure determination of compound **1** were used as a starting point for Pawley refinement, employing 444 parameters (6 background, 1 zero error, 5 profile, 3 cell, 429 reflections). Pawley refinement converged to R_{wp} = 0.0675, $R_{wp'}$ = 0.193 (Figure S1). [(**1**): a = 14.6765 (3) Å, b = 11.7985 (3) Å, c = 6.7539 (2) Å, V = 1169.51 (6) $Å^3$].

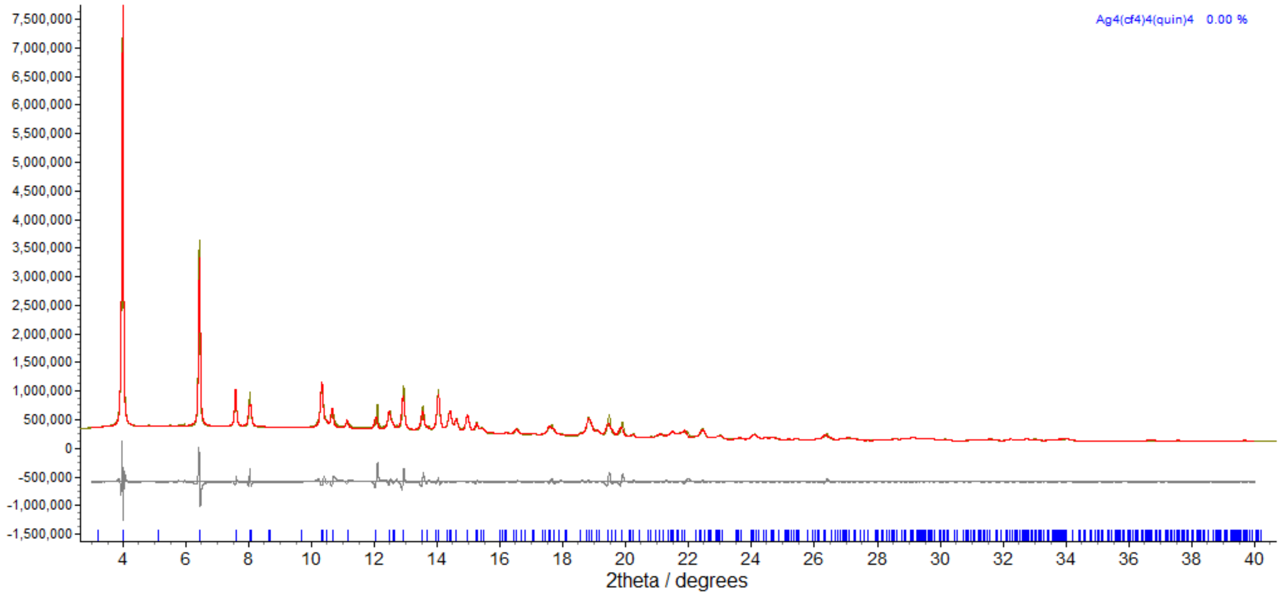

Figure S1. Observed (green) and calculated (red) profiles and difference plot $[I_{obs}-I_{calc}]$ (grey) of the Pawley refinement. (2θ range 3.0 - 40 °, d_{min} = 1.21 Å).

$[Ag_4(OOCCF_2CF_3)_4(quin)_4]$ (2)

The unit cell parameters from the single-crystal structure determination of compound **2** were used as a starting point for Pawley refinement, employing 3111 parameters (6 background, 1 zero error, 5 profile, 6 cell, 3093 reflections). Pawley refinement converged to $R_{wp} = 0.0445$, $R_{wp'} = 0.118$ (Figure S2). [**(2):** a = 22.1969 (9) Å, b = 11.2486 (4) Å, c = 25.9702 (7) Å, α = 85.446 (3) °, β = 135.633 (2) °, γ = 83.814 (3) °, V = 4392.2 (3) Å^3].

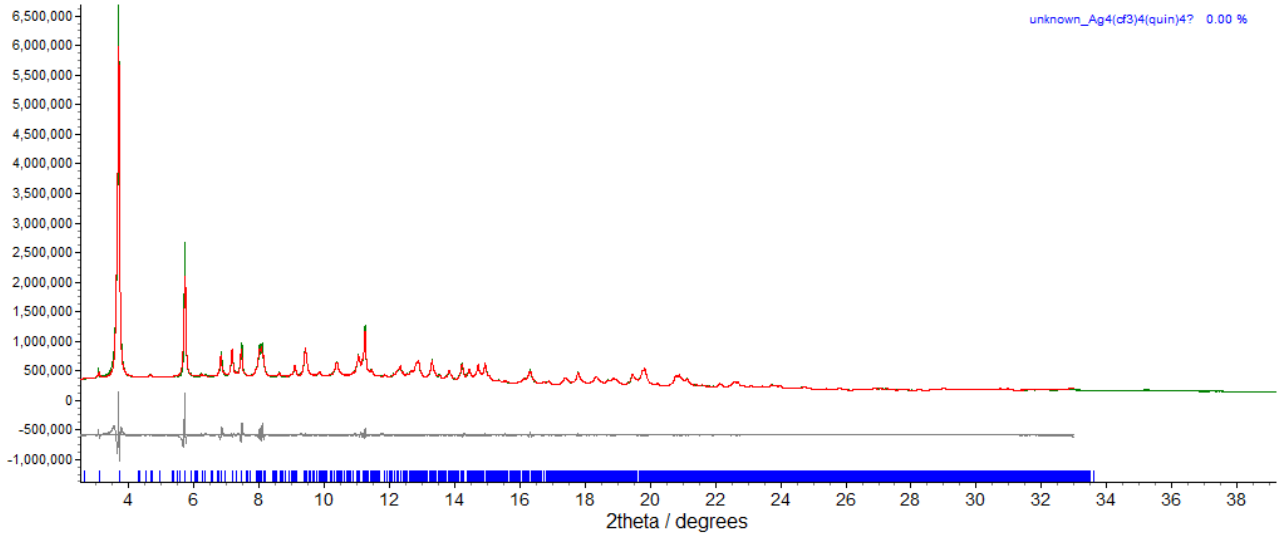

Figure S2. Observed (green) and calculated (red) profiles and difference plot [I_{obs}-I_{calc}] (grey) of the Pawley refinement. (2θ range 2.0 - 33 °, d_{min} = 1.45 Å).

$[Ag_4(OOC(CF_2)_2CF_3)_4(quin)_4]$ (3)

The unit cell parameters of from the single-crystal structure determination compound **3** were used as a starting point for the Pawley refinement, employing 338 parameters (6 background, 1 zero error, 5 profile, 4 cell, 322 reflections). Pawley refinement converged to R_{wp}= 0.0692, $R_{wp'}$ = 0.112 (Figure S3). [**(3):** a = 14.6410 (2) Å, b = 6.7869 (1) Å, c = 14.3907 (2) Å, β = 96.842 (2) °, V = 1419.78 (4) Å^3].

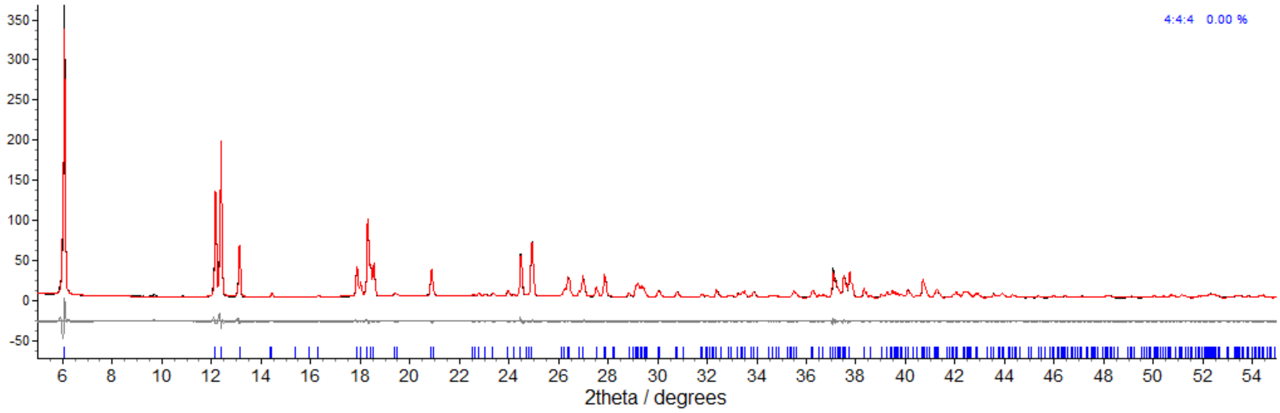

Figure S3. Observed (black) and calculated (red) profiles and difference plot [I_{obs}-I_{calc}] (grey) of the Pawley refinement. (2θ range 6.0 - 55 °, d_{min} = 0.89 Å).

$[Ag_4(OOC(CF_2)_3CF_3)_4(quin)_4]$ (4)

The unit cell parameters from the single-crystal structure determination of compound **4** were used as a starting point for Pawley refinement, employing 1825 parameters (6 background, 1 zero error, 5 profile, 4 cell, 1809 reflections). Pawley refinement converged to R_{wp} = 0.06956, $R_{wp'}$ = 0.181 (Figure S4). [**(4):** a = 18.3750 (3) Å, b = 6.78303 (7) Å, c = 14.3382 (1) Å, β = 61.400 (1) °, V = 1569.03 (4) Å^3].

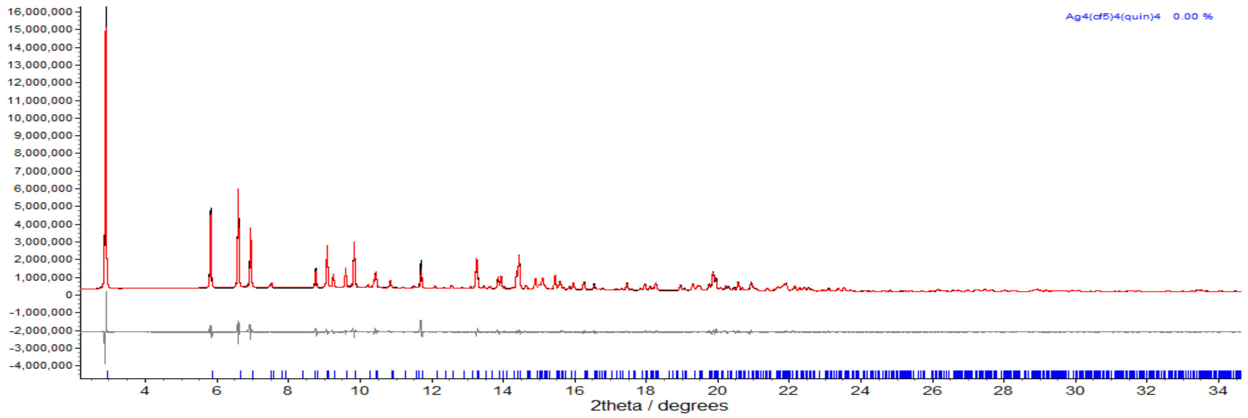

Figure S4. Observed (black) and calculated (red) profiles and difference plot [I_{obs}-I_{calc}] (grey) of the Pawley refinement. (2θ range 2.0 - 35 °, d_{min} = 1.37 Å).

$[Ag_4(OOC(CF_2)_4CF_3)_4(quin)_4]$ (5)

The sample was scanned at a rate of 1 sec step^{-1}, with each detector step size being 0.02°. The unit cell parameters from the single-crystal structure determination of compound **5** were used as a starting point for Pawley refinement, employing 480 parameters (14 background, 1 zero error, 5 profile, 4 cell, 456 reflections). Pawley refinement converged to R_{wp}= 0.051, $R_{wp'}$ = 0.157 (Figure S5). [**(5):** a = 17.288 (9) Å, b = 6.883 (1) Å, c = 14.479 (6) Å, β = 95.18 (2) °, V = 1715.9 (12) Å^3].

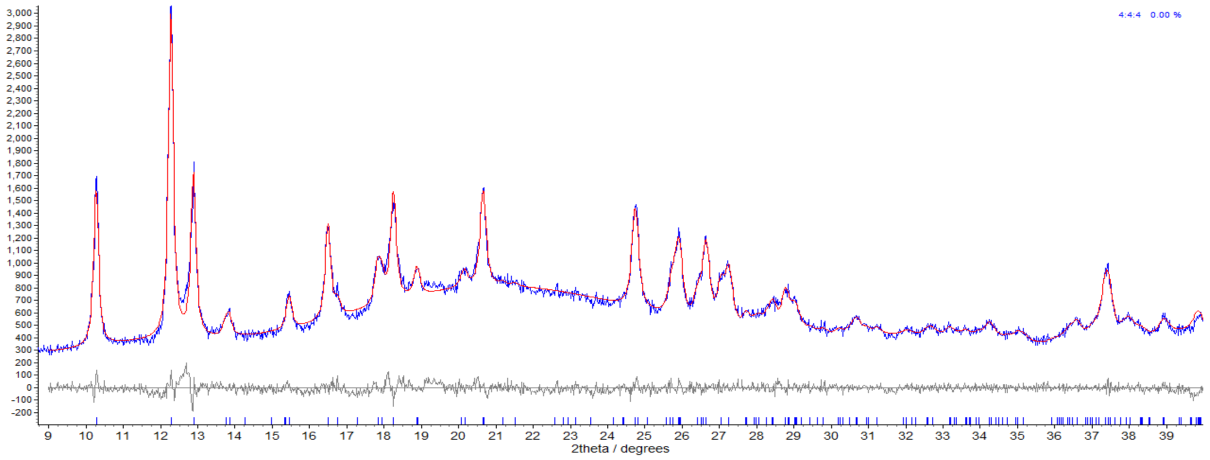

Figure S5. Observed (blue) and calculated (red) profiles and difference plot [I_{obs}-I_{calc}] (grey) of the Pawley refinement. (2θ range 9 - 40 °, d_{min} = 2.25 Å).

$[Ag_4(OOCCF_3)_4(quin)_3]$ (8)

The sample was scanned at a rate of 1 sec step^{-1}, with each detector step size being 0.02°. The unit cell from the single-crystal structure determination of compound **8** were used as a starting point for a Pawley refinement, employing 384 parameters (6 background, 1 zero error, 5 profile, 4 cell, 368 reflections), resulting in final indices of fit R_{wp}= 0.0674, $R_{wp'}$ = 0.281 (Figure S6). [**(8):** *a* = 10.164 (4) Å, *b* = 16.239 (3) Å, *c* = 23.582 (3) Å, β = 99.27 (4) °, *V* = 3841 (2) Å^3].

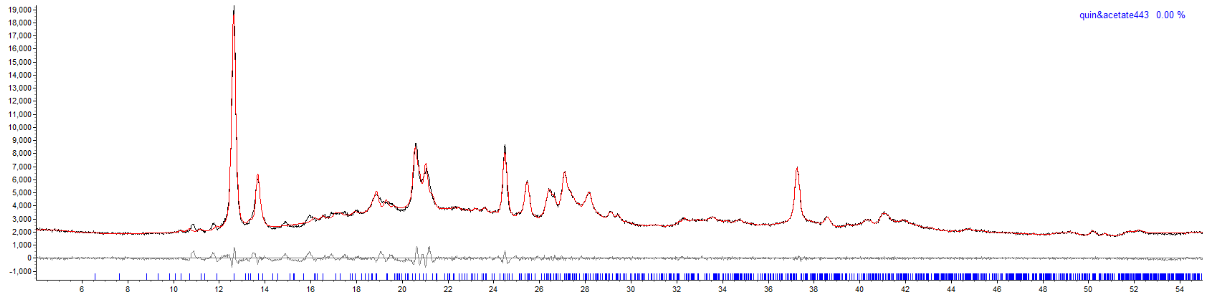

Figure S6. Observed (black) and calculated (red) profiles and difference plot [I_{obs}-I_{calc}] (grey) of the Pawley refinement. (2θ range 4 - 55 °, d_{min} = 1.67 Å).

$[Ag_4(OOCCF_2CF_3)_4(quin)_3]$ (9)

The sample was scanned at a rate of 1.5 sec step^{-1}, with each detector step size being 0.02°. The unit cell parameters from the single-crystal structure determination of compound **9** were used as a starting point for the Pawley refinement, employing 420 parameters (15 background, 1 zero error, 5 profile, 4 cell, 395 reflections). Pawley refinement converged to R_{wp}= 0.0750, $R_{wp'}$ = 0.111 (Figure S7). [**(9):** *a* = 10.649 (9) Å, *b* = 16.49 (1) Å, *c* = 12.715 (2) Å, *V* = 2232 (2) Å^3].

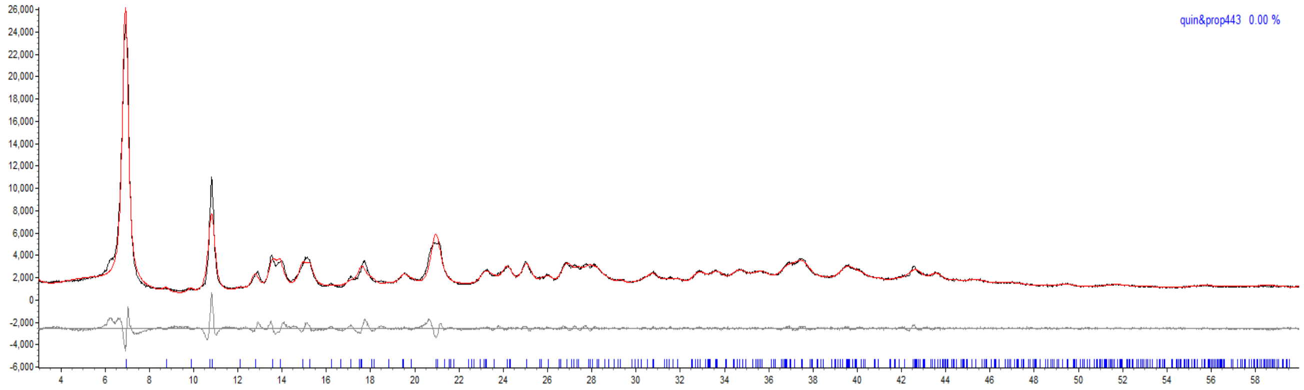

Figure S7. Observed (black) and calculated (red) profiles and difference plot [I_{obs}-I_{calc}] (grey) of the Pawley refinement. (2θ range 3.0 - 60 °, d_{min} = 1.54 Å).

$[Ag_4(OOC(CF_2)_2CF_3)_4(quin)_3]$ (10)

The sample was scanned at a rate of 1.5 sec step^{-1}, with each detector step size being 0.02°. The unit cell parameters from the single-crystal structure determination of compound **10** were used as a starting point for the Pawley refinement, employing 1453 parameters (15 background, 1 zero error, 5 profile, 4 cell, 1428 reflections). Pawley refinement converged to R_{wp} = 0.0513, $R_{wp'}$ = 0.0891 (Figure S8). [**(10):** *a* = 10.6996 (9) Å, *b* = 16.312 (1) Å, *c* = 27.977 (2) Å, β = 92.364 (7) °, *V* = 4878.8 (7) Å^3].

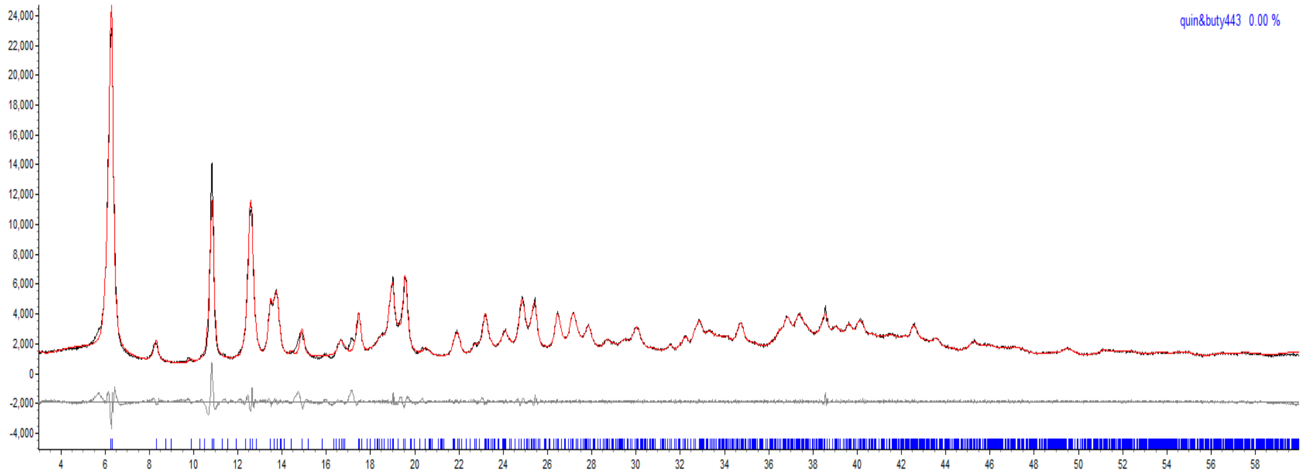

Figure S8. Observed (black) and calculated (red) profiles and difference plot [I_{obs}-I_{calc}] (grey) of the Pawley refinement. (2θ range 3.0 - 60 °, d_{min} = 1.54 Å).

S4. PXRD studies of time-evolution of synthesis of 3 and 10

PXRD data were collected for product of the reaction of quinoxaline with silver(I) heptafluorobutanoate to monitor the production of $[Ag_4(O_2C(CF_2)_2CF_3)_4(quin)_4]$ (**3**) and $[Ag_4(O_2C(CF_2)_2CF_3)_4(quin)_3]$ (**10**) as function of reaction time (see section 1 for reaction conditions). Two studies were undertaken, at time points following reaction after 4 days and after 7 days. Patterns were collected at room temperature and fitted using the *TOPAS-Academic* program.

For the product after 4 days' reaction, the powder was loaded into a 0.5 mm borosilicate capillary. PXRD data were collected using Cu-Kα radiation at University of Sheffield, Department of Materials Science on a STOE STADI P equipped with a PSD detector. A single scan ($5 \leq 2\theta \leq 40$ °) was measured at a scan rate of 0.067 ° min^{-1}, using a rotating capillary. The unit cell parameters for **3** and **10** from single-crystal diffraction studies were used as starting points for a two-phase Pawley refinement, which in turn provided a starting point for a two-phase Rietveld refinement[S8] for which the structure models were kept fixed as those from the single-crystal structure determinations, except for the refinement of a global isotropic thermal parameter. This was refined along with parameters for background, zero error, profile, unit cell, and scale. The model converged to a fit (R_{wp} = 0.0632, $R_{wp'}$ = 0.2321) with a 48.9:51.1% ratio of **3**:**10** (Figure S9).

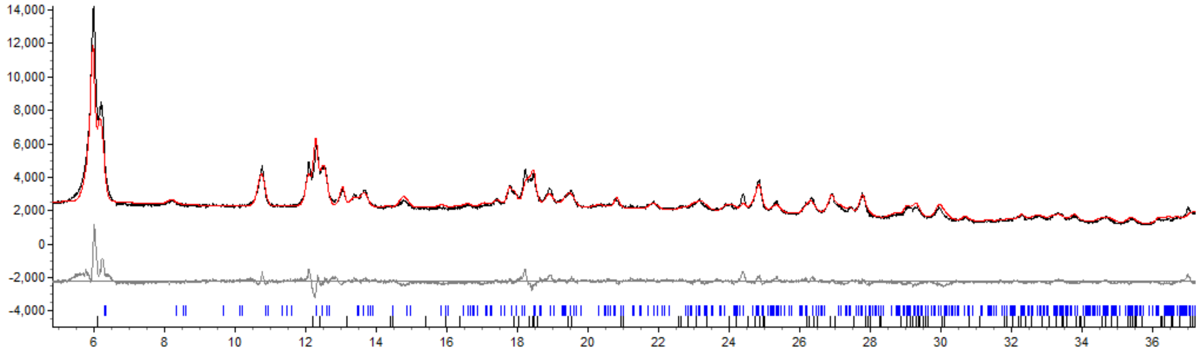

Figure S9. Observed (black), calculated (red) profiles and difference plot [I_{obs}-I_{calc}] (grey) of the two-phase Rietveld refinement. (2θ range shown 5 – 38 °, d_{min} = 2.37 Å).

For the product after 7 days' reaction the powder was loaded into a 0.7 mm quartz capillary. PXRD data were collected (λ = 0.82665 Å) at beamline I11 at Diamond Light Source, equipped with a wide angle (90 °) PSD detector comprising multiple Mythen-2 modules. Pairs of scans was conducted at room temperature, each pair related by a 0.25 ° detector offset to account for gaps between detector modules. The resulting patterns were summed to give the final pattern for structural analysis. Pawley and Rietveld fitting of the pattern were conducted in an analogous manner to the sample studied after 4 days' reaction. The final model converged to a Rietveld fit (R_{wp} = 0.0817, $R_{wp'}$ = 0.1817) with a 90.4:9.6 1% ratio of **3**:**10** (Figure S10).

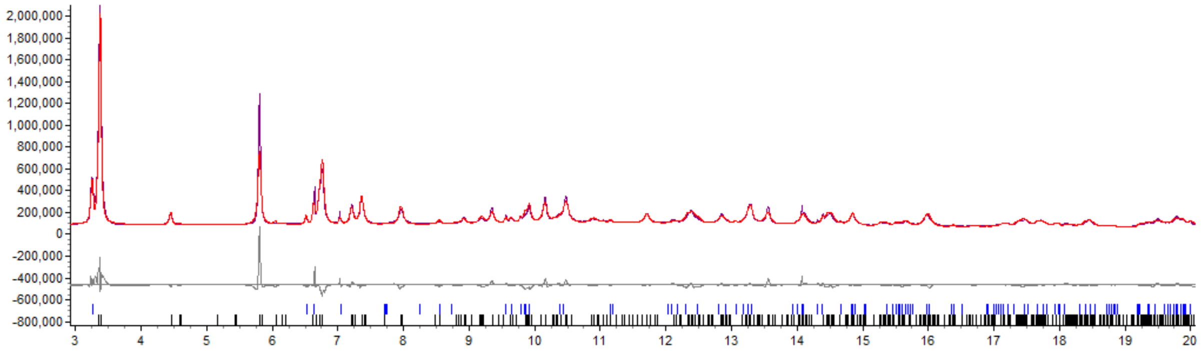

Figure S10. Observed (blue), calculated (red) and difference [I_{obs}-I_{calc}] (grey) profiles of two-phase Rietveld refinement (2θ range 3.0 – 20 °, d_{min} = 2.38 Å).

S5. Thermogravimetric analyses (TGA)

Thermogravimetric analyses were recorded on a Perkin-Elmer Pyris1 TGA model thermogravimetric analyser. Samples were heated under a flow of dry N_2 gas. In each case the component lost at each heating stage and the residue remaining is suggested based on mass loss and not independently verified by spectroscopic measurements.

[$Ag_4(O_2CCF_3)_4$(quin)$_4$] (1)

Temperature sweep TGA: The sample (6.91 mg) was held at 25 °C for 5 mins and then heated to 500 °C at a rate at 2.5 °C/min, followed by holding the temperature at 500 °C for another 5 mins (Figure S11, Table S2).

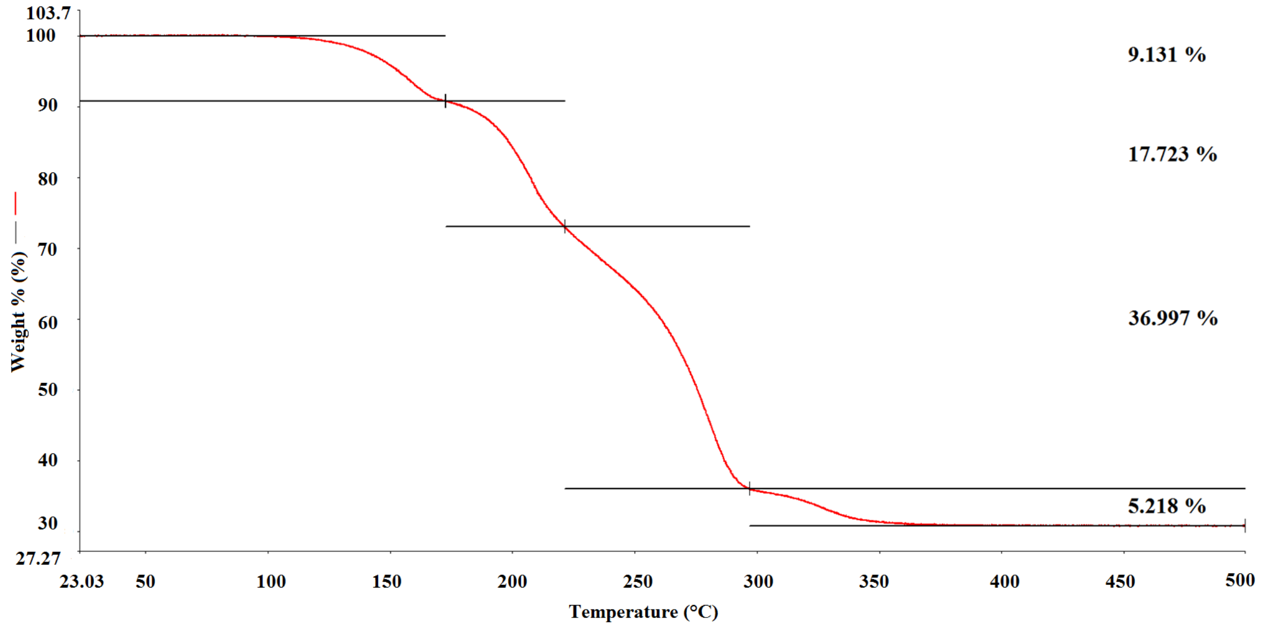

Figure S11. TGA for [$Ag_4(O_2CCF_3)_4$(quin)$_4$] at a scan rate of 5 °C/min.

Table S2. Temperature-sweep TGA mass losses for **[$Ag_4(O_2CCF_3)_4$(quin)$_4$].**

Onset Temperature (°C)	Lost component	Calculated Mass Contribution (%)	Observed Mass Loss Δm (%)	Residue
125	quin	9.27	9.131	$Ag_4(O_2CCF_3)_4$ (quin)$_3$
175	2(quin)	18.54	17.723	$Ag_4(O_2CCF_3)_4$ (quin)
225	quin, 2($COCF_3$), 2(CF_3), CO_2	36.01	36.997	Ag_2O, Ag_2CO_3
290	CO_2, O_2	5.41	5.218	Ag

Isothermal TGA: The sample (5.23 mg) was heated to 135 °C with a heating rate of 10 °C/min and then held at this temperature for 180 mins (Figure S12).

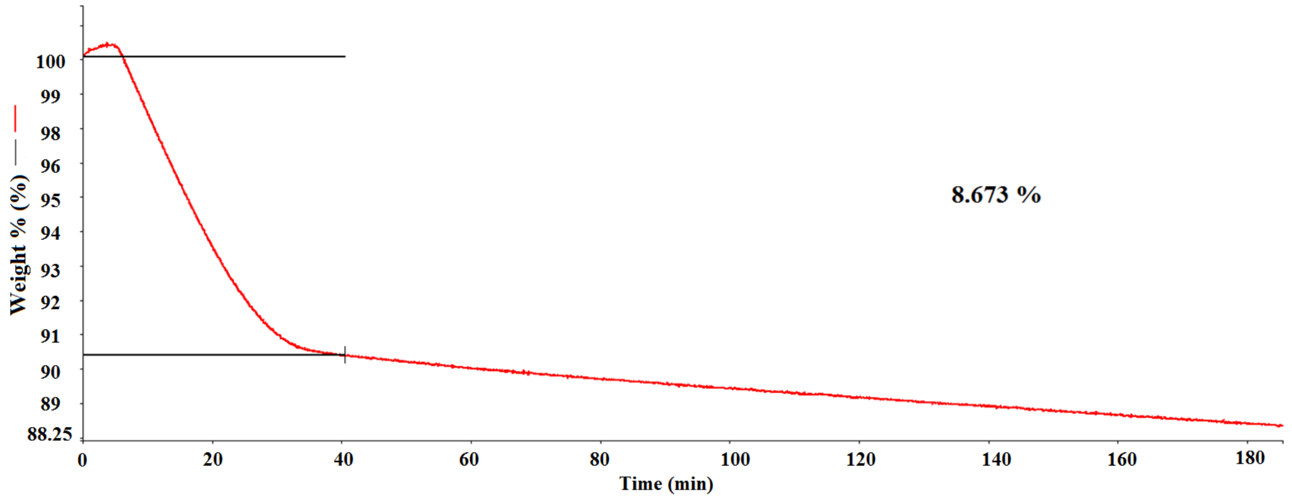

Figure S12. TGA for **[$Ag_4(O_2CCF_3)_4$(quin)$_4$]** at a constant temperature of 135 °C.

[$Ag_4(O_2CCF_2CF_3)_4$(quin)$_4$] (2)

Temperature sweep TGA: The sample (3.31 mg) was held at 25 °C for 5 mins and then heated to 480 °C at a rate at 2.5 °C/min, followed by holding the temperature at 480 °C for another 5 mins (Figure S13, Table S3).

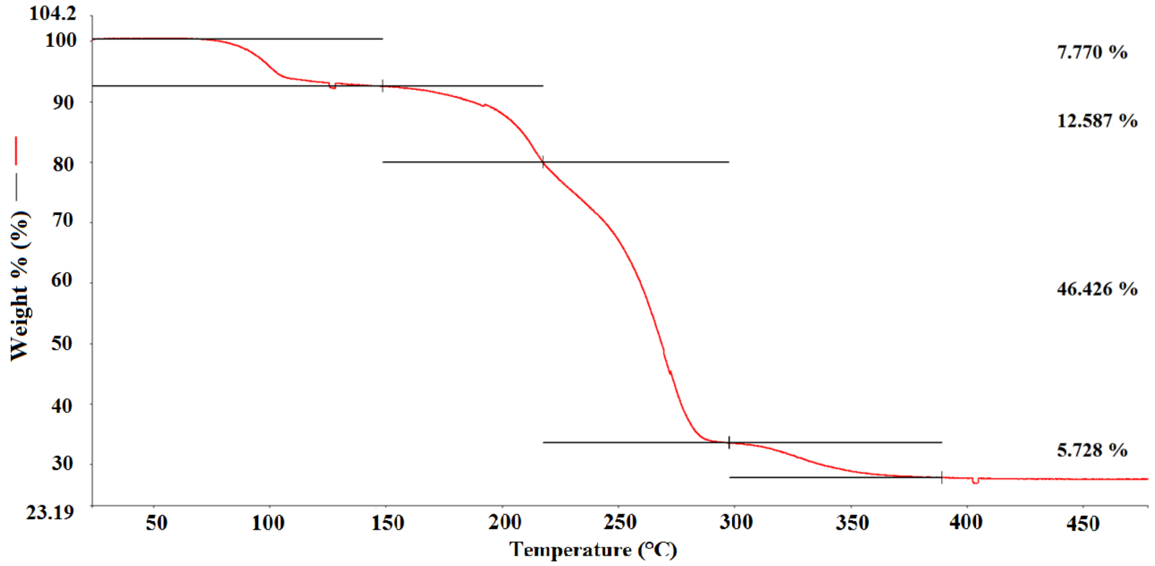

Figure S13. TGA for **[$Ag_4(O_2CCF_2CF_3)_4$(quin)$_4$]** at a scan rate of 2.5 °C/min.

Table S3. Temperature-sweep TGA mass losses for **[$Ag_4(O_2CCF_2CF_3)_4$(quin)$_4$]**.

Onset Temperature (°C)	Lost Component	Calculated Mass Contribution (%)	Observed Mass Loss Δm (%)	Residue
85	quin	8.12	7.770	[$Ag_4(OOCCF_2CF_3)_4$ (quin)$_3$]
150	1.5(quin)	12.17	12.587	[$Ag_4(OOCCF_2CF_3)_4$ (quin)$_{1.5}$]
210	1.5(quin), 2($COCF_2CF_3$), 2(CF_2CF_3)	46.49	46.426	$2Ag_2CO_3$
300	$2CO_2$	5.49	5.728	$2Ag_2O$

Isothermal TGA: Sample (6.12 mg) was heated to 95 °C with a heating rate of 10 °C/min and then held at this temperature for 120 mins (Figure S14).

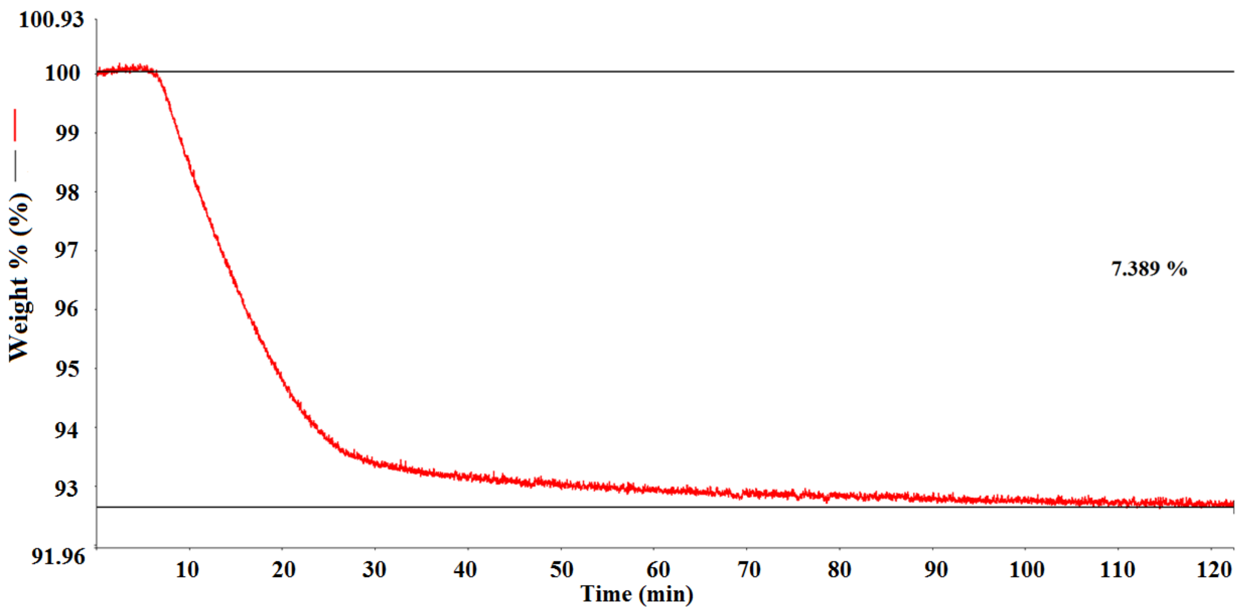

Figure S14. TGA for **[$Ag_4(O_2CCF_2CF_3)_4(quin)_4$]** at a constant temperature of 95 °C.

[$Ag_4(O_2C(CF_2)_3CF_3)_4(quin)_4$] (3)

Temperature sweep TGA: Sample (4.16 mg) was held at 25 °C for 5 mins and then heated to 480 °C at a rate at 2.5 °C/min followed by holding the temperature at 480 °C for another 5 mins (Figure S15, Table S4).

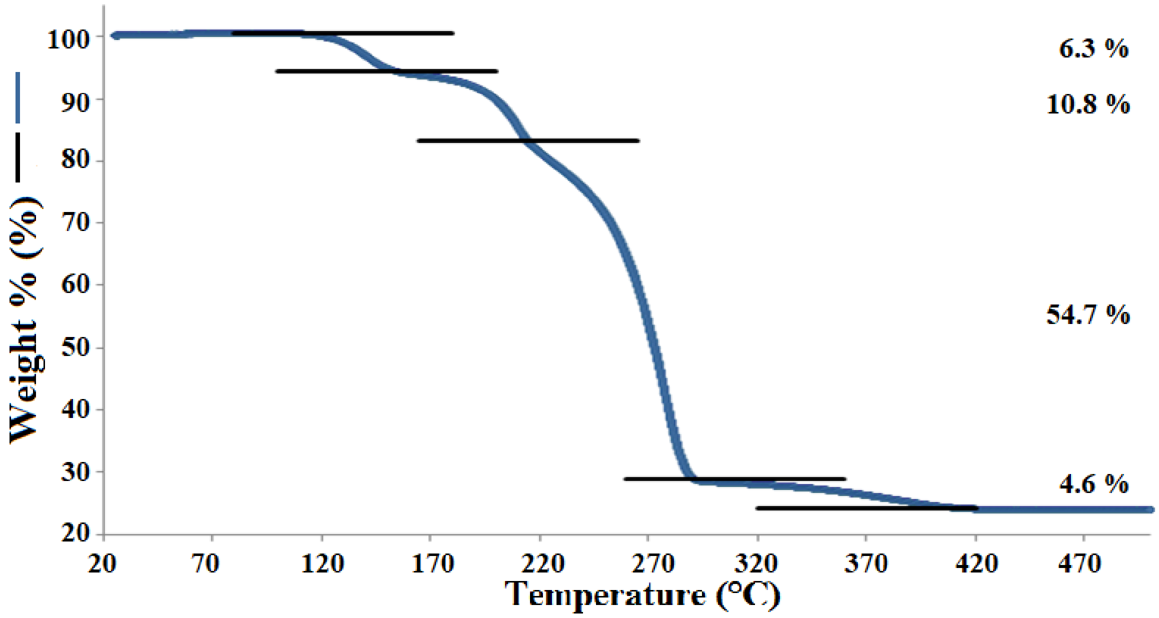

Figure S15. TGA for **[$Ag_4(O_2C(CF_2)_2CF_3)_4(quin)_4$]** at a scan rate of 2.5 °C/min.

Table S4. Temperature-sweep TGA mass losses for **[$Ag_4(O_2C(CF_2)_2CF_3)_4(quin)_4$]**.

Onset Temperature (°C)	Lost component	Calculated Mass Contribution (%)	Observed Mass Loss Δm (%)	Residue
85	quin	7.2	6.3	$Ag_4(OOC(CF_2)_3CF_3)_4$ (quin)$_3$
150	1.5(quin)	10.8	10.8	$Ag_4(OOC(CF_2)_3CF_3)_4$ (quin)$_{1.5}$
210	1.5(quin), 2($CO(CF_2)_2CF_3$), 2($(CF_2)_2CF_3$)	53.0	54.7	$2Ag_2CO_3$
290	$2CO_2$	4.8	4.6	$2Ag_2O$

Isothermal TGA: Sample (3.85 mg) was heated to 120 °C with a heating rate of 10 °C/min and then held at this temperature for 140 mins (Figure S16).

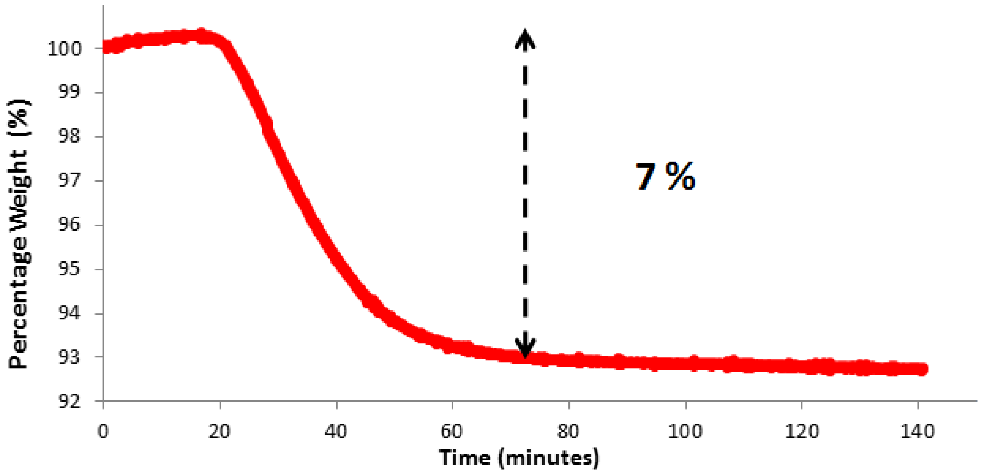

Figure S16. TGA for **[$Ag_4(O_2C(CF_2)_2CF_3)_4(quin)_4$]** at constant temperature of 120 °C.

[$Ag_4(O_2C(CF_2)_3CF_3)_4(quin)_4$] (4)

Temperature sweep TGA: Sample (5.83 mg) was held at 25 °C for 5 mins and then heated to 550 °C at a rate at 2.5 °C/min followed by holding the temperature at 550 °C for another 5 mins (Figure S17, Table S5).

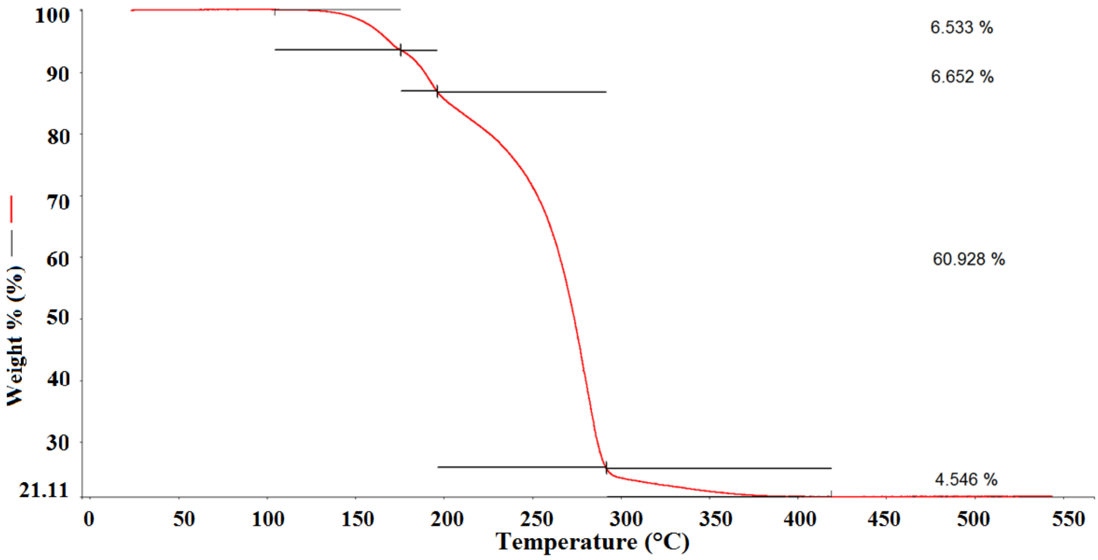

Figure S17. TGA for **[$Ag_4(O_2C(CF_2)_3CF_3)_4(quin)_4$]** at a scan rate of 2.5 °C/min.

Table S5. Temperature-sweep TGA mass losses for **[$Ag_4(O_2C(CF_2)_3CF_3)_4$(quin)$_4$]**.

Onset Temperature (°C)	Lost Component	Calculated Mass Contribution (%)	Observed Mass Loss Δm (%)	Residue
125	quin	6.49	6.533	[$Ag_4(OOC(CF_2)_3CF_3)_4$ (quin)$_3$]
175	quin	6.49	6.652	[$Ag_4(OOC(CF_2)_3CF_3)_4$ (quin)$_2$]
195	2(quin) 2($CO(CF_2)_3CF_3)_4$), 2($(CF_2)_3CF_3)_4$)	59.50	60.928	2Ag_2CO_3
290	2CO_2	4.39	4.546	2Ag_2O

Isothermal TGA: Sample (7.08/7.15/6.87 mg) was heated to 130/120/115 °C with a heating rate of 10 °C/min and then held at this temperature for 180/300/400 mins (Figures S18-S20).

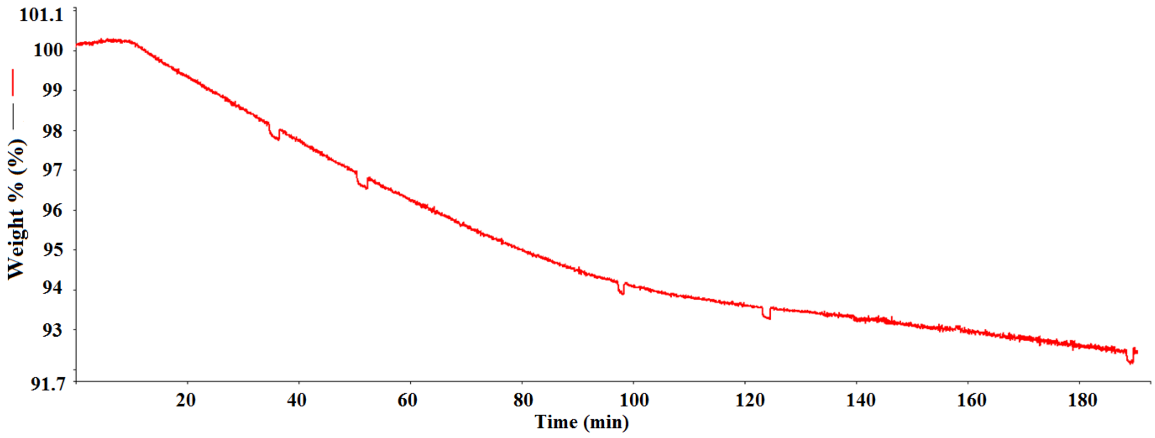

Figure S18. TGA for **[$Ag_4(O_2C(CF_2)_3CF_3)_4$ (quin)$_4$]** at a constant temperature of 130 °C.

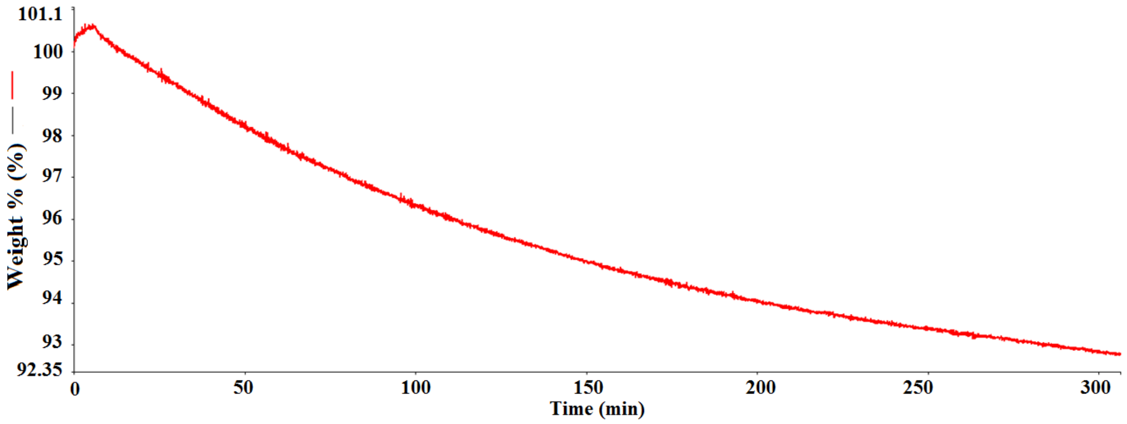

Figure S19. TGA for **[$Ag_4(O_2C(CF_2)_3CF_3)_4$(quin)$_4$]** at a constant temperature of 120 °C.

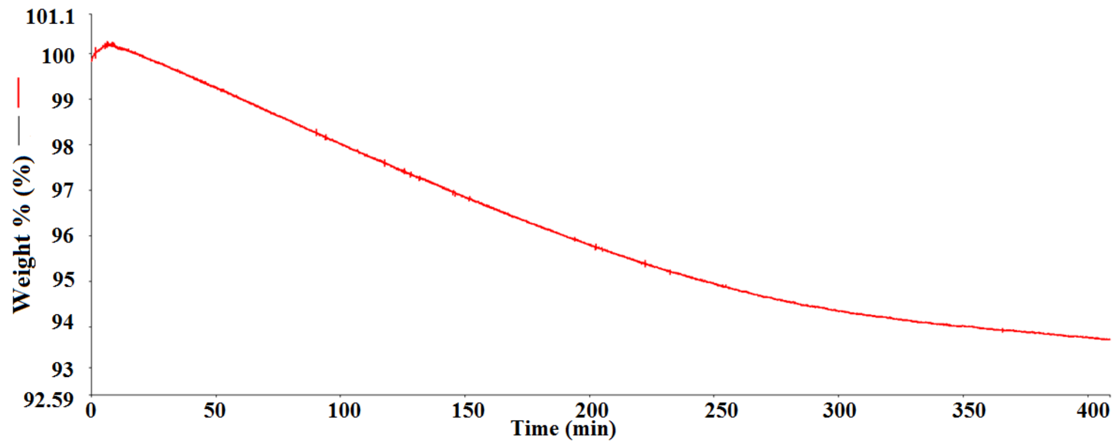

Figure S20. TGA for **[$Ag_4(O_2C(CF_2)_3CF_3)_4(quin)_4$]** at a constant temperature of 115 °C.

[$Ag_4(O_2C(CF_2)_4CF_3)_4(quin)_4$] (5)

Temperature sweep TGA: The sample (7.45 mg) was held at 25 °C for 5 mins and then heated to 500 °C at a rate at 5 °C/min followed by holding the temperature at 500 °C for another 5 mins (Figure S21, Table S6).

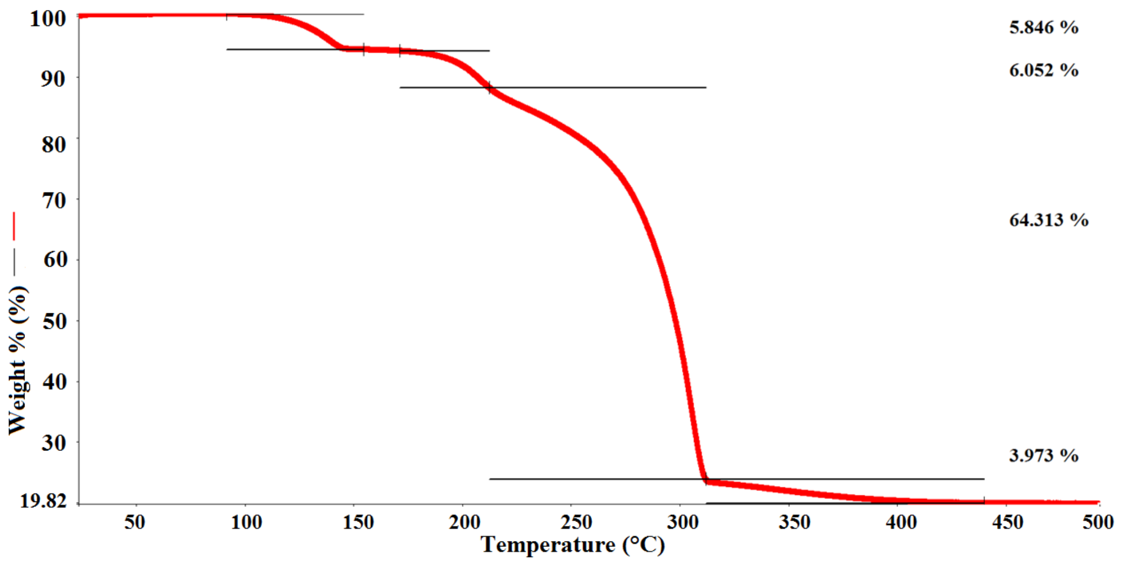

Figure S21. TGA for **[$Ag_4(O_2C(CF_2)_4CF_3)_4$ $(quin)_4$]** at a scan rate of 5 °C/min.

Table S6. Temperature-sweep TGA mass losses for **[$Ag_4(O_2C(CF_2)_4CF_3)_4(quin)_4$]**.

Onset Temperature (°C)	Lost Component	Calculated Mass Contribution (%)	Observed Mass Loss Δm (%)	Residue
100	quin	5.91	5.846	[$Ag_4(OOC(CF_2)_4CF_3)_4$ $(quin)_3$]
170	quin	5.91	6.052	[$Ag_4(OOC(CF_2)_4CF_3)_4$ $(quin)_2$]
220	2(quin), 2($CO(CF_2)_4CF_3$)), 2(($CF_2)_4CF_3$))	63.18	64.313	2Ag_2CO_3
315	2CO_2	3.99	3.973	2Ag_2O

Isothermal TGA: The sample (5.97 mg) was heated to 120 °C with a heating rate of 10 °C/min and then held at this temperature for 120 mins (Figure S22).

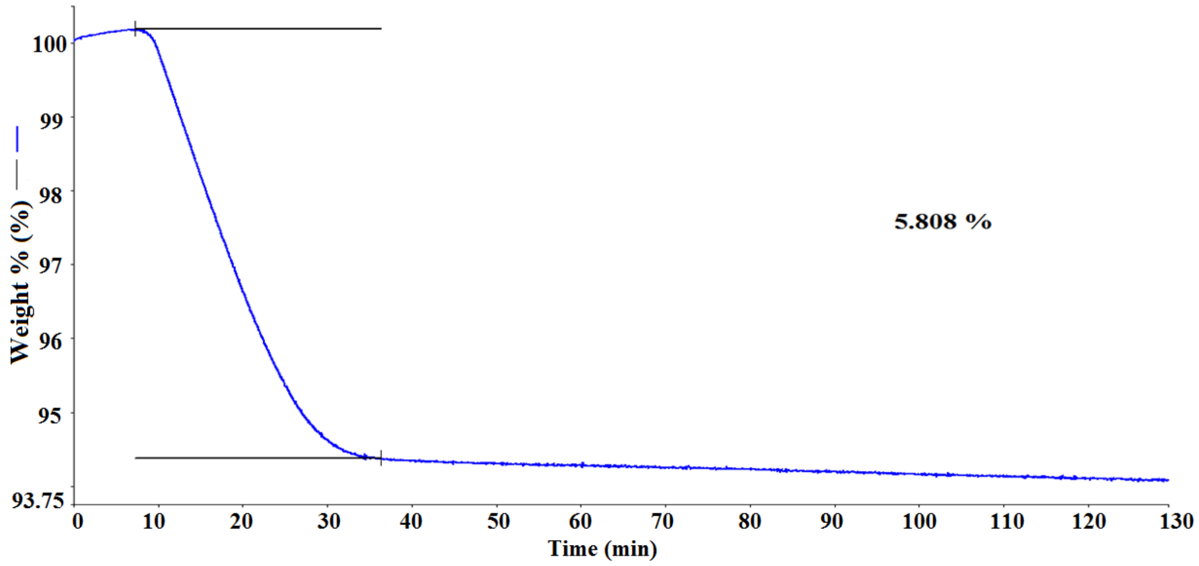

Figure S22. TGA for **[$Ag_4(O_2C(CF_2)_4CF_3)_4(quin)_4$]** at a constant temperature of 120 °C.

S6. *In situ* PXRD heating studies of loss of quinoxaline from 1, 3 and 5

In situ PXRD heating studies were recorded on a Bruker D8 ADVANCE X-ray powder diffractometer equipped with a focusing Göbel mirror optic and a high-resolution energy-dispersive Lynxeye XE detector. Samples were loaded in a 0.7 mm borosilicate capillary which was open at both ends and rotated at 30 rev min^{-1}. Measurements started with a collection at 298 K. Samples were heated up to 433 K, using an Oxford Cryosystems Cryostream Plus device with nitrogen flow co-axial with the capillary. Several more collections were undertaken at 433 K, followed by a final collection after cooling back to 298 K. The patterns were indexed and fitted using Pawley refinement using the *TOPAS Academic* program.

[$Ag_4(O_2CCF_3)_4(quin)_4$] (1) → [$Ag_4(O_2CCF_3)_4(quin)_3$] (8)

A stack plot of the series of PXRD patterns is shown in Figure 7a in the main paper for the study of the conversion of **1** to **8**. Patterns at 298 K were measured in the range $6 \leq 2\theta \leq 50$ ° using a step size of 0.015° and step time of 2.5 s, giving a total exposure time of 94 mins. No scans were collected during heating to 433 K. Patterns at 433 K were measured in the range $3 \leq 2\theta \leq 40$ ° using a step size of 0.015 ° and step time of 0.5 s, giving a total exposure time of 19 mins. The patterns are presented in the range 7-40 ° for consistency with the 298 K patterns. Patterns were compared to the calculated PXRD patterns from the single-crystal structures of **1** and **8** and fitted using Pawley refinement. The starting material and the final product at 298 K were confirmed to be phase-pure **1** and **8**, respectively.

Initial pattern at 298 K

The pattern was indexed, and a unit cell resembling that of **1** obtained from single-crystal X-ray data at 150 K was found. This unit cell was used as the starting point for a Pawley refinement, employing 130 parameters (10 background, 1 zero error, 5 profile, 3 cell, 111 reflections), resulting in final

indices of fit R_{wp} = 0.0445, $R_{wp'}$ = 0.162 (Figure S23). [**(1):** a = 11.7080(7) Å, b = 6.7367(7) Å, c = 14.6158(9) Å, V = 1152.8(2) Å^3].

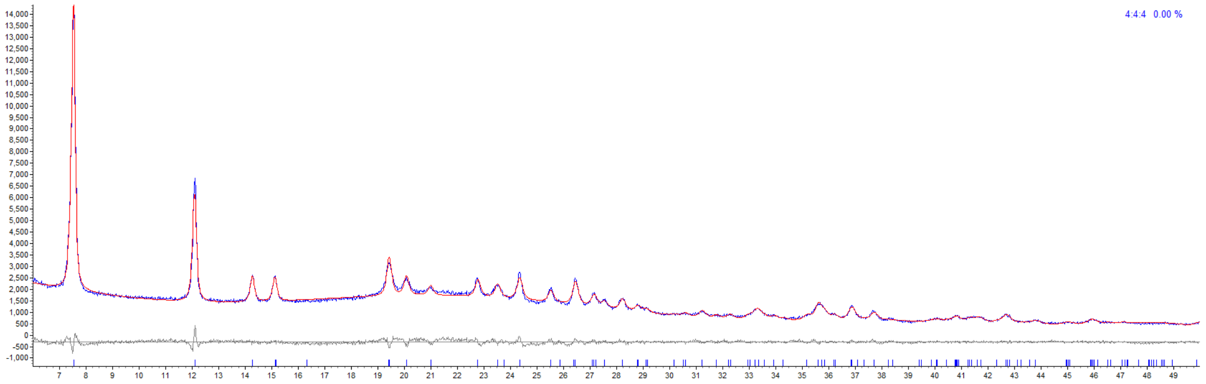

Figure S23. Observed (blue) and calculated (red) profiles and difference plot [I_{obs}-I_{calc}] (grey) of the Pawley refinement. (2θ range 7 - 50°, d_{min} = 1.82 Å).

After 1 hour at 433 K.

The sample was heated to 433 K and allowed to equilibrate at this temperature for one hour before the pattern was recorded. Visual inspection and comparison of the data indicated the presence of both **1** and **8**. The unit cells of the 4:4:4 phase (**1**) from the refinement at 298 K and that of the 4:4:3 phase (**8**) from a later point in the study at 433 K (when it was the only phase present) were used as the starting points for a mixed-phase Pawley refinement, employing 452 parameters (8 background, 1 zero error, 9 profile, 7 cell, 427 reflections), resulting in final indices of fit R_{wp} = 0.0670, $R_{wp'}$ = 0.166 (Figure S24). [**(1):** a = 11.848(2) Å, b = 6.783(2) Å, c = 14.620(5) Å, V = 1174.8(5) Å^3; **(8):** a = 10.1389(11) Å, b = 16.244(2) Å, c = 23.6611(12) Å, β = 99.249(6) °, V = 3846.3(6) Å^3].

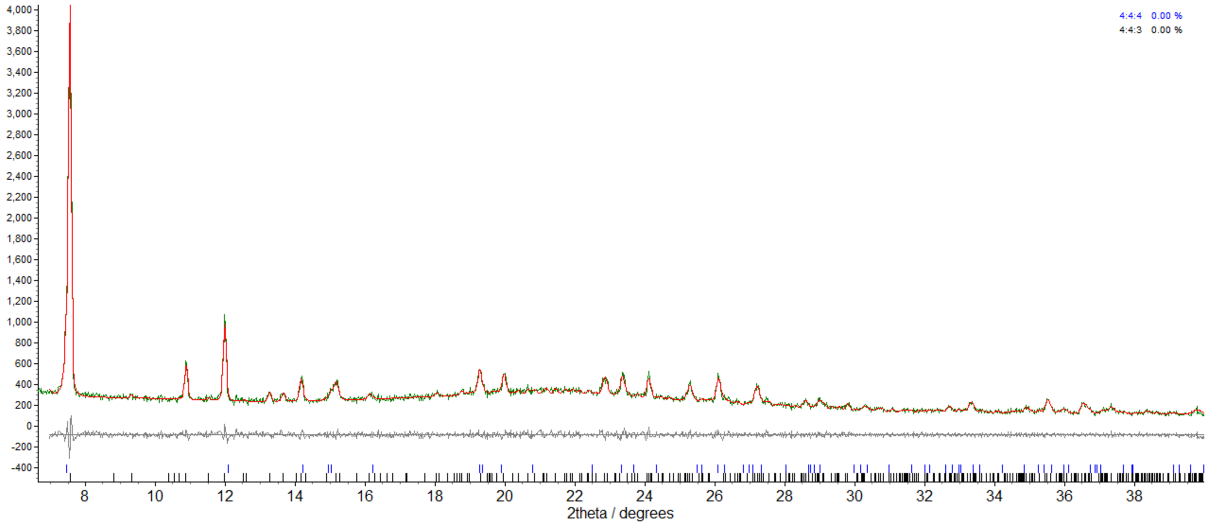

Figure S24. Observed (green) and calculated (red) profiles and difference plot [I_{obs}-I_{calc}] (grey) of the Pawley refinement. (2θ range 7 - 40°, d_{min} = 2.25 Å). Blue and black tickmarks correspond to reflections for **1** and **8**, respectively.

After 2 hours 15 min at 433 K.

The sample was kept at 433 K for a further one hour and 15 minutes. The pattern was recorded again. Visual inspection and comparison of the data indicated the presence of both **1** and **8**. The unit cells of both phases from the previous refinement were used as the starting point for a mixed-phase Pawley

refinement, employing 452 parameters (8 background, 1 zero error, 9 profile, 7 cell, 427 reflections), resulting in final indices of fit R_{wp} = 0.0666, $R_{wp'}$ = 0.1805 (Figure S25). [**(1):** *a* = 11.834 (2) Å, *b* = 6.800 (2) Å, *c* = 14.620 (5) Å, *V* = 1176.5 (5) Å^3; **(8):** *a* = 10.1469 (13) Å, *b* = 16.245 (2) Å, *c* = 23.6508 (12) Å, β = 99.284 (7) °, *V* = 3847.4 (7) Å^3].

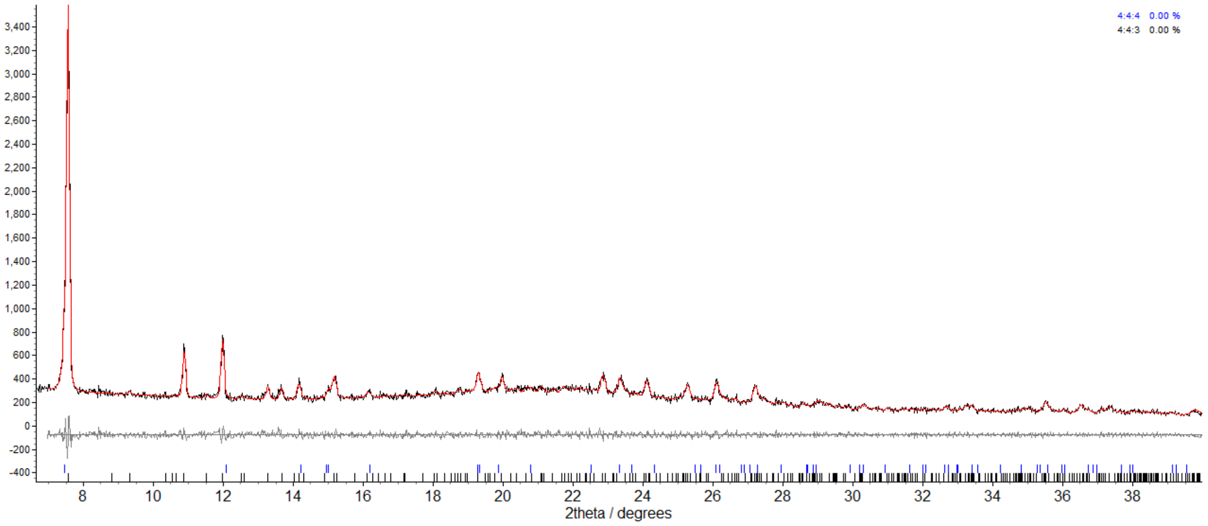

Figure S25. Observed (black) and calculated (red) profiles and difference plot [I_{obs}-I_{calc}] (grey) of the Pawley refinement. (2θ range 7 - 40 °, d_{min} = 2.25 Å). Blue and black tickmarks correspond to reflections for **1** and **8**, respectively.

After 3 hours 30 min at 433 K.

The sample was kept at 433 K for a further one hour and 15 minutes. The pattern was recorded again. Visual inspection and comparison of the data indicated the presence of both **1** and **8**. The unit cells of both phases from the previous refinement were used as the starting point for a mixed-phase Pawley refinement, employing 452 parameters (8 background, 1 zero error, 9 profile, 7 cell, 427 reflections), resulting in final indices of fit R_{wp} = 0.0683, $R_{wp'}$ = 0.1973 (Figure S26). [**(1):** *a* = 11.839 (2) Å, *b* = 6.788 (2) Å, *c* = 14.639 (6) Å, *V* = 1176.4 (6) Å^3; **(8):** *a* = 10.1441 (13) Å, *b* = 16.250 (2) Å, *c* = 23.6481 (14) Å, β = 99.305 (10) °, *V* = 3846.9 (7) Å^3].

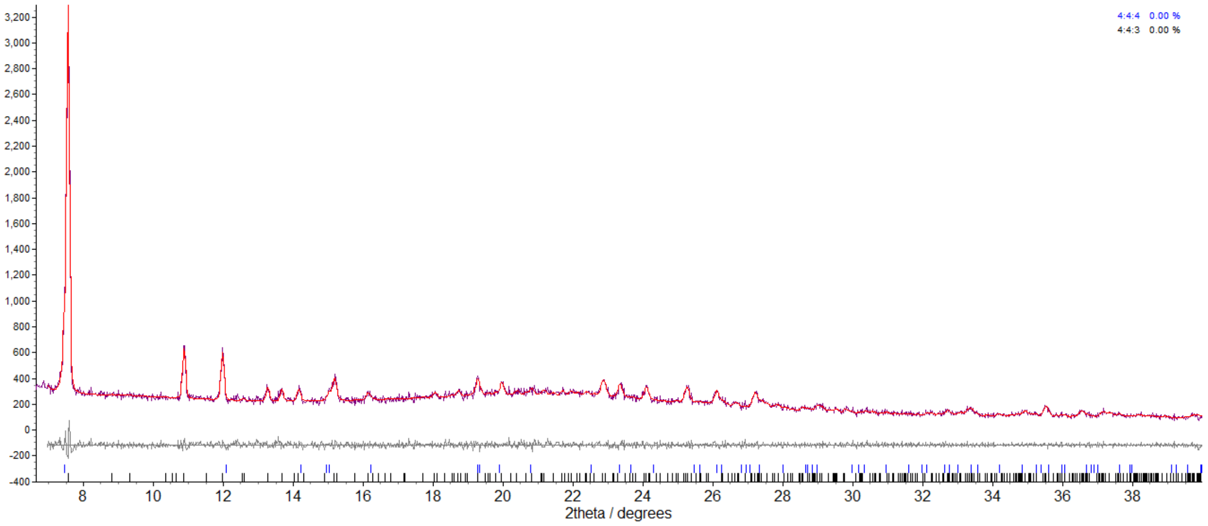

Figure S26. Observed (purple) and calculated (red) profiles and difference plot [I_{obs}-I_{calc}] (grey) of the Pawley refinement. (2θ range 7 - 40 °, d_{min} = 2.25 Å). Blue and black tickmarks correspond to reflections for **1** and **8**, respectively.

After 4 hours 45 min at 433 K.

The sample was kept at 433 K for a further one hour and 15 minutes. The pattern was recorded again. Visual inspection and comparison of the data indicated the presence of both **1** and **8**. The unit cells of both phases from the previous refinement were used as the starting point for a mixed-phase Pawley refinement, employing 452 parameters (8 background, 1 zero error, 9 profile, 7 cell, 427 reflections), resulting in final indices of fit R_{wp} = 0.0695, $R_{wp'}$ = 0.1772 (Figure S27). [**(1)** *a* = 11.830 (2) Å, *b* = 6.7924 (14) Å, *c* = 14.644 (3) Å, *V* = 1176.7 (4) Å^3; **(8):** *a* = 10.1557 (12) Å, *b* = 16.249 (2) Å, *c* = 23.6418 (14) Å, β = 99.293 (9) °, *V* = 3850.2 (7) Å^3].

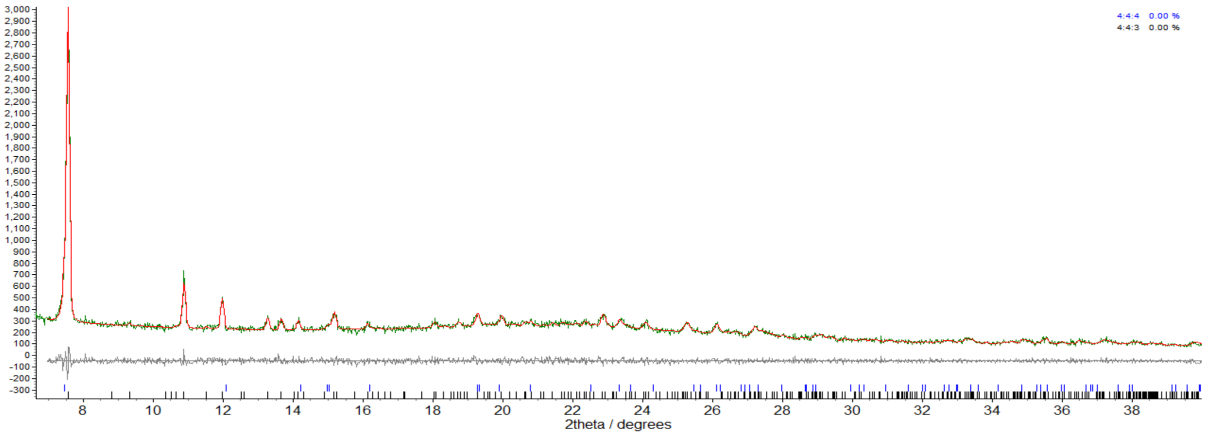

Figure S27. Observed (green) and calculated (red) profiles and difference plot [I_{obs}-I_{calc}] (grey) of the Pawley refinement. (2θ range 7 - 40 °, d_{min} = 2.25 Å). Blue and black tickmarks correspond to reflections for **1** and **8**, respectively.

After 6 hours at 433 K.

The sample was kept at 433 K for a further one hour and 15 minutes. The pattern was recorded again. Visual inspection and comparison of the data indicated the presence of both **1** and **8**. The unit cells of both phases from the previous refinement were used as the starting point for a mixed-phase Pawley refinement, employing 452 parameters (8 background, 1 zero error, 9 profile, 7 cell, 427 reflections), resulting in final indices of fit R_{wp} = 0.0704, $R_{wp'}$ = 0.2050 (Figure S28). [**(1):** *a* = 11.710 (2) Å, *b* = 6.7973 (2) Å, *c* = 14.676 (6) Å, *V* = 1178.1 (7) Å^3; **(8):** *a* = 10.186 (2) Å, *b* = 16.256 (2) Å, *c* = 23.619 (2) Å, β = 99.174 (10) °, *V* = 3861.1 (9) Å^3].

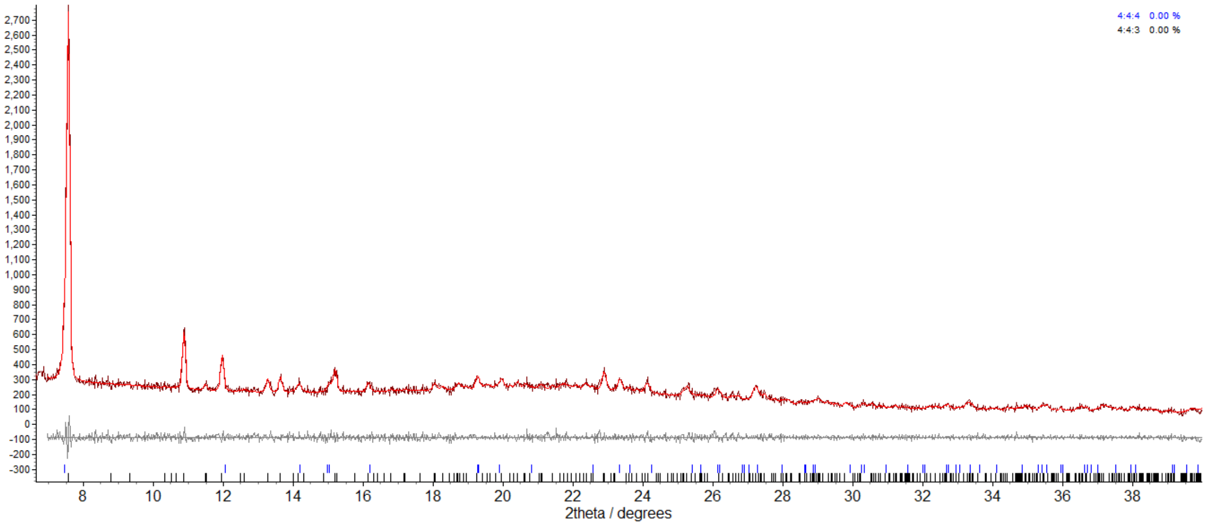

Figure S28. Observed (brown) and calculated (red) profiles and difference plot [I_{obs}-I_{calc}] (grey) of the Pawley refinement. (2θ range 7 - 40 °, d_{min} = 2.25 Å). Blue and black tickmarks correspond to reflections for **1** and **8**, respectively.

After 7 hours 15 min at 433 K.

The sample was kept at 433 K for a further one hour and 15 minutes. The pattern was recorded again. Visual inspection and comparison of the data indicated the presence of both **1** and **8**. The unit cells of both phases from the previous refinement were used as the starting point for a mixed-phase Pawley refinement, employing 452 parameters (8 background, 1 zero error, 9 profile, 7 cell, 427 reflections), resulting in final indices of fit R_{wp} = 0.0690, $R_{wp'}$ = 0.2258 (Figure S29). [**(1):** *a* = 11.821 (3) Å, *b* = 6.795 (3) Å, *c* = 14.672 (7) Å, *V* = 1178.5 (8) Å^3; **(8):** *a* = 10.172 (2) Å, *b* = 16.265 (2) Å, *c* = 23.644 (2) Å, β = 99.219 (12) °, *V* = 3861.2 (9) Å^3].

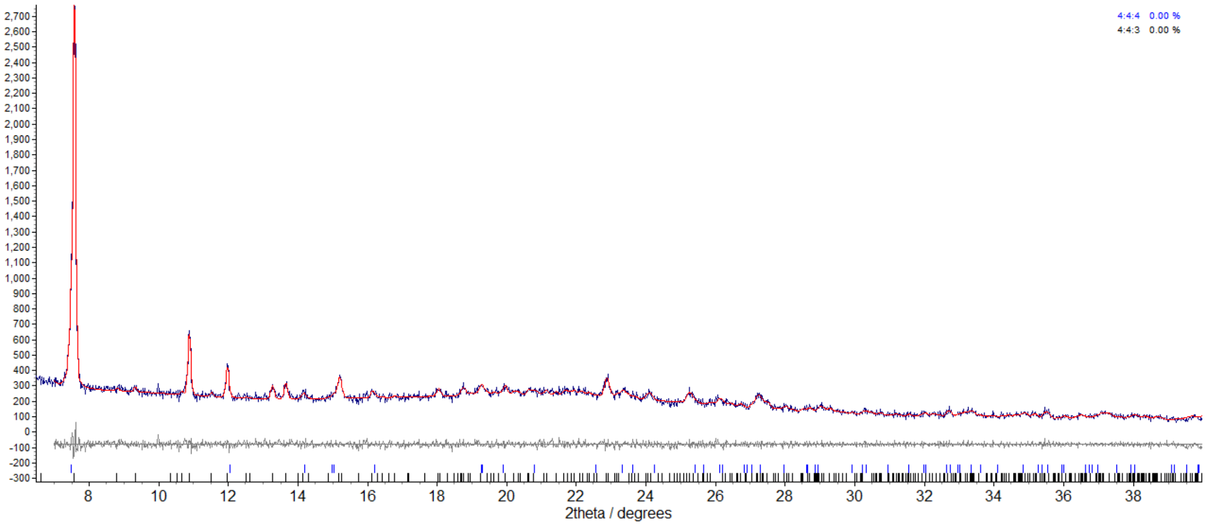

Figure S29. Observed (blue) and calculated (red) profiles and difference plot [I_{obs}-I_{calc}] (grey) of the Pawley refinement. (2θ range 7 - 40 °, d_{min} = 2.25 Å). Blue and black tickmarks correspond to reflections for **1** and **8**, respectively.

After 8 hours 30 min at 433 K

The sample was kept at 433 K for another 8 hours and 30 mins. The pattern was recorded again. Visual inspection of the data suggested that the only remaining phase was **8**. The unit cell of the 4:4:3 phase from the previous refinement was used as the starting point for a Pawley refinement, employing 386 parameters (8 background, 1 zero error, 5 profile, 4 cell, 368 reflections), resulting in final indices of fit R_{wp} = 0.0801, $R_{wp'}$ = 0.286 (Figure S30). [**(8):** *a* = 10.170(3) Å, *b* = 16.255(2) Å, *c* = 23.643(2) Å, β = 99.23(2) °, *V* = 3857.9(11) Å^3].

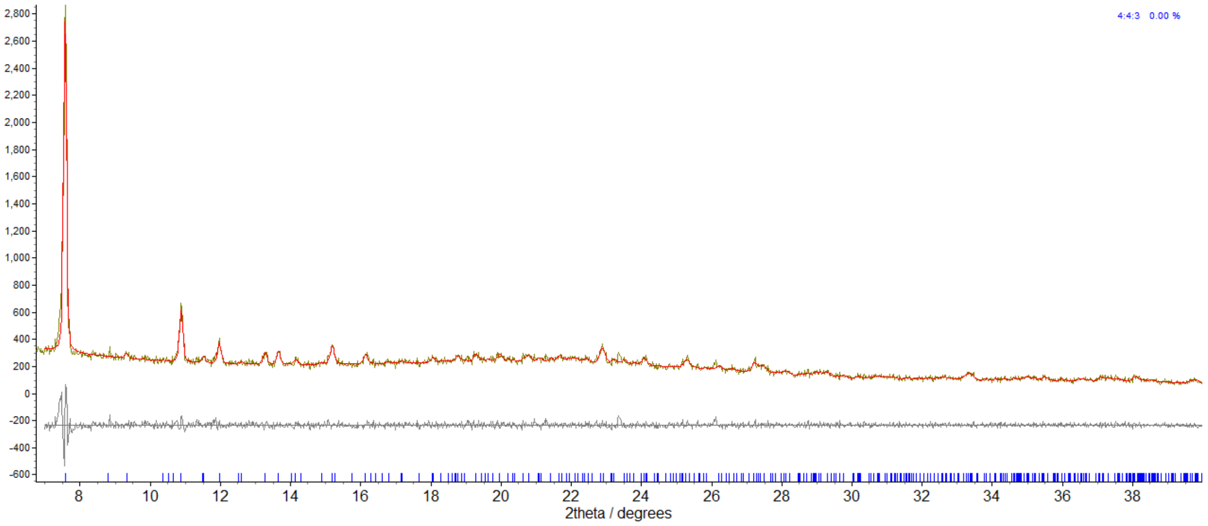

Figure S30. Observed (gold) and calculated (red) profiles and difference plot [I_{obs}-I_{calc}] (grey) of the Pawley refinement. (2θ range 6 - 40°, d_{min} = 2.25 Å).

After 9 hours 45 min at 433 K.

The sample was kept at 433 K for a further one hour and 15 minutes. The pattern was recorded again. The unit cell of the 4:4:3 phase (**8**) from the previous refinement were used as the starting point for a Pawley refinement, employing 386 parameters (8 background, 1 zero error, 5 profile, 4 cell, 368

reflections), resulting in final indices of fit R_{wp} = 0.0766, $R_{wp'}$ = 0.2774 (Figure S31). [**(8):** *a* = 10.174 (2) Å, *b* = 16.255 (2) Å, *c* = 23.632 (2) Å, β = 99.23 (2) °, *V* = 3857.6 (11) $Å^3$].

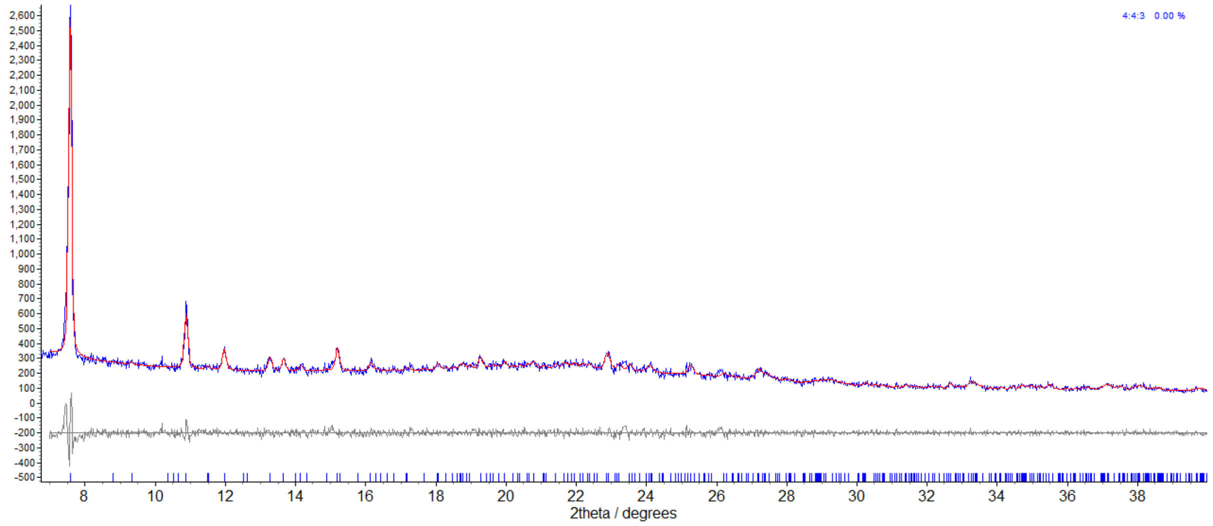

Figure S31. Observed (blue) and calculated (red) profiles and difference plot [I_{obs}-I_{calc}] (grey) of the Pawley refinement. (2θ range 7 - 40 °, d_{min} = 2.25 Å).

After 11 hours at 433 K.

The sample was kept at 433 K for a further one hour and 15 minutes. The pattern was recorded again. The unit cell of the 4:4:3 phase (**8**) from the previous refinement were used as the starting point for a Pawley refinement, employing 386 parameters (8 background, 1 zero error, 5 profile, 4 cell, 368 reflections), resulting in final indices of fit R_{wp} = 0.0774, $R_{wp'}$ = 0.2872 (Figure S32). [**(8):** *a* = 10.174 (3) Å, *b* = 16.255 (3) Å, *c* = 23.634 (2) Å, β = 99.24 (2) °, V = 3857.7 (12) $Å^3$].

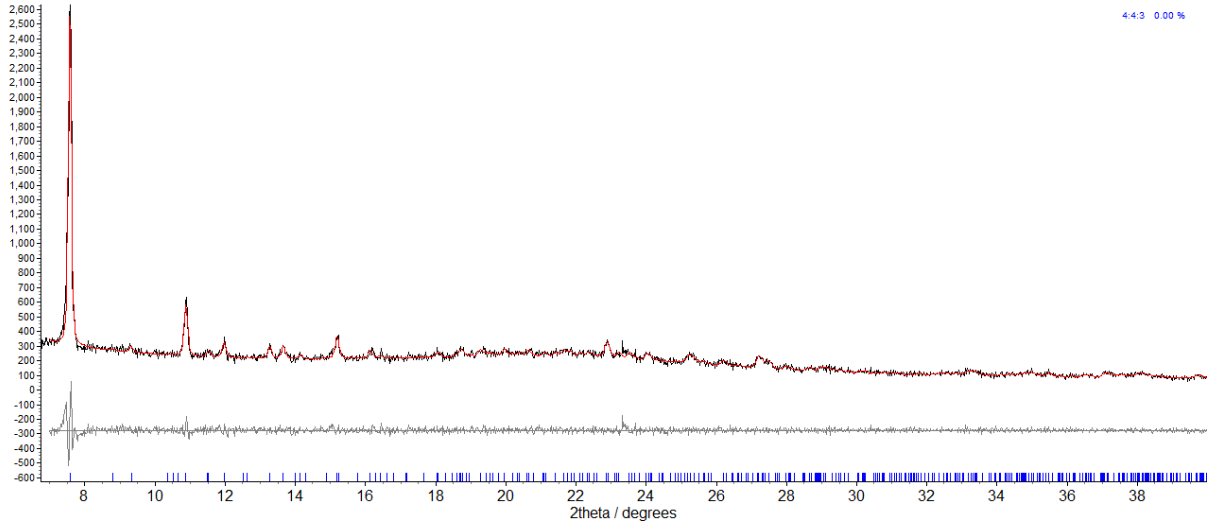

Figure S32. Observed (black) and calculated (red) profiles and difference plot [I_{obs}-I_{calc}] (grey) of the Pawley refinement. (2θ range 7 - 40 °, d_{min} = 2.25 Å).

After 12 hours 15 min at 433 K.

The sample was kept at 433 K for a further one hour and 15 minutes. The pattern was recorded again. The unit cell of the 4:4:3 phase (**8**) from the previous refinement were used as the starting point for a Pawley refinement, employing 386 parameters (8 background, 1 zero error, 5 profile, 4 cell, 368 reflections), resulting in final indices of fit R_{wp} = 0.0804, $R_{wp'}$ = 0.2828 (Figure S33). [**(8):** *a* = 10.176 (3) Å, *b* = 16.255 (2) Å, *c* = 23.628 (2) Å, β = 99.24 (2) °, *V* = 3857.7 (13) Å^3].

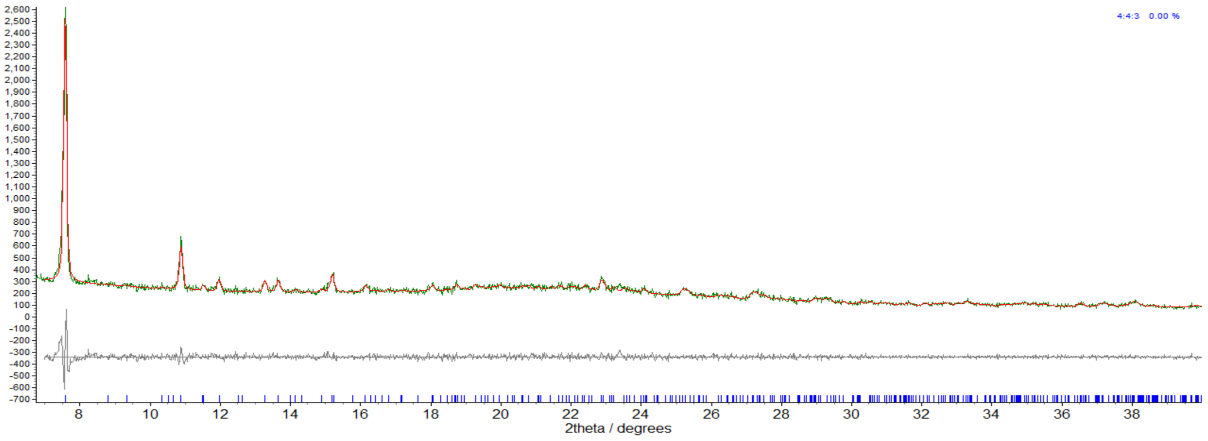

Figure S33. Observed (green) and calculated (red) profiles and difference plot [I_{obs}-I_{calc}] (grey) of the Pawley refinement. (2θ range 7 - 40 °, d_{min} = 2.25 Å).

After 13 hours 30 min at 433 K.

The sample was kept at 433 K for a further one hour and 15 minutes. The pattern was recorded again. The unit cell of the 4:4:3 phase (**8**) from the previous refinement were used as the starting point for a Pawley refinement, employing 386 parameters (8 background, 1 zero error, 5 profile, 4 cell, 368 reflections), resulting in final indices of fit R_{wp} = 0.0803, $R_{wp'}$ = 0.2970 (Figure S34). [**(8):** *a* = 10.174 (3) Å, *b* = 16.252 (3) Å, *c* = 23.621 (2) Å, β = 99.24 (2) °, *V* = 3855.1 (12) Å^3].

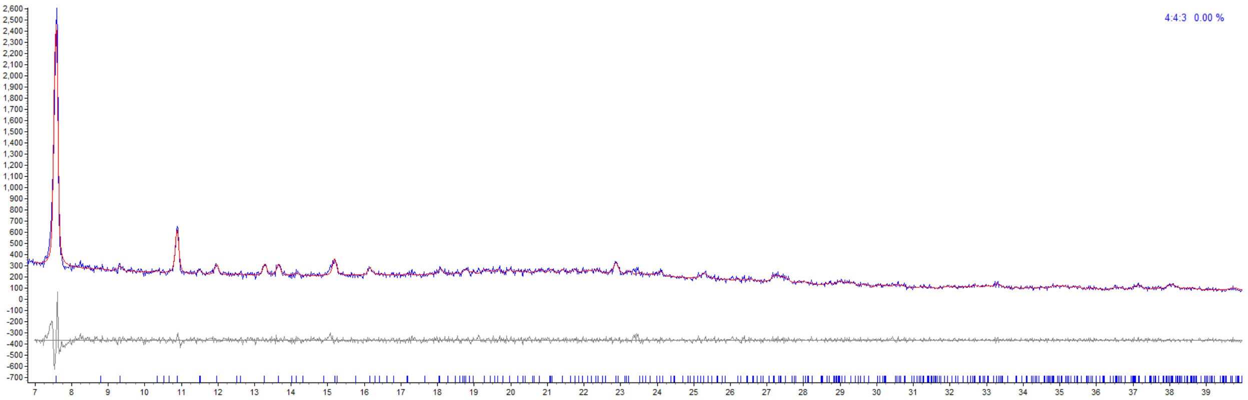

Figure S34. Observed (blue) and calculated (red) profiles and difference plot [I_{obs}-I_{calc}] (grey) of the Pawley refinement. (2θ range 7 - 40 °, d_{min} = 2.25 Å).

After 14 hours 45 min at 433 K.

The sample was kept at 433 K for a further one hour and 15 minutes. The pattern was recorded again. The unit cell of the 4:4:3 phase (**8**) from the previous refinement were used as the starting point for a Pawley refinement, employing 390 parameters (12 background, 1 zero error, 5 profile, 4 cell, 368 reflections), resulting in final indices of fit R_{wp} = 0.0735, $R_{wp'}$ = 0.3057 (Figure S35). [**(8):** *a* = 10.169 (4) Å, *b* = 16.248 (3) Å, *c* = 23.605 (3) Å, β = 99.16 (4) °, *V* = 3850 (2) Å^3].

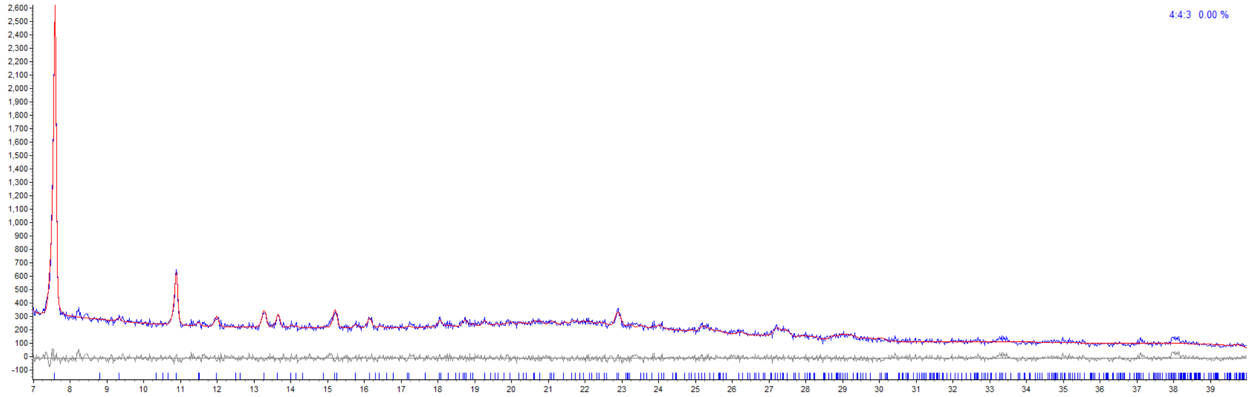

Figure S35. Observed (blue) and calculated (red) profiles and difference plot [I_{obs}-I_{calc}] (grey) of the Pawley refinement. (2θ range 7 - 40 °, d_{min} = 2.25 Å).

After 16 hours at 433 K.

The sample was kept at 433 K for a further one hour and 15 minutes. The unit cell of the 4:4:3 phase (**8**) from the previous refinement were used as the starting point for a Pawley refinement, employing 390 parameters (12 background, 1 zero error, 5 profile, 4 cell, 368 reflections), resulting in final indices of fit R_{wp} = 0.0725, $R_{wp'}$ = 0.2991 (Figure S36). [**(8):** *a* = 10.157 (4) Å, *b* = 16.240 (3) Å, *c* = 23.581 (3) Å, β = 99.24 (4) °, *V* = 3839 (2) Å^3].

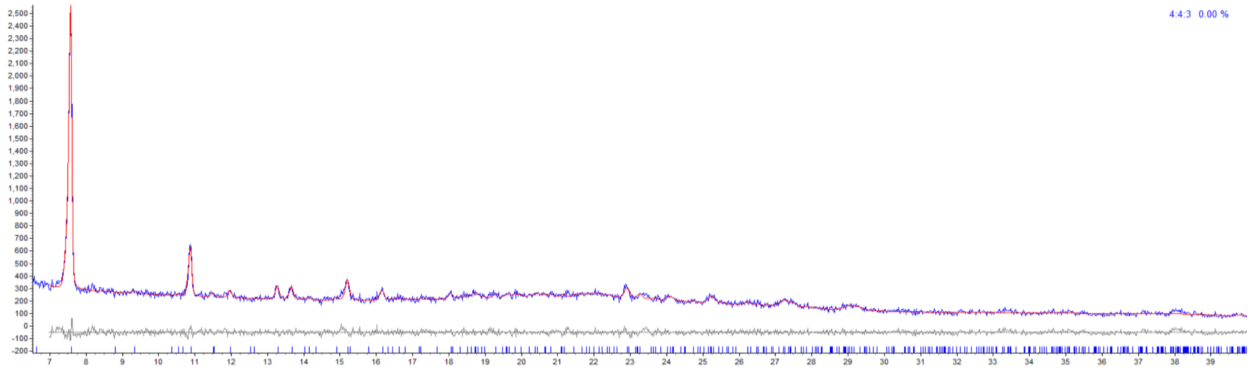

Figure S36. Observed (blue) and calculated (red) profiles and difference plot [I_{obs}-I_{calc}] (grey) of the Pawley refinement. (2θ range 7 - 40 °, d_{min} = 2.25 Å).

After 17 hours 15 min at 433 K.

The sample was kept at 433 K for a further one hour and 15 minutes. The pattern was recorded again. The appearance of a new peak at 2θ 8.25° was also observed, which did not correspond to either of the known phases, but was insufficient in intensity to further characterise. The unit cell of the 4:4:3 phase (**8**) from the previous refinement were used as the starting point for a Pawley refinement, employing 390 parameters (12 background, 1 zero error, 5 profile, 4 cell, 368 reflections), resulting in final indices of fit R_{wp} = 0.0674, $R_{wp'}$ = 0.2806 (Figure S37). [(**8**): *a* = 10.164 (4) Å, *b* = 16.239 (3) Å, *c* = 23.582 (3) Å, β = 99.27 (4) °, *V* = 3841 (2) $Å^3$].

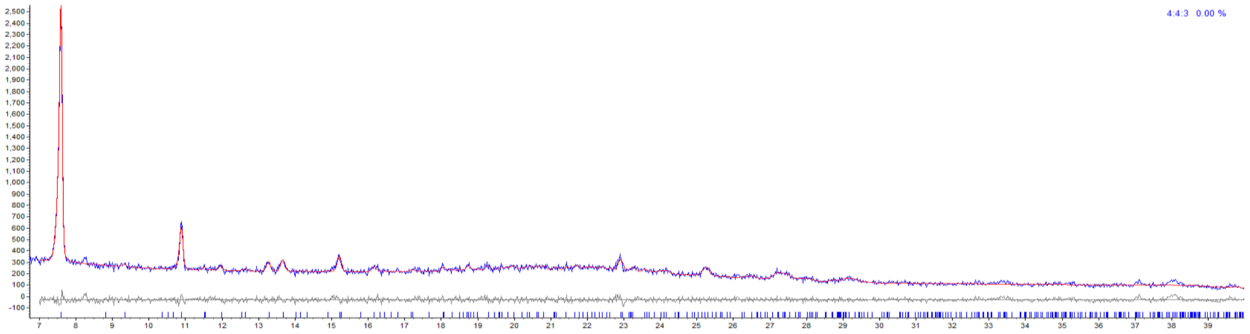

Figure S37. Observed (blue) and calculated (red) profiles and difference plot [I_{obs}-I_{calc}] (grey) of the Pawley refinement. (2θ range 7 - 40 °, d_{min} = 2.25 Å).

After returning to 298 K

The sample was cooled back to 298 K and held for two hours, before recording the pattern again, at a longer exposure. The peak corresponding to the unknown phase at 2θ = 8.25° remained present, in addition to the appearance of a smaller peak at 2θ = 8.55°. This region was therefore excluded from the refinement. The pattern was indexed, and a unit cell resembling that of **8** obtained from single-crystal X-ray data at 150 K was identified. This unit cell was used as the starting point for a Pawley refinement, employing 386 parameters (8 background, 1 zero error, 5 profile, 4 cell, 368 reflections), resulting in final indices of fit R_{wp} = 0.0361, $R_{wp'}$ = 0.140 (Figure S38). [(**8**): *a* = 10.0347(12) Å, *b* = 16.2603(11) Å, *c* = 23.4602(9) Å, β = 99.150(8) °, *V* = 3779.2(5) $Å^3$].

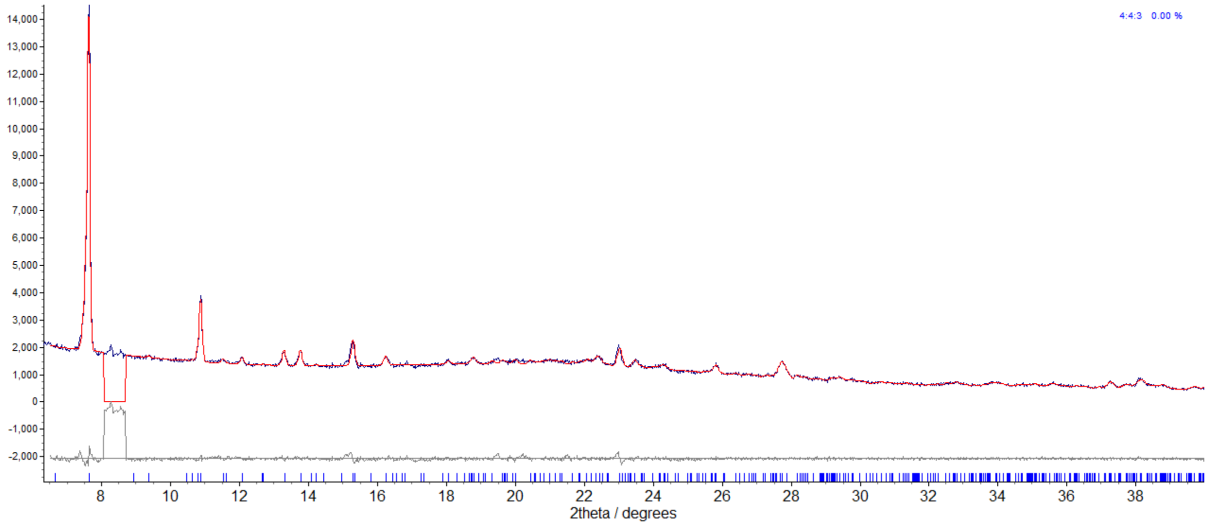

Figure S38. Observed (blue) and calculated (red) profiles and difference plot [I_{obs}-I_{calc}] (grey) of the Pawley refinement. (2θ range 7 - 40°, d_{min} = 2.25 Å).

$[Ag_4(O_2C(CF_2)_2CF_3)_4(quin)_4]$ (3) → $[Ag_4(O_2C(CF_2)_2CF_3)_4(quin)_3]$ (10):

A stack plot of the series of PXRD patterns is shown in Figure 7b in the main paper for the study of the conversion of **3** to **10**. Patterns at 298 K were measured in the range $3 \leq 2\theta \leq 40°$ using a step size of 0.015° and step time of 1.0 s, giving a total exposure time of 41 mins. No scans were collected during heating to 433 K. Patterns at 433 K were measured in the range $3 \leq 2\theta \leq 40°$ using a step size of 0.015° and step time of 0.5 s, giving a total exposure time of 21 mins. The patterns were compared to the calculated PXRD patterns from the single-crystal structures of **3** and **10** and fitted using Pawley refinement.

Initial pattern at 298 K

The pattern was indexed and a unit cell resembling that of **3** obtained from single-crystal X-ray data at 150 K was identified. This unit cell was used as the starting point for a Pawley refinement, employing 154 parameters (13 background, 1 zero error, 5 profile, 4 cell, 131 reflections), resulting in final indices of fit R_{wp} = 0.0610, $R_{wp'}$ = 0.148 (Figure S39). [**(3):** a = 14.6269(11) Å, b = 6.7864(6) Å, c = 14.3807(9) Å, β = 96.82(1) °, V = 1417.4(2) Å^3].

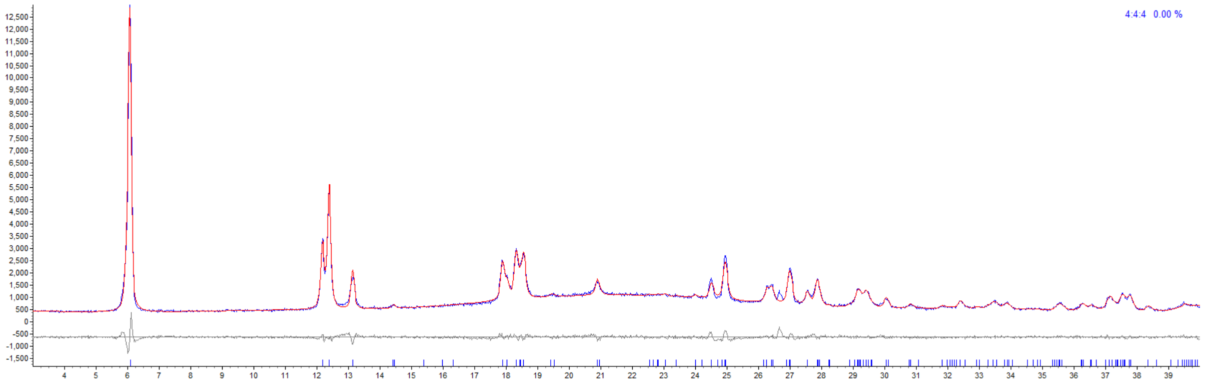

Figure S39. Observed (blue) and calculated (red) profiles and difference plot [I_{obs}-I_{calc}] (grey) of the Rietveld refinement. (2θ range 3 - 40°, d_{min} = 2.25 Å).

After 1 hour at 433 K

The sample was heated to 433 K and allowed to stay at this temperature for a period of one hour. The pattern was recorded again. Visual inspection and comparison of the data indicated the presence of **3** and **10**. The appearance of a new peak at 2θ = 6.69° was also observed, which did not correspond to either of these two phases, and thus was excluded from the refinement. The unit cell of **3** from the refinement at 298 K and that of **10** from a later point in the study at 433 K (when it is the only phase present) were used as the starting point for a mixed-phase Pawley refinement, employing 648 parameters (12 background, 1 zero error, 9 profile, 8 cell, 618 reflections), resulting in final indices of fit R_{wp} = 0.0494, $R_{wp'}$ = 0.239 (Figure S40). [**(3):** a = 14.7853(9) Å, b = 6.7542(9) Å, c = 14.2912(8) Å, β = 96.75(1) °, V = 1417.3(2) Å^3; **(10):** a = 11.2818(9) Å, b = 16.1521(9) Å, c = 28.2213(14) Å, β = 92.779(6) °, V = 5136.6(5) Å^3].

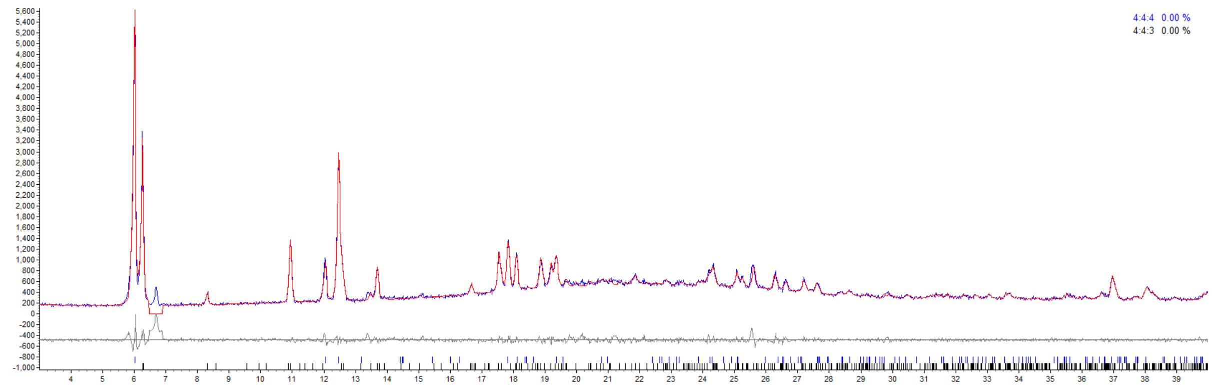

Figure S40. Observed (blue) and calculated (red) profiles and difference plot [I_{obs}-I_{calc}] (grey) of the Pawley refinement. (2θ range 3 - 40°, d_{min} = 2.25 Å). Blue and black tickmarks correspond to reflections for **3** and **10**, respectively.

After 2 hours at 433 K.

The sample was kept at 433 K for a further hour and the pattern was recorded again. The peak corresponding to some unknown phase at 2θ 6.69 ° remained and was excluded from refinement. The unit cells of both the 4:4:4 phase (**3**) and the 4:4:3 phase (**10**) from the previous refinement were used as the starting point for a mixed-phase Pawley refinement, employing 648 parameters (14 background, 1 zero error, 9 profile, 8 cell, 616 reflections), resulting in final indices of fit R_{wp} = 0.0840, $R_{wp'}$ = 0.1874 (Figure S41). [**(3):** *a* = 14.7829 (11) Å, *b* = 6.7564 (11) Å, *c* = 14.2888 (11) Å, β = 96.722 (11) °, *V* = 1417.3 (3) Å^3; **(10):** *a* = 11.2824 (9) Å, *b* = 16.1524 (11) Å, *c* = 28.222 (16) Å, β = 92.754 (7) °, *V* = 5137.1 (6) Å^3].

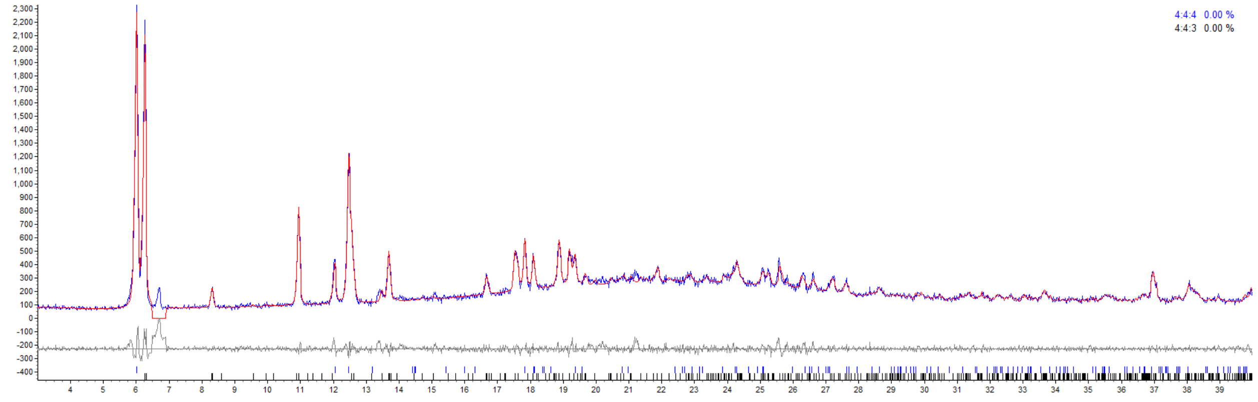

Figure S41. Observed (blue) and calculated (red) profiles and difference plot [I_{obs}-I_{calc}] (grey) of the Pawley refinement. (2θ range 3 - 40 °, d_{min} = 2.25 Å). Blue and black tickmarks correspond to reflections for **3** and **10**, respectively.

After 2 hours 45 mins at 433 K.

The sample was kept at 433 K for a further 45 minutes and the pattern was recorded again. The peak corresponding to some unknown phase at 2θ 6.69° remained and was excluded from refinement. The unit cells of both the 4:4:4 phase (**3**) and the 4:4:3 phase (**10**) from the previous refinement were used

as the starting point for a mixed-phase Pawley refinement, employing 649 parameters (14 background, 1 zero error, 9 profile, 8 cell, 617 reflections), resulting in final indices of fit R_{wp} = 0.0823, $R_{wp'}$ = 0.1791 (Figure S42). [**(3):** *a* = 14.7827 (13) Å, *b* = 6.7552 (9) Å, *c* = 14.2892 (12) Å, β = 96.73 (14) °, *V* = 1417.1 (3) Å^3; **(10):** *a* = 11.2822 (8) Å, *b* = 16.1506 (9) Å, *c* = 28.2204 (15) Å, β = 92.757 (6) °, *V* = 5136.2 (6) Å^3].

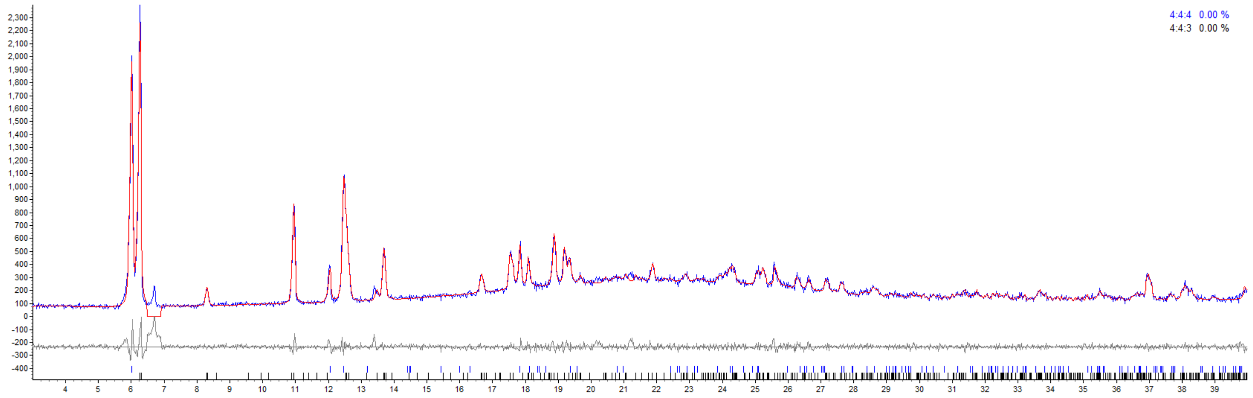

Figure S42. Observed (blue) and calculated (red) profiles and difference plot [I_{obs}-I_{calc}] (grey) of the Pawley refinement. (2θ range 5 - 40 °, d_{min} = 2.25 Å). Blue and black tickmarks correspond to reflections for **3** and **10**, respectively.

After 3 hours 30 mins at 433 K.

The sample was kept at 433 K for a further 45 minutes and the pattern was recorded again. The peak corresponding to some unknown phase at 2θ 6.69° remained and was excluded from refinement. The unit cells of both the 4:4:4 phase (**3**) and the 4:4:3 phase (**10**) from the previous refinement were used as the starting point for a mixed-phase Pawley refinement, employing 649 parameters (14 background, 1 zero error, 9 profile, 8 cell, 617 reflections), resulting in final indices of fit R_{wp} = 0.0803, $R_{wp'}$ = 0.1812 (Figure S43). [**(3):** *a* = 14.7788 (13) Å, *b* = 6.7540 (11) Å, *c* = 14.2870 (13) Å, β = 96.665 (14) °, *V* = 1416.4 (3) Å^3; **(10):** *a* = 11.2810 (10) Å, *b* = 16.1480 (10) Å, *c* = 28.2127 (14) Å, β = 92.717 (7) °, *V* = 5133.6 (6) Å^3].

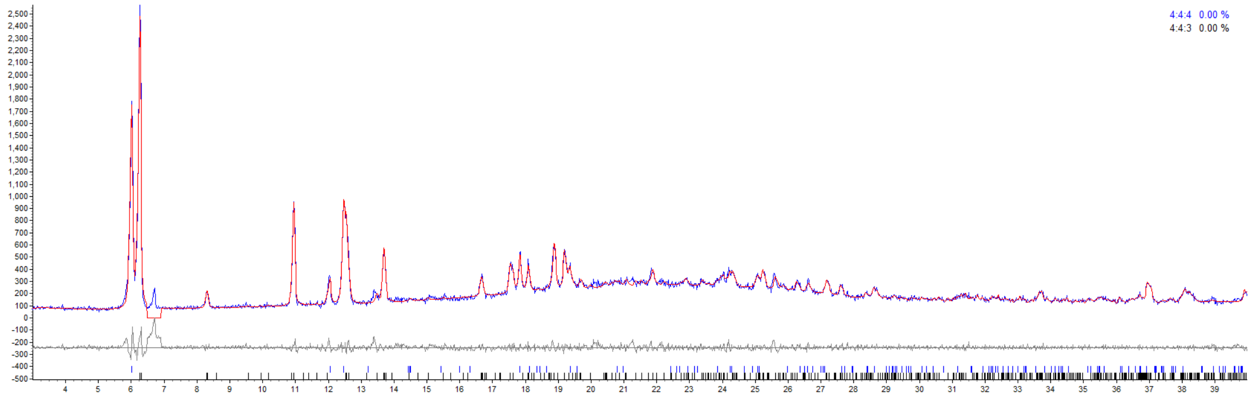

Figure S43. Observed (blue) and calculated (red) profiles and difference plot [I_{obs}-I_{calc}] (grey) of the Pawley refinement. (2θ range 5 - 40 °, d_{min} = 2.25 Å). Blue and black tickmarks correspond to reflections for **3** and **10**, respectively.

After 4 hours 15 mins at 433 K.

The sample was kept at 433 K for a further 45 minutes and the pattern was recorded again. The peak corresponding to some unknown phase at 2θ 6.69° remained and was excluded from refinement. The unit cells of both the 4:4:4 phase (**3**) and the 4:4:3 phase (**10**) from the previous refinement were used as the starting point for a mixed-phase Pawley refinement, employing 649 parameters (14 background, 1 zero error, 9 profile, 8 cell, 617 reflections), resulting in final indices of fit R_{wp} = 0.0823, $R_{wp'}$ = 0.1806 (Figure S44). [**(3):** *a* = 14.7787 (14) Å, *b* = 6.7534 (10) Å, *c* = 14.2894 (14) Å, β = 96.668 (14) °, *V* = 1416.5 (3) Å^3; **(10):** *a* = 11.2775 (9) Å, *b* = 16.1523 (9) Å, *c* = 28.2088 (14) Å, β = 92.697 (7) °, *V* = 5133.6 (7) Å^3].

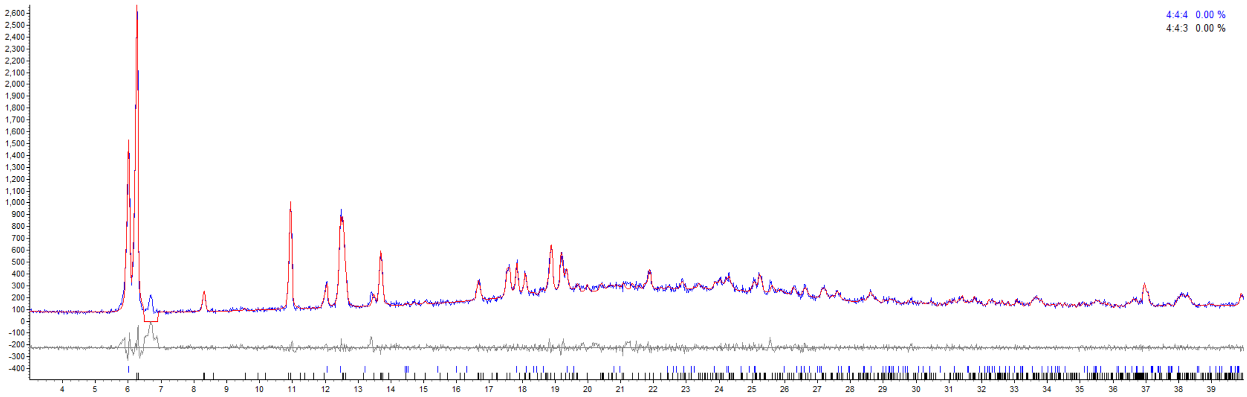

Figure S44. Observed (blue) and calculated (red) profiles and difference plot [I_{obs}-I_{calc}] (grey) of the Pawley refinement. (2θ range 3 - 40 °, d_{min} = 2.25 Å). Blue and black tickmarks correspond to reflections for **3** and **10**, respectively.

After 5 hours at 433 K.

The sample was kept at 433 K for a further 45 minutes and the pattern was recorded again. The peak corresponding to some unknown phase at 2θ 6.69° remained and was excluded from refinement. The unit cells of both the 4:4:4 phase (**3**) and the 4:4:3 phase (**10**) from the previous refinement were used as the starting point for a mixed-phase Pawley refinement, employing 649 parameters (14 background, 1 zero error, 9 profile, 8 cell, 617 reflections), resulting in final indices of fit R_{wp} = 0.0794, $R_{wp'}$ = 0.1760 (Figure S45). [**(3):** *a* = 14.7802 (14) Å, *b* = 6.7543 (12) Å, *c* = 14.2891 (14) Å, β = 96.656 (13) °, *V* = 1416.9 (3) Å^3; **(10):** *a* = 11.2783 (8) Å, *b* = 16.1494 (9) Å, *c* = 28.2138 (14) Å, β = 92.709 (7) °, *V* = 5133.1 (5) Å^3].

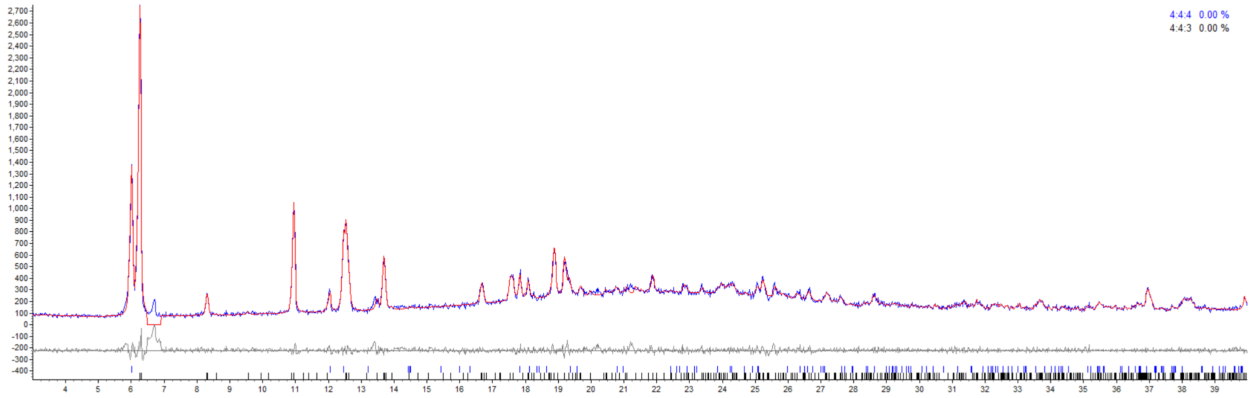

Figure S45. Observed (blue) and calculated (red) profiles and difference plot [I_{obs}-I_{calc}] (grey) of the Pawley refinement. (2θ range 3 - 40 °, d_{min} = 2.25 Å). Blue and black tickmarks correspond to reflections for **3** and **10**, respectively.

After 5 hours 45 mins at 433 K.

The sample was kept at 433 K for a further 45 minutes and the pattern was recorded again. The peak corresponding to some unknown phase at 2θ 6.69° remained and was excluded from refinement. The unit cells of both the 4:4:4 phase (**3**) and the 4:4:3 phase (**10**) from the previous refinement were used as the starting point for a mixed-phase Pawley refinement, employing 649 parameters (14 background, 1 zero error, 9 profile, 8 cell, 617 reflections), resulting in final indices of fit R_{wp} = 0.0805, $R_{wp'}$ = 0.1775 (Figure 46). [(**3**): *a* = 14.7796 (15) Å, *b* = 6.7519 (11) Å, *c* = 14.2826 (14) Å, β = 96.605 (13) °, *V* = 1415.8 (3) Å^3; **(10):** a = 11.2755 (9) Å, *b* = 16.1485 (9) Å, *c* = 28.2066 (14) Å, β = 92.688 (7) °, *V* = 5130.3 (5) Å^3].

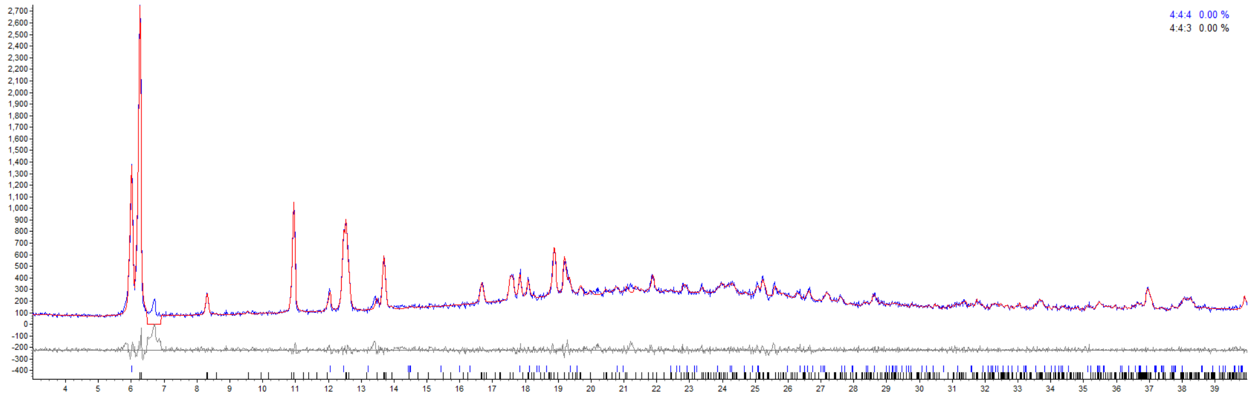

Figure S46. Observed (blue) and calculated (red) profiles and difference plot [I_{obs}-I_{calc}] (grey) of the Pawley refinement. (2θ range 3 - 40 °, d_{min} = 2.25 Å). Blue and black tickmarks correspond to reflections for **3** and **10**, respectively.

After 6 hours 30 mins at 433 K.

The sample was kept at 433 K for a further 45 minutes and the pattern was recorded again. The peak corresponding to some unknown phase at 2θ 6.69° remained and was excluded from refinement. The unit cells of both the 4:4:4 phase (**3**) and the 4:4:3 phase (**10**) from the previous refinement were used as the starting point for a mixed-phase Pawley refinement, employing 649 parameters (14

background, 1 zero error, 9 profile, 8 cell, 617 reflections), resulting in final indices of fit R_{wp} = 0.0821, $R_{wp'}$ = 0.1771 (Figure S47). [[**3**]: *a* = 14.781 (2) Å, *b* = 6.7508 (13) Å, *c* = 14.287 (2) Å, β = 96.660 (14) °, *V* = 1416.0 (4) Å^3; **[10]:** *a* **=** 11.27679 (9) Å, *b* = 16.1485 (9) Å, *c* = 28.2049 (13) Å, β = 92.694 (6) °, *V* = 5130.6 (5) Å^3].

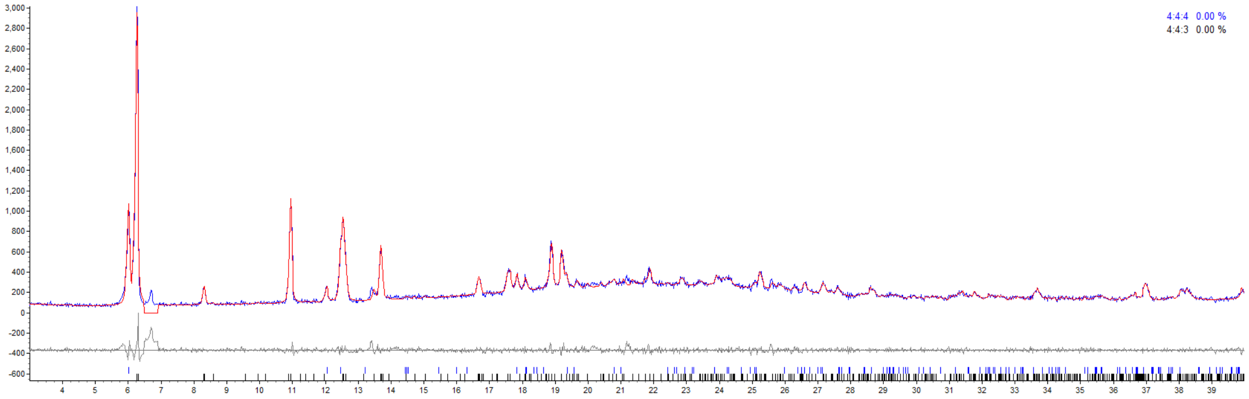

Figure S47. Observed (blue) and calculated (red) profiles and difference plot [I_{obs}-I_{calc}] (grey) of the Pawley refinement. (2θ range 3 - 40 °, d_{min} = 2.25 Å). Blue and black tickmarks correspond to reflections for **3** and **10**, respectively.

After 7 hours 15 mins at 433 K.

The sample was kept at 433 K for a further 45 minutes and the pattern was recorded again. The peak corresponding to some unknown phase at 2θ 6.69° remained and was excluded from refinement. The unit cells of both the 4:4:4 phase (**3**) and the 4:4:3 phase (**10**) from the previous refinement were used as the starting point for a mixed-phase Pawley refinement, employing 649 parameters (14 background, 1 zero error, 9 profile, 8 cell, 617 reflections), resulting in final indices of fit R_{wp} = 0.0808, $R_{wp'}$ = 0.1808 (Figure S48). [**(3):** *a* = 14.781 (2) Å, *b* = 6.7511 (12) Å, *c* = 14.287 (2) Å, β = 96.653 (13) °, *V* = 1416.0 (3) Å^3; **(10):** *a* **=** 11.2758 (9) Å, *b* = 16.1497 (9) Å, *c* = 28.2045 (13) Å, β = 92.693 (7) °, *V* = 5130.4 (6) Å^3].

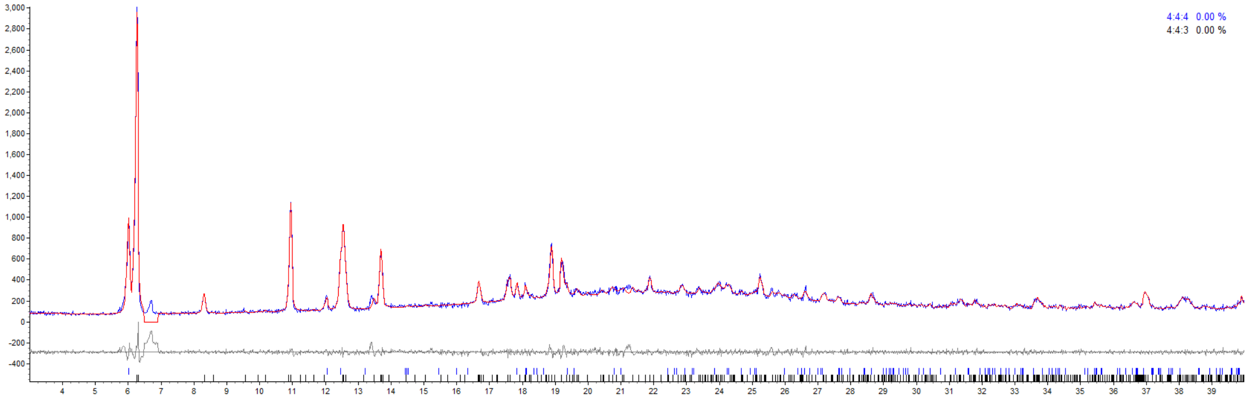

Figure S48. Observed (green) and calculated (red) profiles and difference plot [I_{obs}-I_{calc}] (grey) of the Pawley refinement. (2θ range 3 - 40 °, d_{min} = 2.25 Å). Blue and black tickmarks correspond to reflections for **3** and **10**, respectively.

After 8 hours 45 mins at 433 K.

The sample was kept at 433 K for a further 1 hour and 30 minutes and the pattern was recorded again. The peak corresponding to some unknown phase at 2θ 6.69° remained and was excluded from refinement. The unit cells of both the 4:4:4 phase (**3**) and the 4:4:3 phase (**10**) from the previous refinement were used as the starting point for a mixed-phase Pawley refinement, employing 649 parameters (14 background, 1 zero error, 9 profile, 8 cell, 617 reflections), resulting in final indices of fit R_{wp} = 0.0809, $R_{wp'}$ = 0.1883 (Figure S49). [**(3):** *a* = 14.784 (2) Å, *b* = 6.751 (2) Å, *c* = 14.289 (2) Å, β = 96.65 (2) °, *V* = 1416.6 (4) $Å^3$; **(10):** *a* **=** 11.2761 (9) Å, *b* = 16.1503 (9) Å, *c* = 28.2046 (13) Å, β = 92.691 (6) °, *V* = 5130.7 (6) $Å^3$].

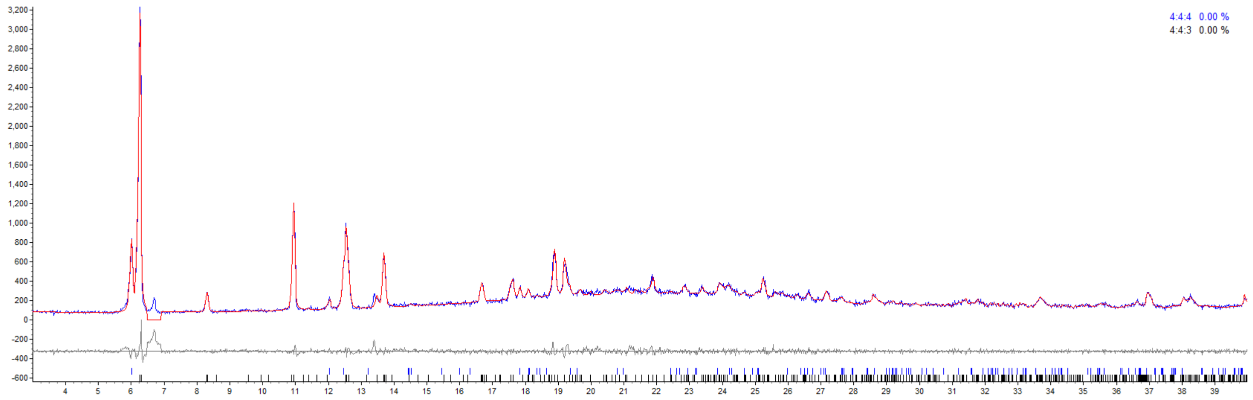

Figure S49. Observed (blue) and calculated (red) profiles and difference plot [I_{obs}-I_{calc}] (grey) of the Pawley refinement. (2θ range 3 - 40 °, d_{min} = 2.25 Å). Blue and black tickmarks correspond to reflections for **3** and **10**, respectively.

After 10 hours 15 mins at 433 K.

The sample was kept at 433 K for a further 1 hour and 30 minutes and the pattern was recorded again. The peak corresponding to some unknown phase at 2θ 6.69° remained and was excluded from refinement. The unit cells of both the 4:4:4 phase (**3**) and the 4:4:3 phase (**10**) from the previous refinement were used as the starting point for a mixed-phase Pawley refinement, employing 649 parameters (14 background, 1 zero error, 9 profile, 8 cell, 617 reflections), resulting in final indices of fit R_{wp} = 0.0830, $R_{wp'}$ = 0.1868 (Figure S50). [**(3):** *a* = 14.782 (2) Å, *b* = 6.7541 (12) Å, *c* = 14.285 (2) Å, β = 96.674 (13) °, *V* = 1416.6 (4) $Å^3$; **(10):** *a* **=** 11.2721 (10) Å, *b* = 16.1498 (9) Å, *c* = 28.2062 (13) Å, β = 92.686 (6) °, *V* = 5129.1 (6) $Å^3$].

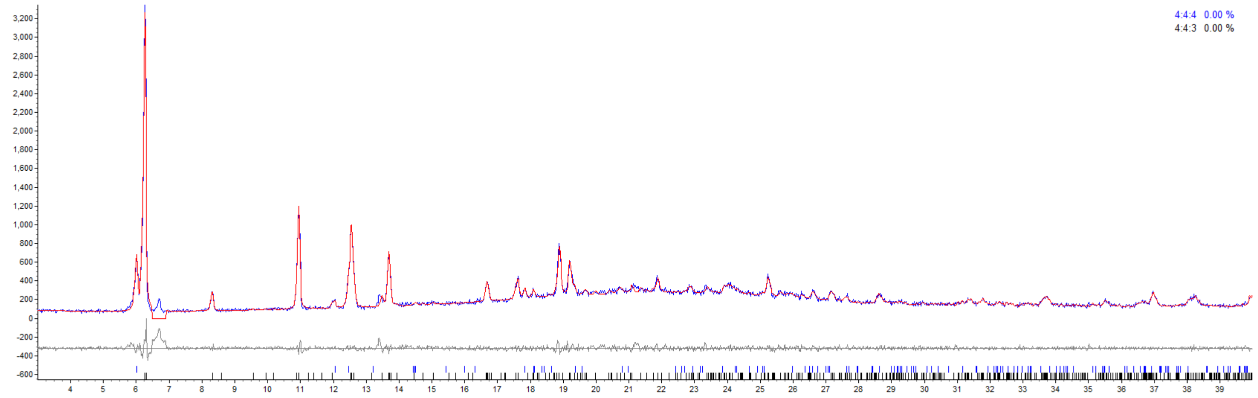

Figure S50. Observed (blue) and calculated (red) profiles and difference plot [I_{obs}-I_{calc}] (grey) of the Pawley refinement. (2θ range 3 - 40 °, d_{min} = 2.25 Å). Blue and black tickmarks correspond to reflections for **3** and **10**, respectively.

After 11 hours 45 mins at 433 K.

The sample was kept at 433 K for a further 1 hour and 30 minutes and the pattern was recorded again. The peak corresponding to some unknown phase at 2θ 6.69° remained and was excluded from refinement. The unit cells of both the 4:4:4 phase (**3**) and the 4:4:3 phase (**10**) from the previous refinement were used as the starting point for a mixed-phase Pawley refinement, employing 649 parameters (14 background, 1 zero error, 9 profile, 8 cell, 617 reflections), resulting in final indices of fit R_{wp} = 0.0810, $R_{wp'}$ = 0.1857 (Figure S51). [**(3):** *a* = 14.778 (3) Å, *b* = 6.756 (2) Å, *c* = 14.299 (2) Å, β = 96.615 (13) °, *V* = 1418.1 (5) $Å^3$; **(10):** *a* **=** 11.2630 (9) Å, *b* = 16.1488 (9) Å, *c* = 28.1975 (13) Å, β = 92.605 (6) °, *V* = 5123.4 (5) $Å^3$].

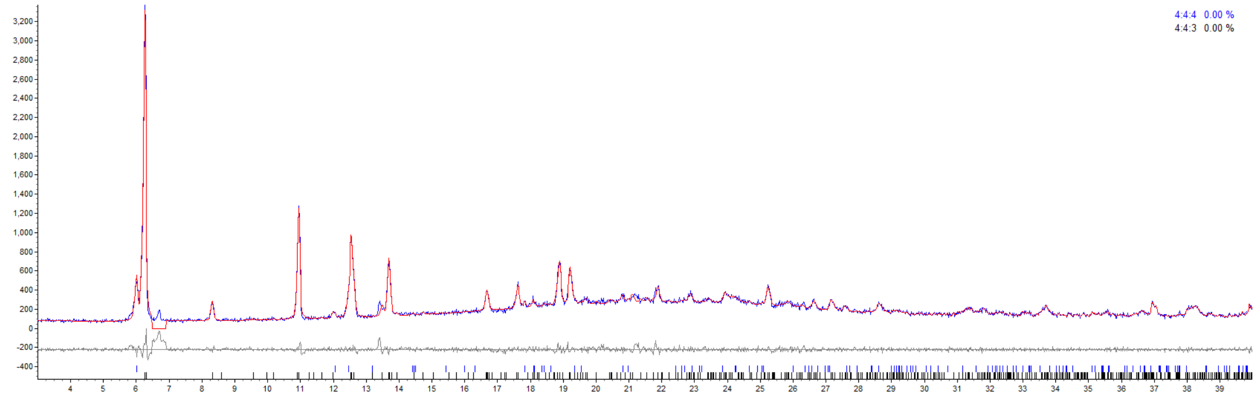

Figure S51. Observed (blue) and calculated (red) profiles and difference plot [I_{obs}-I_{calc}] (grey) of the Pawley refinement. (2θ range 3 - 40 °, d_{min} = 2.25 Å). Blue and black tickmarks correspond to reflections for **3** and **10**, respectively.

After 13 hours 15 mins at 433 K.

The sample was kept at 433 K for a further 1 hour and 30 minutes and the pattern was recorded again. The peak corresponding to some unknown phase at 2θ 6.69° remained and was excluded from refinement. The unit cells of both the 4:4:4 phase (**3**) and the 4:4:3 phase (**10**) from the previous refinement were used as the starting point for a mixed-phase Pawley refinement, employing 649

parameters (14 background, 1 zero error, 9 profile, 8 cell, 617 reflections), resulting in final indices of fit R_{wp} = 0.0821, $R_{wp'}$ = 0.1789 (Figure S52). [**(3):** *a* = 14.782 (2) Å, b = 6.7592 (14) Å, *c* = 14.293 (2) Å, β = 96.589 (12) °, *V* = 1418.7 (4) Å^3; **(10):** *a* **=** 11.2612 (8) Å, b = 16.1490 (9) Å, *c* = 28.1986 (12) Å, β = 92.582 (6) °, *V* = 5122.9 (5) Å^3].

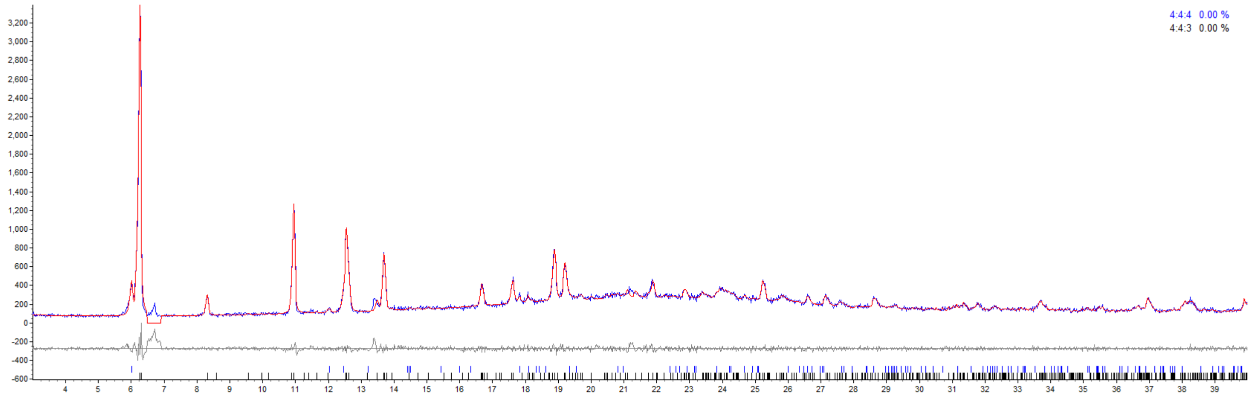

Figure S52. Observed (blue) and calculated (red) profiles and difference plot [I_{obs}-I_{calc}] (grey) of the Pawley refinement. (2θ range 3 - 40 °, d_{min} = 2.25 Å). Blue and black tickmarks correspond to reflections for **3** and **10**, respectively.

After 14 hours 45 mins at 433 K.

The sample was kept at 433 K for a further 1 hour and 30 minutes and the pattern was recorded again. The peak corresponding to some unknown phase at 2θ 6.69° remained and was excluded from refinement. The unit cells of both the 4:4:4 phase (**3**) and the 4:4:3 phase (**10**) from the previous refinement were used as the starting point for a mixed-phase Pawley refinement, employing 649 parameters (14 background, 1 zero error, 9 profile, 8 cell, 617 reflections), resulting in final indices of fit R_{wp} = 0.0824, $R_{wp'}$ = 0.1880 (Figure S53). [**(3):** *a* = 14.767 (3) Å, *b* = 6.7747 (14) Å, *c* = 14.302 (3) Å, β = 96.530 (12) °, *V* = 1421.6 (5) Å^3; **(10):** *a* **=** 11.2621 (9) Å, *b* = 16.1501 (9) Å, *c* = 28.1966 (12) Å, β = 92.527 (6) °, *V* = 5123.5 (6) Å^3].

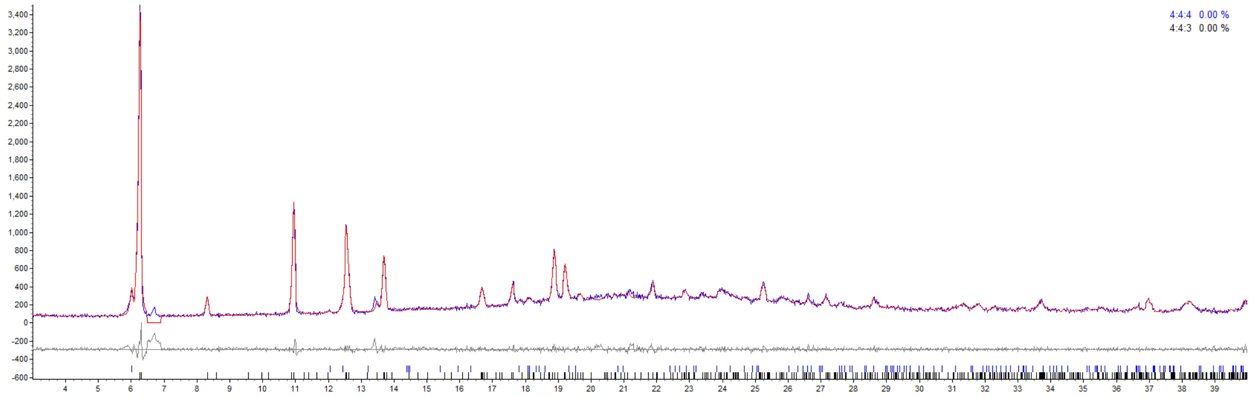

Figure S53. Observed (blue) and calculated (red) profiles and difference plot [I_{obs}-I_{calc}] (grey) of the Pawley refinement. (2θ range 3 - 40 °, d_{min} = 2.25 Å). Blue and black tickmarks correspond to reflections for **3** and **10**, respectively.

After 16 hours 15 mins at 433 K.

The sample was kept at 433 K for a further 1 hour and 30 minutes and the pattern was recorded again. The peak corresponding to some unknown phase at 2θ 6.69° remained and was excluded from refinement. The unit cells of both the 4:4:4 phase (**3**) and the 4:4:3 phase (**10**) from the previous refinement were used as the starting point for a mixed-phase Pawley refinement, employing 649 parameters (14 background, 1 zero error, 9 profile, 8 cell, 617 reflections), resulting in final indices of fit R_{wp} = 0.0828, $R_{wp'}$ = 0.1913 (Figure S54). [**(3):** *a* = 14.762 (3) Å, *b* = 6.774 (2) Å, *c* = 14.290 (3) Å, β = 96.6 (2) °, *V* = 1419.6 (6) Å^3; **(10):** *a* **=** 11.2534 (10) Å, *b* = 16.1509 (9) Å, *c* = 28.1968 (13) Å, β = 92.547 (6) °, *V* = 5119.8 (6) Å^3].

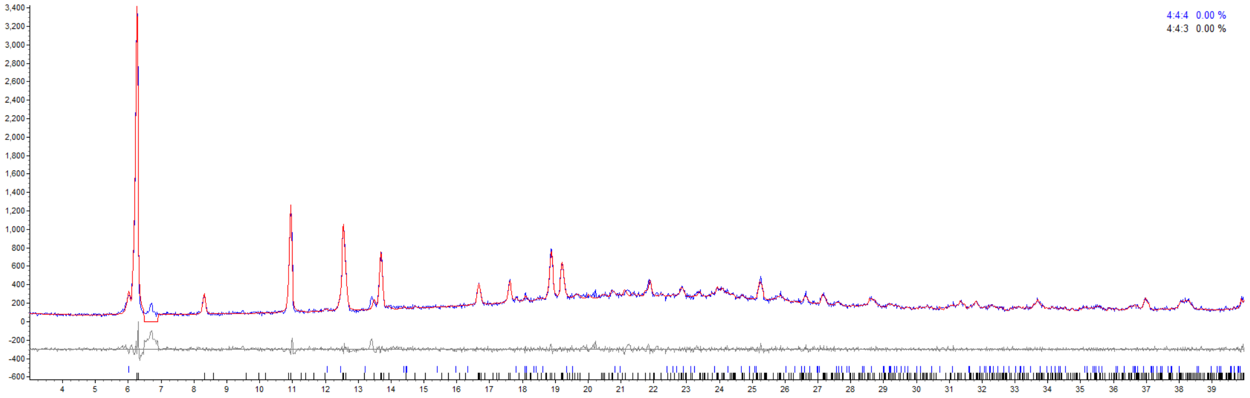

Figure S54. Observed (blue) and calculated (red) profiles and difference plot [I_{obs}-I_{calc}] (grey) of the Pawley refinement. (2θ range 3 - 40 °, d_{min} = 2.25 Å). Blue and black tickmarks correspond to reflections for **3** and **10**, respectively.

After 18 hours 30 mins at 433 K

The sample was kept at 433 K for another 18 hour and 30 minutes and the patterns were recorded again. The peak corresponding to the unknown phase at 2θ = 6.69° remained and was excluded from refinement. The unit cell of the 4:4:3 phase (**10**) from the previous refinement was used as the starting point for a Pawley refinement, employing 503 parameters (8 background, 1 zero error, 5 profile, 4 cell, 485 reflections), resulting in final indices of fit R_{wp} = 0.0876, $R_{wp'}$ = 0.205 (Figure S55). [**(10):** *a* = 11.3076(13) Å, *b* = 16.1428(9) Å, *c* = 28.1989(14) Å, β = 91.887(8) °, *V* = 5144.5(7) Å^3].

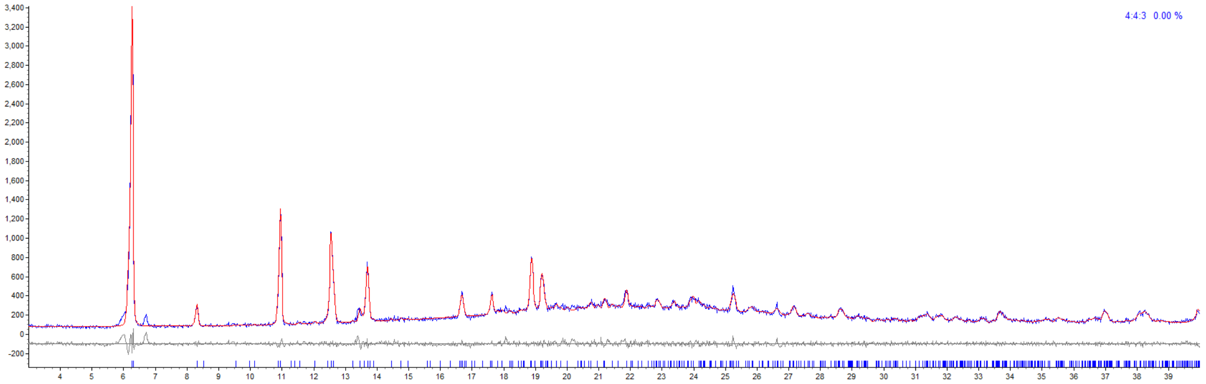

Figure S55. Observed (blue) and calculated (red) profiles and difference plot [I_{obs}-I_{calc}] (grey) of the Pawley refinement. (2θ range 3 - 40°, d_{min} = 2.25 Å).

After returning to 298 K

The sample was allowed to cool back to 298 K and maintained at 298 K for four hours before recording the pattern again. The peak corresponding to the unknown phase at 2θ = 6.69 ° remained and was excluded from the refinement. The pattern was indexed and a unit cell resembling that of **10** (as determined by single-crystal X-ray diffraction at 150 K) was identified and used as the starting point for a Pawley refinement, employing 507 parameters (10 background, 1 zero error, 5 profile, 4 cell, 487 reflections), resulting in final indices of fit R_{wp} = 0.0709, $R_{wp'}$ = 0.199 (Figure S56). [**(10):** *a* = 11.2892(8) Å, *b* = 16.1561(9) Å, *c* = 28.203(2) Å, β = 92.629(7) °, *V* = 5138.4(6) Å^3].

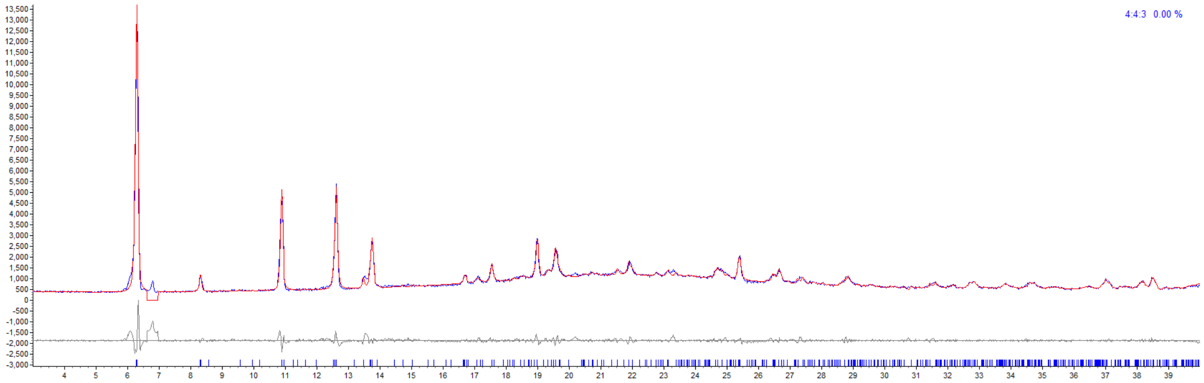

Figure S56. Observed (blue) and calculated (red) profiles and difference plot [I_{obs}-I_{calc}] (grey) of the Pawley refinement. (2*θ* range 3 - 40°, d_{min} = 2.25 Å).

$[Ag_4(O_2C(CF_2)_4CF_3)_4(quin)_4]$ (5) → 5-HT^A → 5-HT^B → unknown phase(s):

Isothermal study at 433 K: A stack plot of the series of PXRD patterns is shown in Figure 9a in the main paper for the study of isothermal heating of **5** that is analogous to the *in situ* PXRD studies of **1** and **3** that documented their conversion into the 4:4:3 phase analogues (**8** and **10**) via loss of quinoxaline. For the measurements at 298 K, scans were collected in the range of $3 \leq 2\theta \leq 40°$ using a step size of 0.015° and step time of 1.0 s, giving a total exposure time of 58 mins. No scans were collected during heating to 433 K. For the measurements at 433 K, scans were collected in the range of $3 \leq 2\theta \leq 40°$ using a step size of 0.015° and step time of 0.5 s, giving a total exposure time of 21 mins. Unlike the studies of **1** and **3**, heating of **5** to 433 K leads to a phase change to **5-HT^B**. Continued heating indicated emergence of a new phase (or phases). Peaks for this phase (or phases) were not able to be indexed, although it is plausible to suggest that the final phase may represent conversion of **5-HT^B** into a 4:4:3 phase material of composition $[Ag_4(O_2C(CF_2)_4CF_3)_4(quin)_3]$. Thus, meaningful fitting of patterns was only feasible for the first 4 patterns measured (Figures S57-S60), beyond which the new unindexed phases became dominant.

Initial pattern at 298 K

The pattern was indexed and a unit cell resembling that of **5** (obtained from single-crystal X-ray data at 150 K) was identified. Unit cell parameters from SCXRD were used as the starting point for a Pawley refinement, employing 177 parameters (10 background, 1 zero error, 5 profile, 4 cell, 157 reflections), resulting in final indices of fit $R_{wp} = 0.0507$, $R_{wp'} = 0.161$ (Figure S57). [**(5):** a = 17.246(2) Å, b = 6.8761(6) Å, c = 14.4452(8) Å, β = 95.146(10) °, V = 1706.1(2) Å^3].

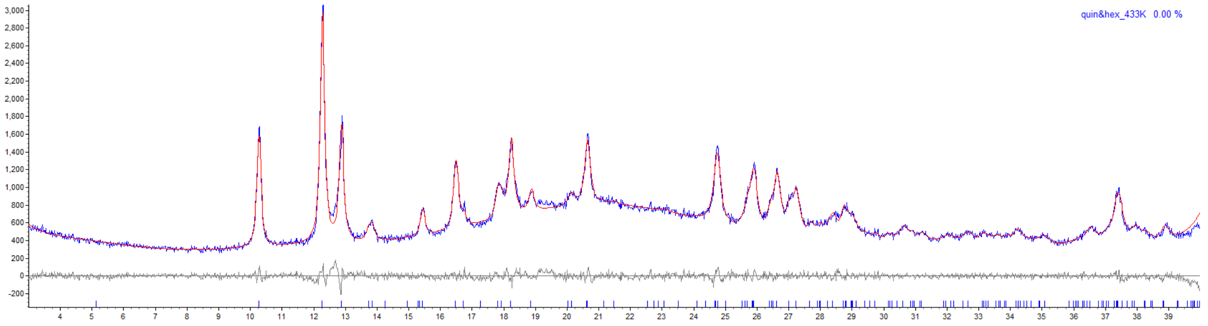

Figure S57. Observed (blue) and calculated (red) profiles and difference plot [I_{obs}-I_{calc}] (grey) of the Pawley refinement. (2θ range 3 - 40°, d_{min} = 2.25 Å).

Sample upon reaching 433 K

The sample was heated to 433 K at a rate of 360°C hr^{-1}, and when the sample reached 433 K, the diffraction pattern was immediately recorded. Visual inspection of the data indicated that the material had transformed into a new crystalline phase. The pattern was indexed to give a unit cell was which did not match or resemble any known phases for either 4:4:4 phase (**5**) or any 4:4:3 phase materials (e.g. **8-10**). The indexed unit cell was used as the starting point for a Pawley refinement, employing 90 parameters (15 background, 1 zero error, 5 profile, 3 cell, 66 reflections), resulting in final indices of fit $R_{wp} = 0.0440$, $R_{wp'} = 0.138$ (Figure S58). [**(5-HT^B):** a = 35.580(1) Å, b = 7.3470(3) Å, c = 7.020(2) Å, V = 1835.0(1) Å^3]. Attempts to determine the structure of this unknown phase (herein referred to as **5-HT^B**) by direct space methods using simulated annealing were unsuccessful.

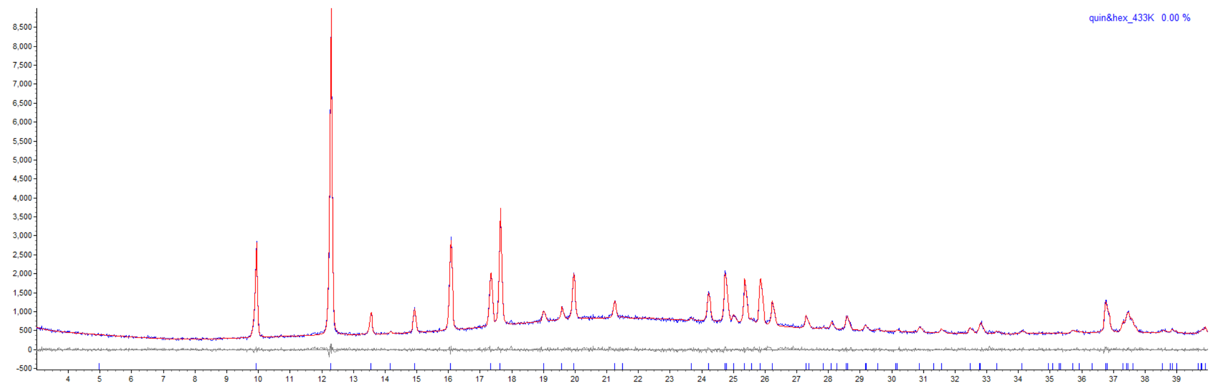

Figure S58. Observed (blue) and calculated (red) profiles and difference plot [I_{obs}-I_{calc}] (grey) of the Pawley refinement. (2θ range 3 - 40°, d_{min} = 2.25 Å).

After 1 hour and 15 mins at 433 K

The sample was kept at 433 K for a further one hour and 15 mins, and the diffraction pattern was recorded again. Visual inspection of the data indicated the growth of new broad peaks corresponding to another new crystalline phase (or phases), which again did not match or resemble any known phase for the 4:4:4 or 4:4:3 composition materials. The new peaks could not be successfully indexed to a single unit cell (even at later points in the study where they become more prominent and numerous). The unit cell of **5-HT^{B}** from the previous refinement was used as the starting point for a Pawley refinement, employing 82 parameters (8 background, 1 zero error, 5 profile, 3 cell, 65 reflections), resulting in final indices of fit R_{wp} = 0.0808, $R_{wp'}$ = 0.324 (Figure S59). [**(5-HT^{B}):** *a* = 35.577(4) Å, *b* = 7.3464(7) Å, *c* = 7.0180(6) Å, *V* = 1834.3 (3) Å^3]. These indices of fit are necessarily high as the new phase is not included in the fit.

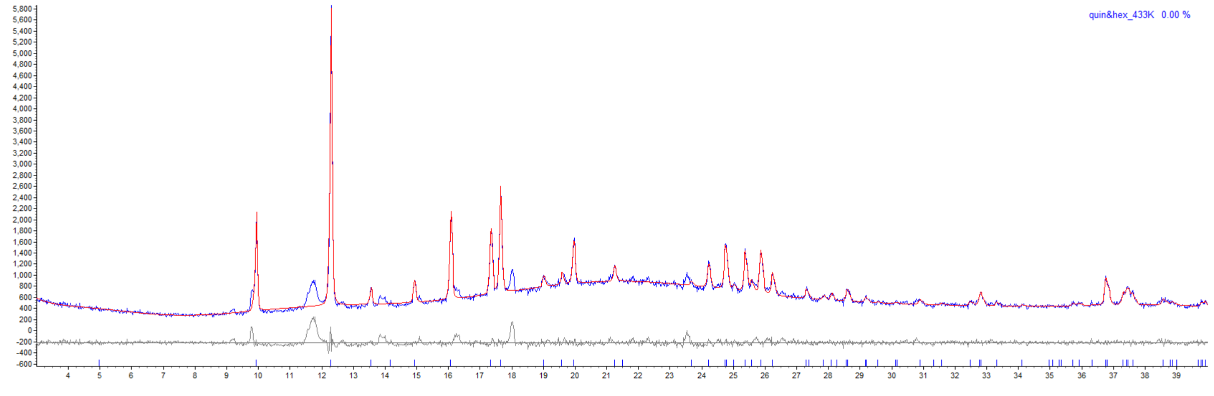

Figure 59 Observed (blue) and calculated (red) profiles and difference plot [I_{obs}-I_{calc}] (grey) of the Pawley refinement. (2θ range 3 - 40°, d_{min} = 2.25 Å).

After 2 hours and 30 mins at 433 K

The sample was held at 433 K for a further one hour and 15 mins, and the diffraction pattern was recorded again. Peaks corresponding to the new un-indexed crystalline phase(s) developed further, and those corresponding to **5-HT^{B}** further decreased in intensity. The unit cell of **5-HT^{B}** derived from the previous refinements was used as the starting point for a Pawley refinement, employing 82 parameters (9 background, 1 zero error, 5 profile, 3 cell, 64 reflections), resulting in final indices of fit R_{wp} = 0.126, $R_{wp'}$ = 0.614 (Figure S60). [**(5-HT^{B})**: *a* = 35.56(1) Å, *b* = 7.344(2) Å, *c* = 7.016(2) Å, *V* = 1832.4(8) Å^3]. These indices of fit are necessarily higher than that for the previous pattern as the new phase is not included in the fit.

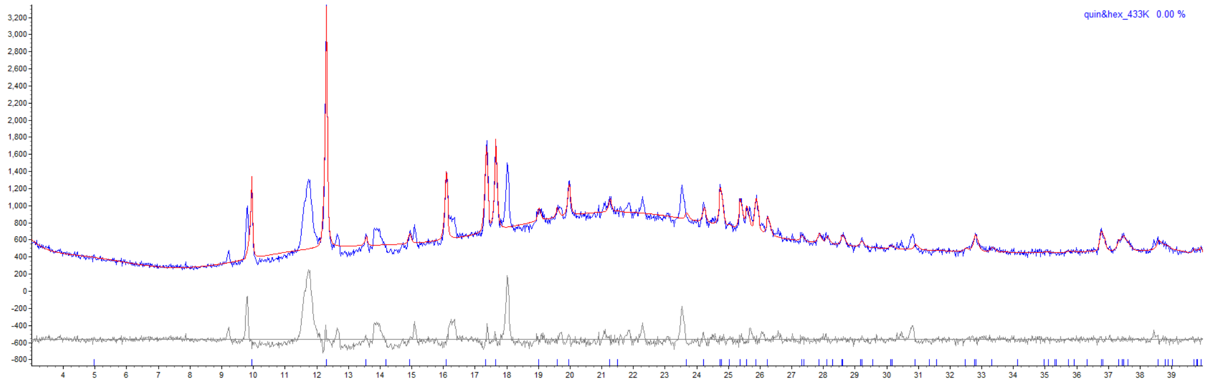

Figure S60. Observed (blue) and calculated (red) profiles and difference plot [I_{obs}-I_{calc}] (grey) of the Pawley refinement. (2θ range 9 - 40°, d_{min} = 2.25 Å).

Overlap of peaks corresponding to the **5-HT^{B}** and the new un-indexed crystalline phase(s) meant that further meaningful refinement of the unit cell parameters of **5-HT^{B}** was no longer possible. As such, further fitting was not performed on subsequent diffraction patterns in this study.

<u>Stepwise heating study 293K→433K:</u> In a separate study a sample of **5** was heated from 293 K to 433 K in steps of 20 K, using a heating rate for each step of 360 °C hr^{-1}. A pattern was measured after each 20 K temperature rise while the temperature was held constant during the measurement. Each scan was collected in the range $3 \leq 2\theta \leq 25°$ using a step size of 0.015° and step time of 0.1 sec, giving a total exposure time of 58 mins. A stack plot of these patterns is shown in Figure 9b of the main paper. The data were compared to the PXRD patterns for **5** and **5-HT^{B}** and other phases were indexed where possible. Pawley fitting of the patterns as single- or mixed-phase fits were then undertaken. The results show a progression of phase changes from **5 → 5-HT^{A} → 5-HT^{B}**, with subsequent emergence of a new phase at the highest temperature (433 K). Fits of key patterns are shown in Figures S61-S63.

Pattern at 373 K

The pattern was indexed to a single phase with a new unit cell that was different from that of **5** and **5-HT^{B}**; this new phase will be referred to as **5-HT^{A}**. This indexed unit cell was used as the starting point for a Pawley refinement, employing 47 parameters (15 background, 1 zero error, 5 profile, 3 cell, 23 reflections), resulting in final indices of fit R_{wp} = 0.0571, $R_{wp'}$ = 0.107 (Figure S61). [**(5-HT^{A})**: *a* = 7.3301(3) Å, *b* = 34.939(2) Å, *c* = 6.9508(4) Å, β = 87.01(1) °, *V* = 1777.7(2) Å^{3}].

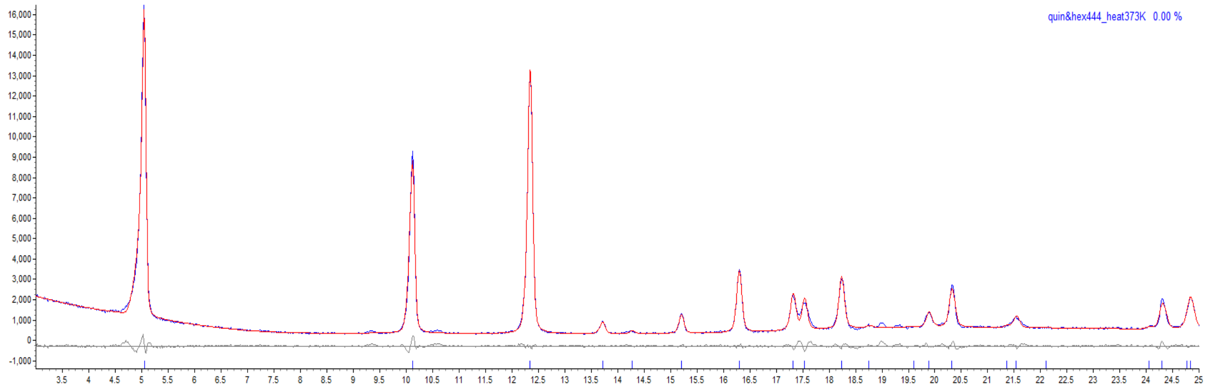

Figure S61. Observed (blue) and calculated (red) profiles and difference plot [I_{obs}-I_{calc}] (grey) of the Pawley refinement. (2θ range 3 - 25°, d_{min} = 3.56 Å).

Pattern at 413 K

The unit cell of **5-HT**$^{\mathbf{B}}$ was used as the starting point for a Pawley refinement, employing 47 parameters (15 background, 1 zero error, 5 profile, 3 cell, 23 reflections), resulting in final indices of fit R_{wp} = 0.0547, $R_{wp'}$ = 0.102 (Figure S62). [**(5-HT**$^{\mathbf{B}}$**)**: a = 35.4334(9) Å, b = 7.3325(2) Å, c = 6.9834(3) Å, V = 1814.4(1) Å^3].

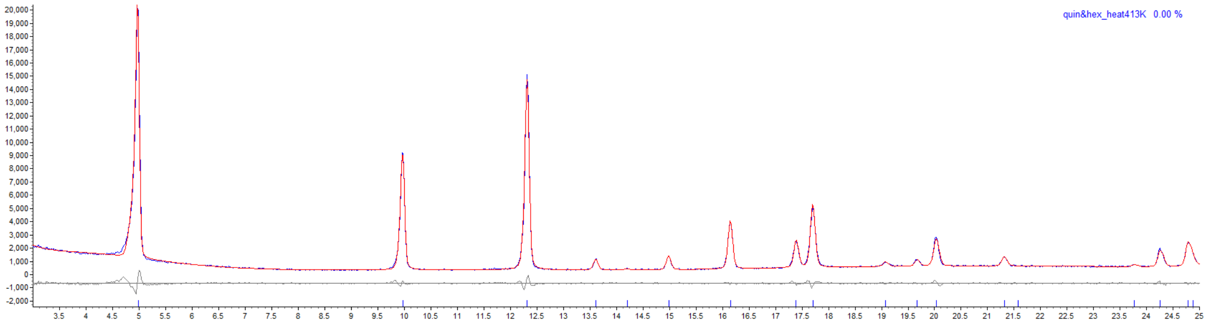

Figure S62 Observed (blue) and calculated (red) profiles and difference plot [I_{obs}-I_{calc}] (grey) of the Pawley refinement. (2θ range 3 - 25°, d_{min} = 3.56 Å).

Pattern at 433 K

The unit cell of **5-HT**$^{\mathbf{B}}$ from the previous refinement was used as the starting point for a Pawley refinement, employing 47 parameters (15 background, 1 zero error, 5 profile, 3 cell, 23 reflections), resulting in final indices of fit R_{wp} = 0.106, $R_{wp'}$ = 0.208 (Figure S63). [**(5-HT**$^{\mathbf{B}}$**)**: a = 35.559(4) Å, b = 7.342(6) Å, c = 7.011(8) Å, V = 1830.3(3) Å^3]. Some new peaks were increasing in intensity around 4.75 ° and 11.75 ° which no longer fitted to the **5-HT**$^{\mathbf{B}}$ phase suggesting that a further transformation may be beginning.

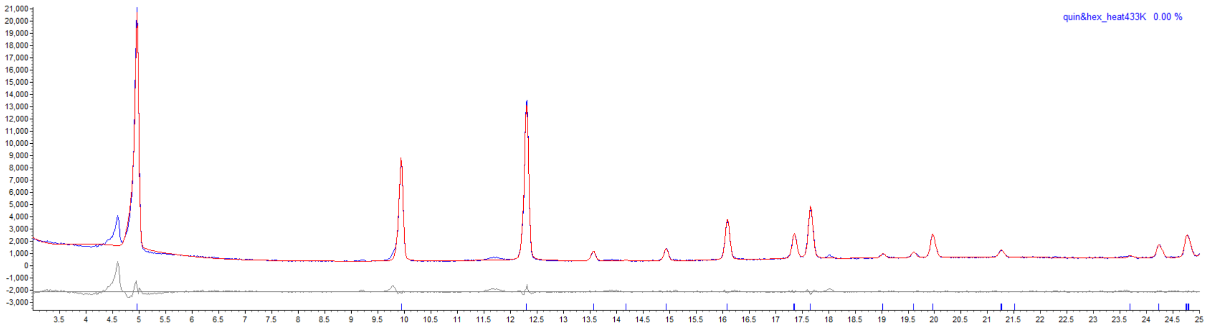

Figure S63. Observed (blue) and calculated (red) profiles and difference plot [I_{obs}-I_{calc}] (grey) of the Pawley refinement. (2θ range 3 - 25°, d_{min} = 3.56 Å).

S7. Diffraction studies of thermal expansion and phase change for 1, 3 and 5

The thermal expansion of compounds **1**, **3** and **5** is illustrated in Figure 10 in the main paper, allowing the observed behaviour to be interpreted in the context of the mechanism for conversion of 4:4:4 phase materials to 4:4:3 phase materials via extrusion of quinoxaline into

the vapour phase. The unit cell data used in this analysis (and in Figure 10) are presented in Tables S7 (for **1**), S8 (for **3**) and S9 (for **5**, **5-HT^{A}**, **5-HT^{B}**).

Table S7. Unit cell parameters for **1** as a function of temperature.

	**1** (150 K)	**1** (298 K)	**1** (433 K)
Space gp	*Pbcm*	*Pbcm*	*Pbcm*
a (Å)	11.7575(3)	11.7080(7)	11.848(2)
b (Å)	6.6483(2)	6.7367(7)	6.783(2)
c (Å)	14.4311(4)	14.6158(9)	14.620(5)
α (°)	90	90	90
β (°)	90	90	90
γ (°)	90	90	90
V (Å^3)	1128.04(5)	1152.8(2)	1174.8(5)

Table S8. Unit cell parameters for **3** as a function of temperature.

	**3** (150 K)	**3** (298 K)	**3** (433 K)
Space gp	$P2_1/c$	$P2_1/c$	$P2_1/c$
a (Å)	14.5530(7)	14.6269(11)	14.937(2)
b (Å)	6.6729(4)	6.7864(6)	6.755(2)
c (Å)	14.3152(6)	14.3807(9)	14.441(2)
α (°)	90	90	90
β (°)	96.466(3)	96.82(1)	100.66(3)
γ (°)	90	90	90
V (Å^3)	1381.32(12)	1417.4(2)	1432.1(5)

Table S9. Unit cell parameters for **5** as a function of temperature including conversion to **5-HT^A^** and **5-HT^B^**.

	**5** (150 K)	**5** (298 K)	**5-HT^{A}** (373 K)	**5-HT^{B}** (413 K)
Space gp	$P2_1/c$	$P2_1/c$	Bb	$B22_12$
a (Å)	17.0199(16)	17.247(2)	34.940(2)	35.433(1)
b (Å)	6.7461(6)	6.8735(7)	6.9508(5)	6.9834(3)
c (Å)	14.3243(16)	14.452(1)	7.3302(3)	7.3325(2)
α (°)	90	90	90	90
β (°)	94.823(7)	95.13(1)	90	90
γ (°)	90	90	87.007(6)	90
V (Å^3)	1638.86(3)	1706.4(3)	1777.7(2)	1814.4(1)

S8. Dielectric constant test

Samples (0.1g each) were ground into powders and compressed into round plates (d = 13 mm) by using IR plate moulds. Both upper and lower surfaces of samples were coated with silver paint and dried before measurements to smooth the surface of the plates and minimise errors in measurements. Dielectric constants and losses at different frequencies (1-10^6 Hz) were measured on a broadband dielectric spectrometer (Novocontrol Concept 80, Germany) in Soochow University, China.

S9. CIF A and B alerts

Compound 2:

Alert A: Low $\sin\theta/\lambda_{max}$ - crystals are very thin plates and diffract weakly.

Alert B: low ratio of reflections to parameters – a consequence of weak diffraction leading to smaller than ideally desirable number of observable reflections.

Compound 5

Alert A: isotropic non-H atoms in main residue(s) – due to disorder model for perfluoalkyl chains

Alert B: Large range of U_{eq}(max)/U_{eq}(min) for C and F elements – due to disorder modelling of perfluoralkyl chains.

Compound 8

Alert A: Low $\sin\theta/\lambda_{max}$ – crystals are very thin plates and diffract weakly.Alert B: low ratio of reflections to parameters – a consequence of weak diffraction leading to smaller than ideally desirable number of observable reflections.

Alert B: isotropic non-H atoms in main residue(s) – due to disorder model for perfluoalkyl chains

Alert B: large Hirschfeld differences C3-C4 – due to disorder model for perfluoalkyl chains and uncertainties in displacement parameter magnitudes as a consequence of uncertainty in site occupancy.

Alert B: Low bond precision C-C bonds – due to disorder model for perfluoalkyl chains

Compound 9

Alert A: Low $\sin\theta/\lambda_{max}$ – crystals are very thin plates and diffract weakly.

Alert A: low ratio of reflections to parameters – a consequence of weak diffraction leading to smaller than ideally desirable number of observable reflections.

Alert B: High U_{eq} compared to neighbours (C6) due to disorder model for perfluoromethyl group

Compound 10

Alert A: isotropic non-H atoms in main residue(s) – due to disorder model for perfluoalkyl chains

Alert B: Large range of U_{eq}(max)/U_{eq}(min) for C atoms – due to disorder modelling of perfluoralkyl chains.

S10. References

S1 G. M. Sheldrick, *Acta Crystallogr.*, **2008**, *64*, 112–122.

S2 O. V Dolomanov, L. J. Bourhis, R. J. Gildea, J. A. K. Howard and H. Puschmann, *J. Appl. Crystallogr.*, **2009**, 42, 339-341.

S3 (a) *SADABS*, empirical adsorption correction program,S4b based on the method of Blessing.[S4] (b) L. Krause, R. Herbst-Irmer, G. M. Sheldrick and D. Stalke, *J. Appl. Cryst.* **2015**, *48*, 3–10.

S4 R. H. Blessing, *Acta Crystallogr. Sect. A*, **1995**, *51*, 33–38.

S5 (a) S. P. Thompson, J. E. Parker, J. Potter, T. P. Hill, A. Birt, T. M. Cobb, F. Yuan and C. C. Tang, *Rev. Sci. Instrum.* **2009**, *80*, 075107. (b) S. P. Thompson, J. E. Parker, J. Marchal, J. Potter, A. Birt, F. Yuan, R. D. Fearn, A. R. Lennie, S. R. Street and C. C. Tang, *J. Synchrotron Radiat.* **2011**, *18*, 637–648.

S6 G. S. Pawley, *J. Appl. Crystallogr.*, 1981, 14, 357–361.

S7 A. A. Coelho, *TOPAS Academic Version 4.1, 2007, see http//www.topas-academic.net.* (b) A. A. Coelho, *J. Appl. Cryst.*, **2018**, *51*, 210–218; (c) A. A. Coelho, J. Evans, I. Evans, A. Kern and S. Parsons, *Powder Diffr.*, **2011**, *26*, S22–S25.

S8 H. M. Rietveld, *J. Appl. Crystallogr.*, 1969, **2**, 65–71.

Open Access: For the purpose of open access, the authors have applied a Creative Commons Attribution (CC BY) licence to any Author Accepted Manuscript version arising.
